# Nutraceuticals for the management of weight and inflammation-related complications in obesity: a pediatric perspective. Systematic review and network meta-analysis

**DOI:** 10.3389/fnut.2026.1715574

**Published:** 2026-02-12

**Authors:** Gianvincenzo Zuccotti, Alessandro Gatti, Virginia Rossi, Erika Cordaro, Valeria Calcaterra

**Affiliations:** 1 Department of Biomedical and Clinical Sciences, University of Milan, Milan, Italy; 2 Pediatric Department, Buzzi Children's Hospital, Milano, Italy; 3 Laboratory of Adapted Motor Activity (LAMA), Department of Public Health, Experimental Medicine and Forensic Science, University of Pavia, Pavia, Italy; 4 National PhD Programme in One Health Approaches to Infectious Diseases and Life Science Research, Department of Public Health, Experimental and Forensic Medicine, University of Pavia, Pavia, Italy; 5 Department of Internal Medicine and Therapeutics, University of Pavia, Pavia, Italy

**Keywords:** butyrate, carnitine, inulin, long-chain omega-3 fatty acids, nutraceuticals, obesity, pediatrics, vitamin B

## Abstract

**Background:**

Obesity, defined as excess body fat that impairs health, is a major public health challenge associated with metabolic and inflammation-related complications across the lifespan. Conventional treatments often show limited long-term efficacy, leading to growing interest in complementary strategies. Nutraceuticals have been studied for their potential in weight management and metabolic improvement. This systematic review and network meta-analysis evaluates the role of nutraceuticals in obesity management, with attention also given to pediatric populations.

**Methods:**

We performed a systematic review and network meta-analysis (NMA) following Cochrane and PRISMA-NMA 2020 guidelines. Eligible randomized and non-randomized trials enrolled children or adults with overweight/obesity, testing nutraceuticals (inulin, butyrate, long-chain omega-3 fatty acids, vitamin B, carnitine) versus placebo or standard care. Primary outcomes included anthropometric, metabolic, and inflammatory markers. The protocol was registered in PROSPERO (ID: CRD420251151333).

**Results:**

L-carnitine emerged as the most effective and consistent intervention, producing significant reductions in body weight, body mass index (BMI), waist circumference, fasting blood glucose, Homeostatic Model Assessment for Insulin Resistance (HOMA-IR), and LDL-Cholesterol (LDL-C), along with a significant increase in HDL-Cholesterol (HDL-C). Inulin and long-chain omega-3 fatty acids (LC n3-PUFA) exhibited modest or non-significant effects on most outcomes, although LC n3-PUFA significantly reduced triglyceride levels. Butyrate demonstrated beneficial effects on BMI and waist circumference in children, whereas vitamin B showed limited impact. Dose–response analyses confirmed the efficacy of L-carnitine at relatively low dosages, while other supplements required higher intakes without achieving the predefined clinical targets.

**Conclusion:**

This NMA shows heterogeneous effects of nutraceuticals on obesity-related outcomes. L-carnitine emerged as the most consistent intervention, while LC n3-PUFA, inulin, butyrate, and vitamin B provided more limited benefits. Preliminary evidence suggests potential age-related differences, highlighting the need for further studies to define age-specific and tailored strategies for obesity management.

## 1 Introduction

Obesity, defined as an abnormal or excessive fat accumulation that poses a risk to health, represents one of the most critical global public health concerns, with its prevalence rising steadily in both developed and developing countries (1, 2). According to the World Health Organization (WHO), in 2022, one in eight people worldwide were living with obesity (1). Since 1990, global adult obesity has more than doubled, while the prevalence of obesity among adolescents has quadrupled (1). Current estimates indicate that in 2022 approximately 2.5 billion adults aged 18 years and older were overweight, of whom 890 million were living with obesity. Overall, 43% of adults were classified as overweight and 16% as obese (1). Childhood obesity also remains a major concern: in 2024, around 35 million children under the age of five were overweight, while in 2022 more than 390 million children and adolescents aged 5–19 years were overweight, including 160 million living with obesity (1). The growing burden of childhood obesity is particularly alarming, as it predisposes individuals to long-term cardiometabolic complications, including type 2 diabetes mellitus (T2DM), hypertension, dyslipidemia, non-alcoholic fatty liver disease (NAFLD), and systemic inflammatory conditions (3–7).

**Figure 1:**
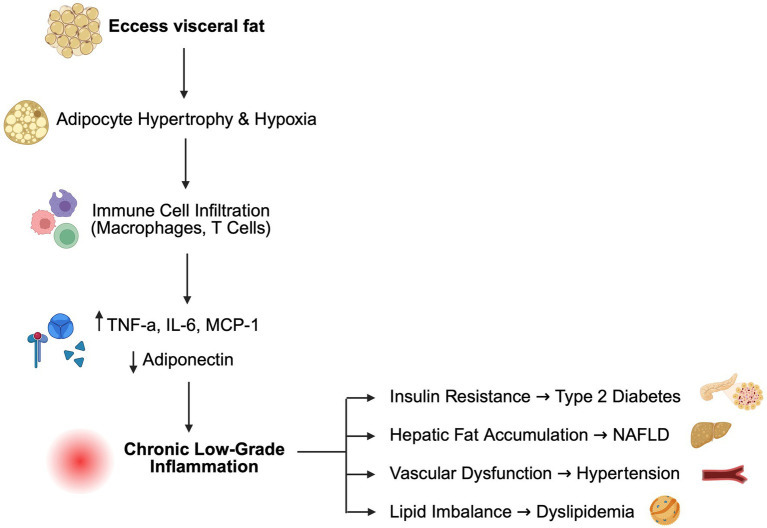
Mechanistic link between excess adiposity, inflammation, and cardiometabolic disease. Excess visceral fat leads to adipocyte hypertrophy and hypoxia, promoting infiltration of immune cells such as macrophages and T lymphocytes. These cells secrete pro-inflammatory cytokines tumor necrosis factor-alpha (TNF-α) and interleukin-6 (IL-6), monocyte chemoattractant protein-1 (MCP-1) and suppress adiponectin, creating chronic low-grade inflammation. This inflammatory state contributes to key obesity-related complications, including insulin resistance and type 2 diabetes, hepatic fat accumulation leading to non-alcoholic fatty liver disease (NAFLD), vascular endothelial dysfunction leading to hypertension, and lipid imbalance resulting in dyslipidemia.

The mechanisms linking obesity to these long-term complications are multifactorial. At the pathophysiological level, obesity is characterized not only by excessive adipose tissue accumulation but also by a state of chronic low-grade inflammation directly driven by weight excess and adipose tissue expansion (8–10). As adipocytes enlarge, hypoxia, mechanical stress, and endoplasmic reticulum stress develop within adipose tissue, contributing to adipocyte dysfunction, increasing lipolysis and elevating circulating free fatty acids, which impair insulin signaling and contribute to insulin resistance, a core driver of type 2 diabetes mellitus (T2DM) (11, 12), Figure 1.

Excess weight also activates an inflammatory cascade: hypertrophic adipocytes release chemotactic signals that recruit macrophages and T lymphocytes into adipose depots, where immune cells shift toward a pro-inflammatory phenotype. These infiltrating immune cells, together with stressed adipocytes, secrete cytokines such as TNF-*α*, IL-6, and MCP-1 while reducing protective adipokines such as adiponectin, establishing chronic systemic inflammation (11–13). These mediators extend beyond adipose depots, propagating systemic inflammation, endothelial dysfunction, and oxidative stress. Such processes promote hypertension through increased sympathetic activity and renin–angiotensin–aldosterone system (RAAS) activation, while dyslipidemia arises from altered hepatic lipid metabolism and increased very-low-density lipoprotein synthesis synthesis (13, 14). Excess fat deposition in the liver further contributes to the development of NAFLD, which may progress to steatohepatitis and fibrosis (14). Finally, obesity-associated changes in gut microbiota composition and impaired intestinal barrier integrity allow translocation of lipopolysaccharides (LPS) into the bloodstream, metabolic endotoxemia, which further amplifies inflammation and reinforces the bidirectional relationship between adipose tissue dysfunction and systemic metabolic impairment (15, 16).

Conventional management strategies for obesity include dietary restriction, lifestyle modifications, and behavioral interventions, although they often show limited long-term adherence and efficacy (17, 18). Dietary restriction typically involves calorie reduction and structured eating patterns, ranging from low-calorie and very-low-calorie diets to macronutrient-specific approaches such as low-carbohydrate, Mediterranean, or low-fat diets, aimed at inducing a sustained negative energy balance (19–21). Lifestyle modifications encompass increased physical activity and structured exercise programs, including aerobic training, resistance training, and combined regimens, which have been shown to improve metabolic health and support weight loss maintenance (22–25). Behavioral interventions target psychological and cognitive aspects of eating and activity patterns, incorporating techniques such as cognitive-behavioral therapy, goal-setting, motivational interviewing, self-monitoring (e.g., food diaries, wearable activity trackers), and relapse-prevention strategies to improve adherence and establish long-term healthy habits (26–28). Despite their clinical relevance, these conventional approaches often suffer from low long-term adherence due to barriers such as palatability restrictions, environmental influences, and behavioral fatigue (29–31). For this reason, increasing attention has been directed toward nutraceuticals and functional foods as adjunctive tools in obesity prevention and treatment.

Among the various nutraceuticals under investigation, several have shown promising potential in the management of obesity and its related complications. Prebiotic fibers, such as inulin, are of particular interest because they selectively stimulate the growth of beneficial gut bacteria and promote the production of short-chain fatty acids, including butyrate, acetate, and propionate (32–36). These metabolites not only improve intestinal barrier function and reduce endotoxemia, but also act as signaling molecules influencing glucose and lipid metabolism, appetite regulation, and systemic inflammation. Mechanistically, inulin reaches the colon undigested, where it undergoes fermentation by commensal bacteria, particularly *Bifidobacterium* and *Lactobacillus* species. This process generates SCFAs that stimulate enteroendocrine L-cells to release glucagon-like peptide-1 (GLP-1) and peptide YY (PYY), hormones known to enhance satiety, reduce appetite, and improve insulin sensitivity; SCFAs also strengthen intestinal tight junctions, lowering metabolic endotoxemia and systemic inflammation while contributing to improved gut–brain axis signaling and central appetite regulation (29, 37). Through these effects, inulin may help to restore gut–brain axis communication and support long-term weight management (38).

Omega-3 fatty acids represent another well-established category of nutraceuticals with broad metabolic benefits (39). Their anti-inflammatory properties derive from their ability to compete with arachidonic acid in eicosanoid synthesis, leading to the production of less pro-inflammatory lipid mediators (40–42). Furthermore, omega-3 fatty acids are known to improve endothelial function, reduce plasma triglyceride levels, and enhance insulin sensitivity, thereby lowering cardiometabolic risk in obese individuals (43). Other compounds, such as L-carnitine and B-complex vitamins, have been studied in the context of energy metabolism and mitochondrial function. L-carnitine plays a crucial role in shuttling long-chain fatty acids into mitochondria for *β*-oxidation, which can increase energy expenditure and reduce fat storage (44–46). B vitamins, particularly B1, B6, B9, and B12, serve as essential cofactors in enzymatic pathways that regulate carbohydrate and lipid metabolism, supporting metabolic flexibility and helping counteract the oxidative stress commonly associated with obesity (47, 48).

Collectively, these findings support the role of nutraceutical interventions, alone or in combination with lifestyle modifications, as a promising multifaceted approach to address not only weight control but also the inflammatory and metabolic disturbances underlying obesity. Nutraceuticals may assume an increasingly important role within the framework of personalized nutrition and precision medicine (49). Individual variability related to genetics, metabolic phenotype, and gut microbiota composition strongly influences the response to bioactive compounds, highlighting the value of targeted interventions (38, 50, 51). Moreover, the combination of different nutraceuticals could provide synergistic benefits by simultaneously acting on appetite regulation, energy metabolism, and inflammation. This perspective is particularly valuable in pediatrics, where early interventions are crucial to prevent the progression of childhood obesity into adulthood and to reduce the lifelong risk of cardiometabolic complications (52–54). The integration of nutraceuticals with lifestyle modifications may therefore represent a sustainable and safe strategy to promote healthy growth and to protect long-term metabolic health in children and adolescents.

Despite encouraging findings, current evidence remains fragmented, as existing reviews generally assess nutraceuticals in isolation, with narrow compound focus, limited outcomes, and restricted populations, seldom including pediatric groups. To date, no synthesis has compared multiple interventions within a unified framework, nor integrated comparative efficacy with dose–response analyses to determine optimal intake levels and clinical thresholds.

The aim of this systematic review and network meta-analysis is to evaluate the role of nutraceutical interventions in obesity management, incorporating evidence from both adult and pediatric populations. This approach allows a more comprehensive understanding of their potential application in childhood, where research remains comparatively limited despite the lifelong impact of early intervention. Considering various nutraceuticals, particularly inulin, butyrate, long-chain omega-3 fatty acids (LC n3-PUFA) including eicosapentaenoic acid (EPA) and docosahexaenoic acid (DHA), vitamin B complexes, and carnitine, this review focuses on four main aspects: (1) the effectiveness of nutraceuticals in weight management, including the prevention and reduction of excess weight gain; (2) their impact on metabolic comorbidities including hyperinsulinemia, dyslipidemia, and hypertension, as well as biomarkers of obesity-related inflammation; (3) the comparative effectiveness of different nutraceuticals was assessed through a network meta-analysis (NMA) to identify the most promising strategies for improving anthropometric characteristics and blood biomarkers, while dose–response meta-analysis approaches were applied to evaluate the relative efficacy of interventions and (4) the specific implications and potential applications for pediatric populations.

## 2 Materials and methods

### 2.1 Study design

This systematic review and NMA was conducted according to the methodological standards outlined in the Cochrane Handbook for Systematic Reviews of Interventions (55). Findings are reported following the Preferred Reporting Items for Systematic Reviews and Meta-Analyses incorporating Network Meta-Analyses (PRISMA-NMA) 2020 statement (56) and reported in Supplementary Table S1. The review question was structured using the Population-Intervention-Comparator-Outcome (PICO) framework (Table 1), which guided both the literature search and study selection.

**Table 1:** PICO elements table.

Element	Definition
Population	Individuals with overweight or obesity (excluding pregnant women); children and adults were considered separately in subgroup analyses
Intervention	Nutraceuticals [e.g., particularly inulin, butyrate, long-chain omega-3 fatty acids (eicosapentaenoic acid, EPA and docosahexaenoic acid, DHA), vitamin B complexes, and carnitine]
Comparator	Placebo or standard care
Outcomes	(1) Weight loss or prevention of weight gain; (2) Resolution of metabolic comorbidities related to obesity (hyperinsulinemia, dyslipidemia, hypertension): (3) Improvement of biomarkers of inflammation linked to obesity
Setting	Clinical trials (randomized or non-randomized) published in peer-reviewed journals within the last 15 years

### 2.2 Protocol registration

The review protocol was prospectively registered in the International Prospective Register of Systematic Reviews (PROSPERO) under the registration number CRD420251151333.

### 2.3 Search strategy

A comprehensive search was performed across three major electronic databases: PubMed, Scopus and Web of Science. The search covered publications from January 2010 to May 2025, to ensure inclusion of studies reflecting contemporary nutraceutical formulations, standardized diagnostic criteria, and current outcome assessment practices. Search terms included controlled vocabulary (e.g., MeSH) and free-text words related to nutraceuticals, obesity, weight management, and metabolic comorbidities. Boolean operators (AND, OR) were used to combine search terms, and filters were applied to restrict the results to human studies (example at Supplementary Table S2).

The detailed search strategy was adapted for each database. Reference lists of included studies and relevant reviews were also hand-searched to identify additional eligible articles.

### 2.4 Study selection

All references retrieved through the database search were imported into Rayyan® to facilitate systematic review management (57). Three reviewers (AG, EC and VR) independently performed the screening process, which was conducted in two stages. In the first stage, titles and abstracts were examined to determine preliminary eligibility. In the second stage, the full texts of potentially relevant articles were assessed in detail. Any disagreements that arose during screening were resolved by discussion, and when consensus could not be reached, a fourth reviewer (VC) was consulted.

Studies were considered eligible if they investigated human populations of children or adults with overweight or obesity and evaluated the effects of nutraceutical interventions. In the specific case of omega-3 trials, only those assessing formulations containing DHA and EPA were included. Eligible studies were required to report at least one of the prespecified outcomes, which comprised weight loss, prevention of weight gain, resolution or prevention of obesity-related metabolic comorbidities, or biomarkers of inflammation. To ensure clinical relevance and contemporaneity, only publications from the last 15 years were included.

Studies were excluded when they involved pregnant women, when they did not provide a comparator such as placebo, standard care, or an alternative nutraceutical, or when they consisted of reviews, case reports, editorials, or conference abstracts without full original data.

### 2.5 Data extraction

Data extraction was carried out independently by three reviewers using a standardized and piloted form to ensure consistency. For each eligible study, we recorded general information such as the author, year of publication, and country of origin, along with methodological details including study design and characteristics of the study population, for example sample size, age distribution, baseline body mass index, and the presence of metabolic comorbidities. Moreover, we systematically documented the type of nutraceutical investigated, its formulation, dosage, and the duration of treatment, together with specific protocol characteristics such as whether administration followed a daily or weekly schedule. Information regarding the comparator, whether placebo, usual care, or an alternative nutraceutical, was also carefully extracted. Outcomes were collected in relation to weight reduction, prevention of weight gain, resolution or prevention of metabolic comorbidities including hyperinsulinemia, dyslipidaemia, and hypertension, as well as biomarkers of obesity-related inflammation. In order to evaluate potential differences in treatment effects across age groups, datasets were constructed separately for studies involving adults and those involving pediatric populations, thereby allowing for specific subgroup analyses.

### 2.6 Risk of bias and assessment

The methodological quality of included studies was assessed using validated appraisal tools appropriate to study design. Randomized controlled trials were evaluated with the Cochrane Risk of Bias 2 (RoB 2) tool, while non-randomized studies were appraised with the ROBINS-I tool (55, 58). Study quality was not used as a reason for exclusion but was taken into account when interpreting the strength and reliability of the overall body of evidence. Risk of bias at the study were performed for each outcome (and, where relevant, at specific time points) with respect to the effect of assignment to the intervention (i.e., an intention-to-treat effect). Three reviewers (AG, EC and VR) conducted all assessments independently, and any disagreements were resolved by consensus or adjudication by a fourth reviewer (VC). For each study–outcome, judgments were made across the five RoB 2 domains: bias arising from the randomization process; bias due to deviations from intended interventions; bias due to missing outcome data; bias in measurement of the outcome; and bias in selection of the reported result. Decisions were guided by the tool’s signaling questions, and both domain-level and overall judgments followed the standard RoB 2 algorithm, yielding categories of low risk, some concerns, or high risk.

### 2.7 Statistical analysis

Data from randomized controlled trials were analyzed using both NMA and dose–response meta-analysis approaches to assess the comparative efficacy of interventions on anthropometric characteristics and blood biomarkers. Post-pre intervention changes were calculated for each intervention group if not reported. Mean differences (MD) were used as the summary measure when outcomes were reported using the same unit of measurement across studies, while standardized mean differences (SMD) were applied when outcomes were measured on different scales. A frequentist NMA was conducted using a random-effects model to account for between-study heterogeneity, applying inverse variance weighting for each pairwise comparison. Network geometry was visualized using network plots, with node sizes proportional to the number of participants and edge thickness proportional to the number of studies informing each comparison. Forest plots were generated to present pooled MDs with 95% confidence intervals (CIs), alongside P-scores to rank interventions according to their relative efficacy. Prediction intervals were also calculated to reflect the range of expected effects in future studies (Supplementary Table S4). Heterogeneity and inconsistency were assessed using Cochran’s *Q* test, the *I*^2^ statistic, design-by-treatment decomposition, and node-splitting methods. League tables presenting all pairwise comparisons with 95% CIs were produced. To assess small-study effects, comparison-adjusted funnel plots were generated and visually inspected. Comparison-adjusted funnel plots were also generated to assess the potential for small-study effects; these are presented in the supplementary material (from Supplementary Figures S1 to S12). Results of the node-splitting analyses are reported in the supplementary material (Supplementary Figures S16–S25).

In addition to the NMA, a dose–response meta-analysis was conducted to evaluate the relationship between supplement dose (mg/day) and weight change at a standardized timepoint of 12 weeks. Individual study arms were used to calculate mean differences from placebo, with pooled estimates computed across multi-arm trials. Separate dose–response models were fitted for each supplement using restricted maximum likelihood (REML) estimation and a multiple-doses covariance structure. Linear models were selected based on data sufficiency and convergence criteria. Predictions were made across observed dose ranges, and minimal effective doses were estimated based on both the upper bounds of 95% CIs and the predicted mean difference required to reach a clinically meaningful reduction. Results were visualized using multi-line dose–response plots with confidence intervals and threshold indicators. Subgroup analyses were performed for children. All analyses were conducted using R software (version 4.4.0), with the netmeta and dosresmeta packages used for NMA and dose–response modeling, respectively.

## 3 Results

### 3.1 Search results

**Figure 2:**
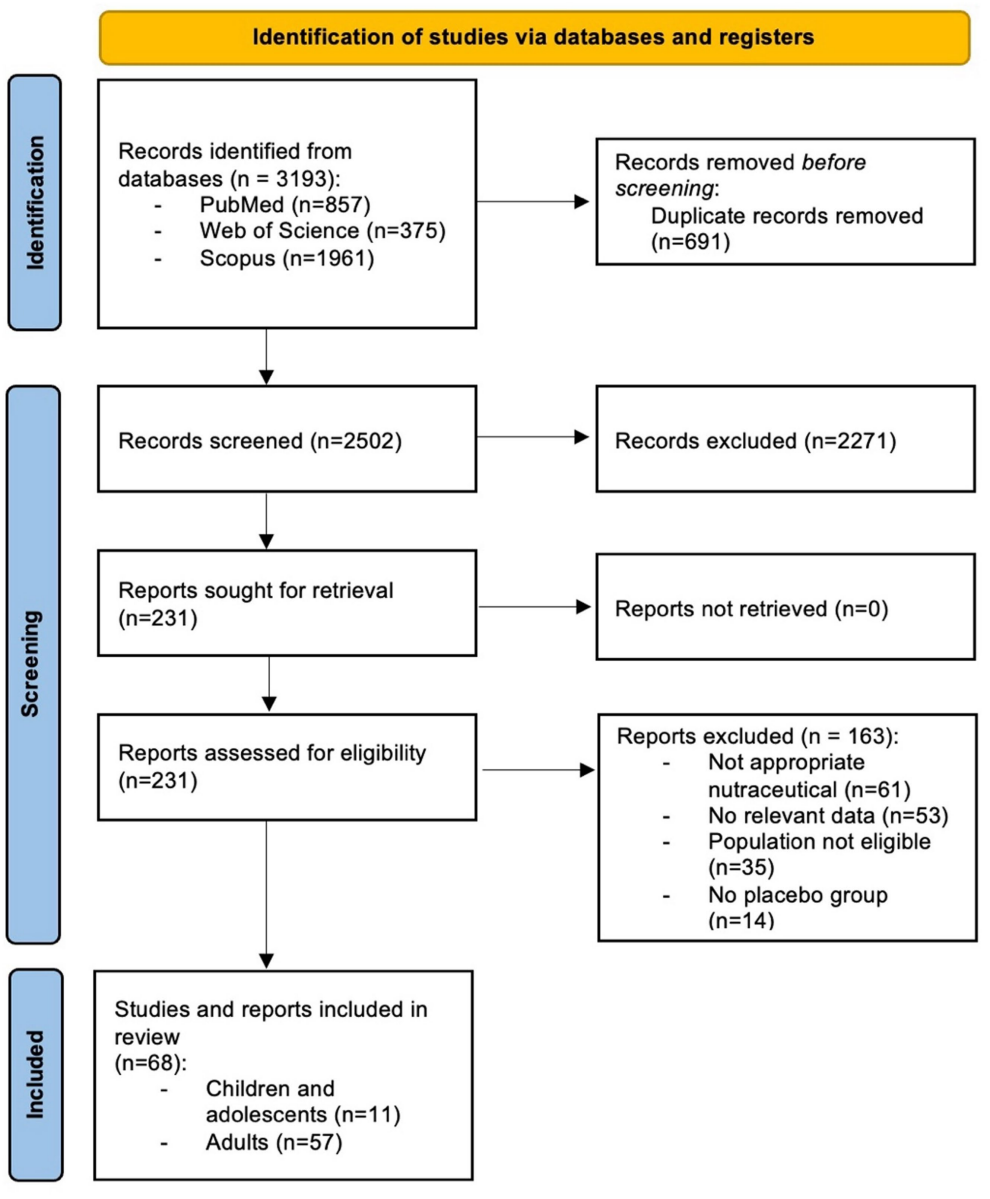
PRISMA flow diagram of the study selection process.

The study selection process is summarized in the PRISMA flow diagram (Figure 2). The initial search retrieved 3,193 records, of which 857 came from PubMed, 375 from Web of Science, and 1961 from Scopus. After removing 691 duplicates, 2,502 unique articles remained for title and abstract screening. At this stage, 2,271 studies were excluded because they did not meet the predefined eligibility criteria, leaving 231 articles for full-text evaluation.

Full-text screening resulted in the exclusion of 163 articles. The most frequent reasons for exclusion were that 61 did not investigate the appropriate nutraceuticals, 53 failed to report data relevant to the review outcomes, 35 involved populations outside the scope of the review and 14 did not include a placebo group. Ultimately, 68 randomized studies fulfilled all eligibility requirements and were included in the final analysis, of which 11 involved pediatric patients.

The descriptive characteristics of the studies are reported in Supplementary Table S3.

### 3.2 Risk of bias and assessment

Results of the risk-of-bias assessment for the included studies are presented visually using a summary bar plot across domains, Figure 3. Most studies had low risk of bias; issues were mainly in randomization and missing data, with few showing high risk. Moreover when evaluating the funnel plots, we found that in none of the outcome evaluated there was a small-study effects (*p*-value > 0.05, Supplementary Figures S1–S14).

**Figure 3:**
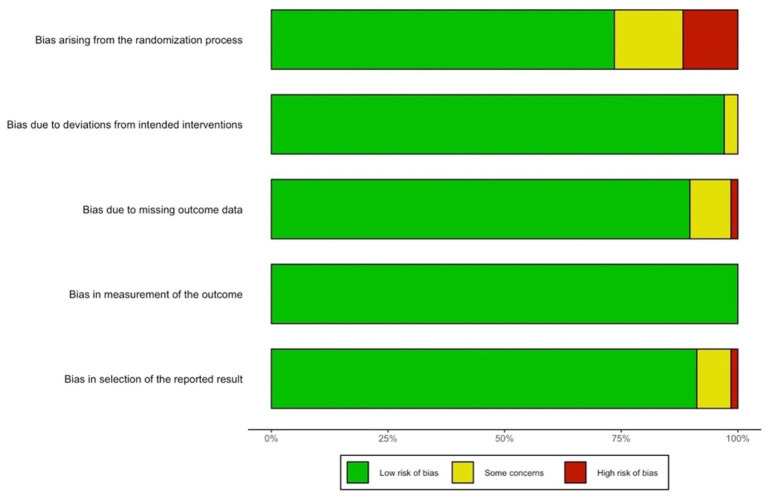
Risk of bias assessment across the five domains of the Cochrane Risk of Bias 2 tool. Most studies were judged at low risk of bias (green) in all domains. Some concerns (yellow) were identified mainly in the randomization process and in handling of missing outcome data, while a smaller proportion of studies showed high risk of bias (red), particularly in the randomization process.

### 3.3 Effects of different types of interventions on anthropometric characteristics

**Figure 4:**
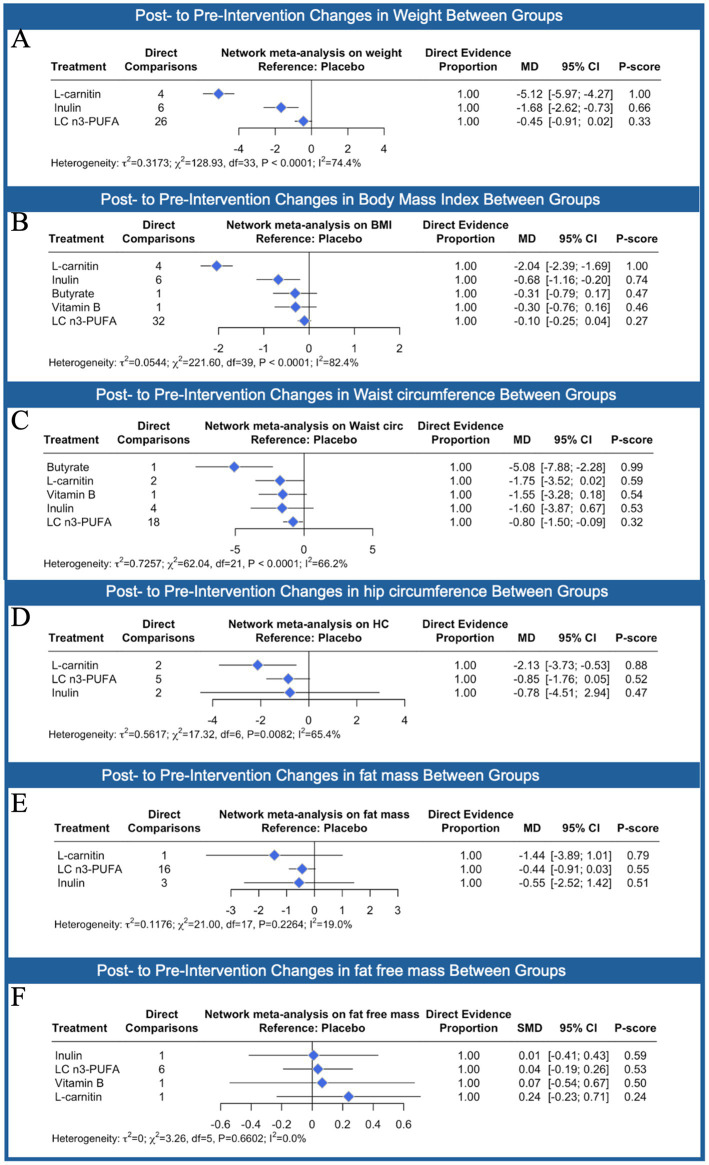
Network meta-analysis results comparing the effects of different nutraceutical interventions compared to placebo (reference) on body weight **(A)**, body mass index **(B)**, waist circumference **(C)**, hip circumference **(D)**, fat mass **(E)** and fat free mass **(F)**. The number of direct comparisons for each treatment group is listed, alongside the standardized mean difference (SMD), 95% confidence intervals (CI), and P-scores, which range from 0 (least effective) to 1 (most effective). Proportion values indicate the contribution of direct evidence to the network estimates. Heterogeneity statistics (*τ*^2^, *χ*^2^, *I*^2^), are reported below each graph to assess consistency across included studies.

Based on the results of the network meta-analysis (Figure 4), L-carnitine led to the most significant reduction in weight compared to placebo (MD: −5.12 kg; 95% CI: −5.97 to −4.27), followed by inulin (MD: −2.18 kg; 95% CI: −3.49 to −0.87). These changes were statistically significant, while LC n3-PUFA showed no meaningful difference from placebo (MD: −0.45 kg; 95% CI: −0.91 to 0.02).

A similar trend was observed for BMI, with L-carnitine yielding the greatest and statistically significant reduction (MD: −1.20 kg/m^2^; 95% CI: −1.67 to −0.74). In comparison, inulin (MD: −0.24 kg/m^2^; 95% CI: −1.06 to 0.57), butyrate (MD: −0.26 kg/m^2^; 95% CI: −1.25 to 0.73), vitamin B (MD: −0.20 kg/m^2^; 95% CI: −1.21 to 0.82), and LC n3-PUFA (MD: −0.18 kg/m^2^; 95% CI: −1.20 to 0.83) did not result in statistically significant changes when compared to placebo.

For waist circumference, butyrate showed the greatest reduction compared to placebo (MD: −5.08 cm; 95% CI: −7.58 to −2.21), followed by L-carnitine (MD: −4.30 cm; 95% CI: −6.28 to −2.27). In contrast, vitamin B (MD: −1.72 cm; 95% CI: −3.89 to 0.44), inulin (MD: −1.00 cm; 95% CI: −3.58 to 1.57), and LC n3-PUFA (MD: −0.88 cm; 95% CI: −3.44 to 1.68) did not lead to statistically significant changes. Overall, L-carnitine consistently ranked as the most effective intervention across all three anthropometric outcomes. Heterogeneity ranged from moderate to high across the analyses (*I*^2^: 42.4–74.4%). For hip circumference, none of the interventions showed statistically significant reductions compared to placebo. L-carnitine showed a small reduction (MD: −1.3 cm; 95% CI: −3.73 to 1.05), as did LC n3-PUFA (MD: −2.85 cm; 95% CI: −7.15 to 0.05), and inulin (MD: −0.78 cm; 95% CI: −4.51 to 2.94), but none reached statistical significance. In terms of fat mass, L-carnitine again showed the greatest reduction (MD: −1.44 kg; 95% CI: −3.98 to 1.11), though this result was not statistically significant. LC n3-PUFA (MD: −0.55 kg; 95% CI: −2.25 to 1.14) and inulin (MD: −0.5 kg; 95% CI: −2.52 to 1.42) also failed to show meaningful differences compared to placebo. For fat-free mass, all treatments produced very small and statistically non-significant effects. Inulin had a standardized mean difference (SMD) of 0.00 (95% CI: −0.43 to 0.44), LC n3-PUFA showed a slight negative effect (SMD: −0.04; 95% CI: −0.47 to 0.40), vitamin B had an SMD of −0.06 (95% CI: −0.51 to 0.38), and L-carnitine showed an SMD of −0.21 (95% CI: −0.72 to 0.31). Across these outcomes, none of the interventions produced statistically significant changes, and between-study heterogeneity ranged from low to moderate.

### 3.4 Effects of different types of interventions on blood pressure and C-reactive protein

For systolic blood pressure (SBP), none of the interventions showed statistically significant effects compared to placebo. Inulin had a slight reduction (SMD: −0.22; 95% CI: −1.17 to 0.73), while vitamin B (SMD: 0.20; 95% CI: −1.46 to 1.86) and LC n3-PUFA (SMD: −0.15; 95% CI: −0.61 to 0.30) also showed no significant changes. The heterogeneity was substantial across studies (*I*^2^ = 88.9%). For diastolic blood pressure (DBP), results were similarly inconclusive. Vitamin B showed a small, non-significant reduction (SMD: −0.52; 95% CI: −1.63 to 0.58), and inulin had a near-zero effect (SMD: −0.10; 95% CI: −1.20 to 1.01). LC n3-PUFA again showed no significant change (SMD: 0.03; 95% CI: −0.25 to 0.30). Heterogeneity was high for this outcome as well (*I*^2^ = 75.7%). For C-reactive protein (CRP), none of the interventions demonstrated significant effects compared with placebo. L-carnitine showed a modest, non-significant reduction (MD: −0.90; 95% CI: −3.12 to 1.32), inulin also indicated a slight reduction (MD: −0.43; 95% CI: −2.46 to 1.61), and LC n3-PUFA showed no effect (MD: 0.00; 95% CI: −0.18 to 0.18). Heterogeneity for this analysis was moderate (*I*^2^ = 82.4%). Forest-plot for the comparisons between groups is presented in Supplementary Figure S13. Although some interventions ranked relatively high according to P-scores, none demonstrated statistically significant effects compared with placebo for blood pressure or C-reactive protein. Rankings are based on point estimates and should therefore be interpreted in conjunction with the corresponding confidence intervals.

### 3.5 Effects of different types of interventions on glycemic profile

**Figure 5:**
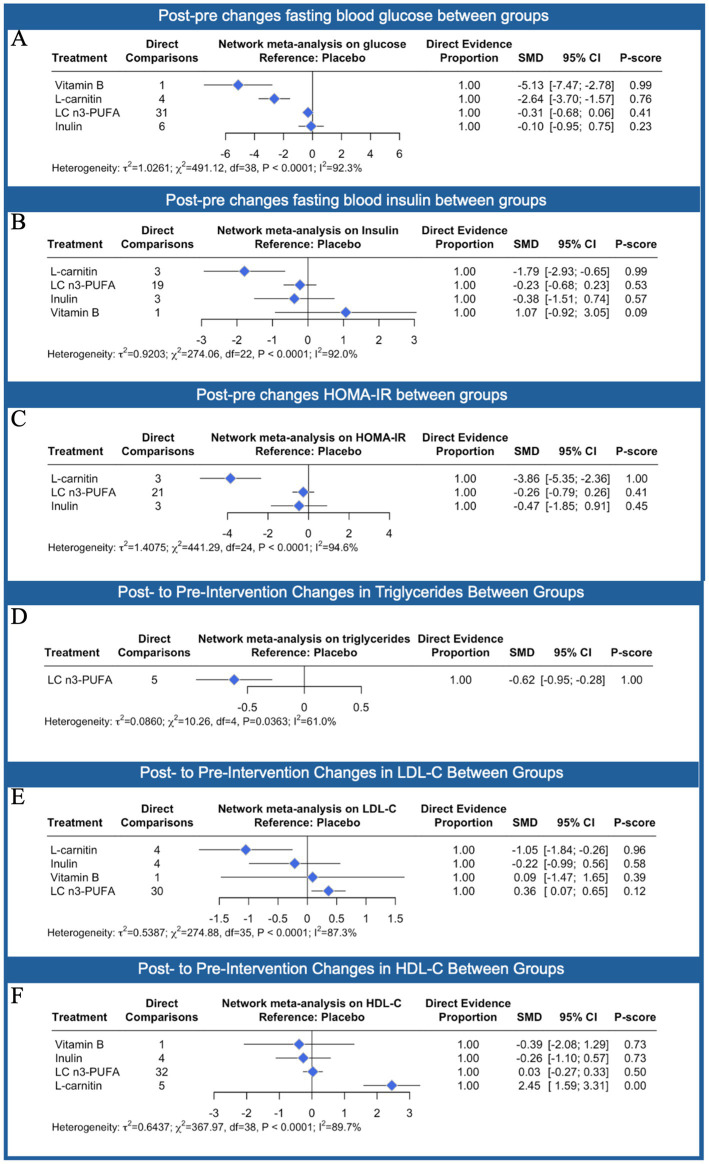
Network meta-analysis results comparing the effects of different nutraceutical interventions compared to placebo (reference) on fasting blood glucose **(A)**, fasting insulin **(B)**, homeostatic model assessment of insulin resistance (HOMA-IR; **C)**, triglycerides **(D)**, low-density lipoprotein LDL-C **(E)** and high-density lipoprotein, HDL-C **(F)**. The number of direct comparisons for each treatment group is listed, alongside the standardized mean difference (SMD), 95% confidence intervals (CI), and P-scores, which range from 0 (least effective) to 1 (most effective). Proportion values indicate the contribution of direct evidence to the network estimates. Heterogeneity statistics (*τ*^2^, *χ*^2^, *I*^2^) are reported below each graph to assess consistency across included studies.

Figure 5 presents the differences between the interventions on lipid and glycemic profiles referring to placebo. For fasting blood glucose, L-carnitine showed the most pronounced and statistically significant reduction compared to placebo (SMD: −5.13; 95% CI: −7.47 to −2.77). In contrast, vitamin B (SMD: −1.54; 95% CI: −3.74 to 0.67), LC n3-PUFA (SMD: −2.43; 95% CI: −7.05 to 2.18), and inulin (SMD: −0.16; 95% CI: −0.95 to 0.63) did not produce statistically significant effects. In terms of fasting insulin levels, none of the interventions led to significant changes. L-carnitine showed a small reduction (SMD: −1.79; 95% CI: −2.92 to −0.65), but with a P-score of 0.99, suggesting high ranking but moderate confidence. LC n3-PUFA (SMD: −0.70; 95% CI: −1.87 to 0.47), inulin (SMD: −0.21; 95% CI: −1.25 to 0.83), and vitamin B (SMD: 1.07; 95% CI: −0.92 to 3.05) also did not reach statistical significance. For HOMA-IR, which reflects insulin resistance, L-carnitine again showed the greatest reduction (SMD: −1.60; 95% CI: −2.85 to −0.35), indicating a potentially meaningful improvement. Neither LC n3-PUFA (SMD: −0.26; 95% CI: −1.28 to 0.76) nor inulin (SMD: −0.47; 95% CI: −1.85 to 0.91) showed significant effect. Overall, L-carnitine appeared to be the most effective intervention for improving glucose metabolism, with the strongest and most consistent reductions across glucose, insulin, and HOMA-IR. However, substantial heterogeneity was present in all outcomes (*I*^2^ range: 92.3–96.4%). For triglycerides, LC n3-PUFA was the only intervention assessed and showed a statistically significant reduction compared to placebo (SMD: −0.62; 95% CI: −0.95 to −0.28), suggesting a moderate lowering effect. In terms of LDL cholesterol (LDL-C), L-carnitine produced the most substantial decrease (SMD: −1.05; 95% CI: −1.84 to −0.26), indicating a statistically significant improvement. Inulin also showed a slight reduction (SMD: −0.22; 95% CI: −1.09 to 0.56), though this was not significant. In contrast, vitamin B had a negligible effect (SMD: 0.09; 95% CI: −1.47 to 1.65), and LC n3-PUFA was associated with a slight increase in LDL-C (SMD: 0.36; 95% CI: 0.07–0.65), which reached statistical significance. For HDL cholesterol (HDL-C), L-carnitine showed the strongest positive effect (SMD: 2.45; 95% CI: 1.59–3.31), indicating a significant increase in HDL levels. Other interventions, including vitamin B (SMD: −0.39; 95% CI: −2.08 to 1.29), inulin (SMD: −0.26; 95% CI: −1.10 to 0.57), and LC n3-PUFA (SMD: 0.03; 95% CI: −0.27 to 0.33), showed no meaningful changes. Overall, L-carnitine consistently improved both LDL and HDL profiles, while LC n3-PUFA effectively reduced triglycerides but was associated with a slight increase in LDL-C. Heterogeneity across lipid outcomes was moderate to high (*I*^2^ range: 61.0–89.7%).

### 3.6 Multivariate dose response analysis on anthropometric characteristics

**Figure 6:**
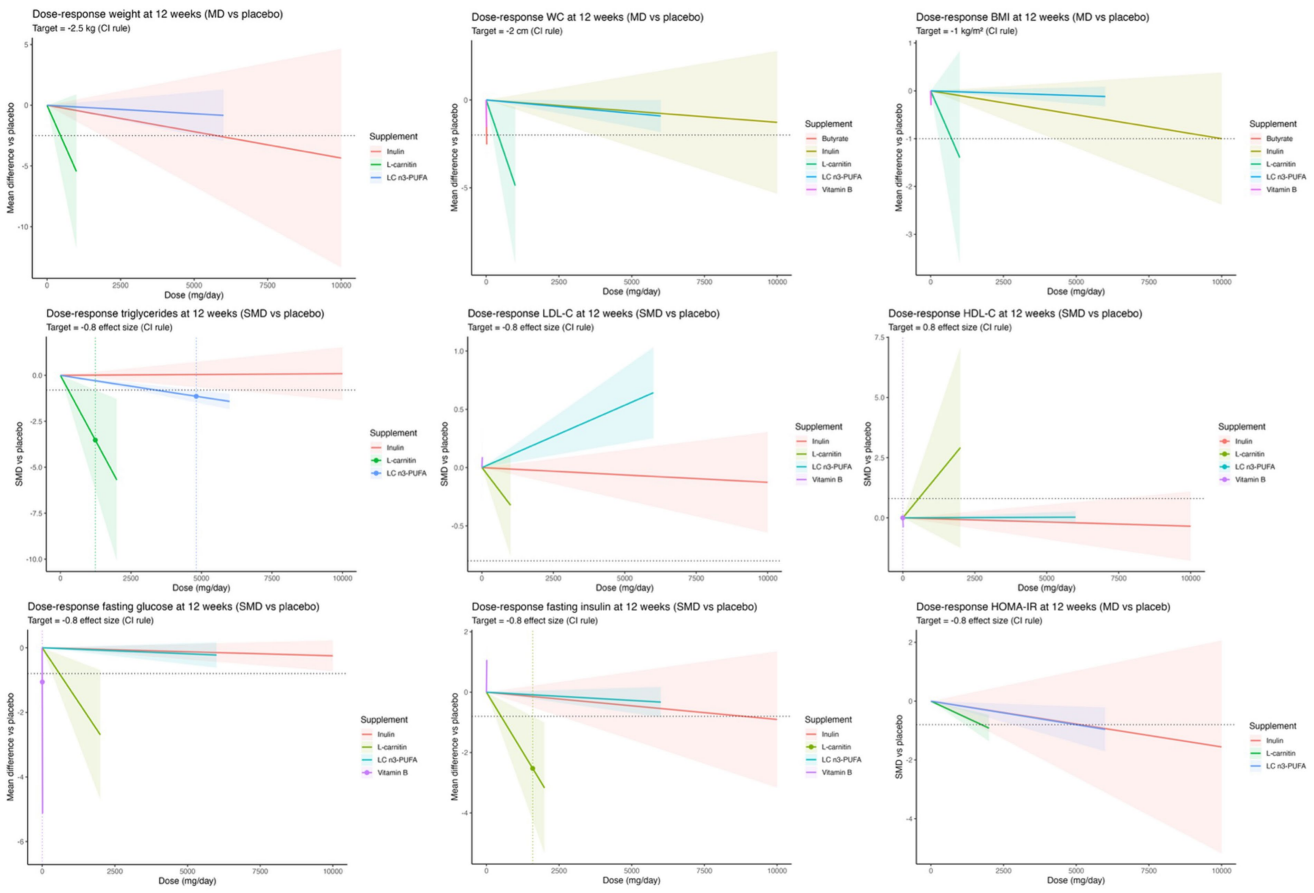
Multivariate dose–response network meta-analysis of supplementation effects on body composition (top), lipid profile (middle) and glycemic profile (bottom) after 12 weeks compared to placebo.

The multivariate dose–response network meta-analysis evaluated the relationship between supplement dosage and reductions in key anthropometric outcomes over a 12-week supplementation period (Figure 6).

For weight loss, the target was a 2.5 kg reduction compared to placebo. The estimated optimal daily doses required to reach this target were 458 mg for L-carnitine and 5,751 mg for inulin, while LC n3-PUFA failed to reach the desired target (−0.83 at 6,000 mg/day). Among these, L-carnitine was the most efficient, achieving the desired weight loss with the lowest dose and demonstrating the steepest and most effective dose–response curve.

For BMI reduction, targeting a 1.0 kg/m^2^ decrease, the optimal daily doses were 456 mg for L-carnitine, 10,000 mg for inulin, and 6,000 mg for LC n3-PUFA. Butyrate (20 mg/day * bodyweight) and vitamin B (14 mg/day) were also assessed. L-carnitine was the only intervention to meet the BMI target. All other supplements, including inulin and LC n3-PUFA, fell short of the 1.0 kg/m^2^ reduction, despite moderate or minor effects.

For waist circumference, the goal was a 2 cm reduction. The estimated effective doses were 410 mg for L-carnitine and 19.60 mg*bodyweight for butyrate. L-carnitine and butyrate each reached the target of a 2 cm reduction. In contrast, LC n3-PUFA (6,000 mg/day), inulin (10,000 mg/day) and vitamin B (14 mg/day) resulted in smaller reductions of 0.91 cm, 1.27 cm and 1.55 cm, respectively. However, it is important to note that none of these dose–response relationships reached statistical significance.

For EPA and DHA, multivariate dose–response analyses were performed using the best-fitting model for each anthropometric outcome. Across all outcomes, the EPA-only formulation consistently demonstrated steeper and more favorable dose–response curves compared to EPA: DHA combinations. Specifically, the EPA-only formulation outperformed the 1.78:1 and 1.85:1 EPA: DHA ratios for weight, BMI, and waist circumference. However, neither formulation achieved the predefined clinical thresholds (−2.5 kg weight loss, −1.0 kg/m^2^ BMI reduction, or −2.0 cm waist reduction) within the observed dose range (Supplementary Figure S14).

In terms of metabolic outcomes, for fasting blood glucose the target effect size of −0.8 was achieved by L-carnitine (504 mg/day) and vitamin B (3 mg/day), whereas inulin (−0.25 at 10,000 mg/day) and LC n3-PUFA (−0.22 at 6,000 mg/day) did not meet the threshold. For fasting insulin, the −0.8 target was reached by L-carnitine (504 mg/day) and by inulin (8,858 mg/day). LC n3-PUFA showed a reduction without reaching the target (−0.33, 6,000 mg/day), while vitamin B showed an opposite effect (+1.00 at 14 mg/day). For HOMA-IR, dose–response modeling suggested favorable reductions with all three evaluated supplements, with mean effective doses estimated at 1,741 mg/day for L-carnitine, 5,137 mg/day for inulin, and 4,988 mg/day for LC n3-PUFA. However, the predefined target was not reached within the observed dose ranges, and none of the associations achieved statistical significance, Figure 5.

For all three outcomes, DHA-only formulations consistently demonstrated steeper and more favorable dose–response curves compared to combined EPA: DHA formulations. Specifically, for fasting glucose, DHA-only outperformed the 1.38:1 EPA: DHA ratio; for fasting insulin, the DHA-only formulation showed stronger effects than the 1.71:1 ratio; and for HOMA-IR, EPA-only supplementation produced greater reductions compared to the 1.74:1 EPA: DHA ratio. Although none of the formulations reached the predefined target effect size of −0.8 within the observed dose range, DHA-only and EPA-only products showed greater potential for improving glucose homeostasis compared to mixed EPA: DHA combinations across all measures (Supplementary Figure S14).

For triglycerides, the predefined target effect size of −0.8 was achieved by L-carnitine (746 mg/day) and LC n3-PUFA (3,382 mg/day) and both reached statistical significance, whereas inulin (10,000 mg/day) showed no meaningful effect (0.20).

For LDL-C, none of the interventions reached the −0.8 threshold. L-carnitine (1,000 mg/day) and inulin (10,000 mg/day) produced small reductions of −0.32 and −0.12, respectively, while vitamin B (10,000 mg/day) showed a minimal increase (0.20), and LC n3-PUFA (6,000 mg/day) was associated with an increase of 0.64.

For HDL-C, the target effect size of +0.8 was achieved only by L-carnitine (552 mg/day; effect size: 0.81). In contrast, vitamin B (14 mg/day, −0.39), inulin (10,000 mg/day, −0.35), and LC n3-PUFA (6,000 mg/day, 0.03) did not reach the predefined threshold. Across all lipid outcomes, L-carnitine demonstrated the most consistent benefits, while inulin and vitamin B showed minimal or unfavorable effects, and LC n3-PUFA exhibited mixed results.

For C-reactive protein, none of the interventions reached the desired target (−0.8 effect size). Inulin (10,000 mg/day), L-carnitine (1,000 mg/day) and LC n3-PUFA (6,000 mg/day) reduced, respectively, by −0.06, −0.19 and −0.42.

For LDL-C, EPA-only supplementation showed a progressive and favorable reduction in SMD across the observed dose range, although the −0.8 effect size threshold was not reached. In contrast, HDL-C showed only a minimal increase with the 0.92:1 EPA: DHA formulation and failed to approach the predefined target of +0.8.

For triglycerides, both DHA-only and the 1.7:1 EPA: DHA formulation showed dose-dependent reductions, with DHA-only demonstrating a steeper and more effective slope, reaching the target effect size of −0.8 at higher doses.

For CRP, the strongest dose–response was observed with EPA-only supplementation, which far exceeded the −1.0 effect size threshold. While the 3:1 EPA: DHA formulation also reduced CRP levels, its slope was less pronounced than EPA-only (Supplementary Figure S14).

### 3.7 Subgroup analysis in children

#### 3.7.1 Subgroup analysis in children for anthropometric characteristics

A subgroup analysis was conducted in children to evaluate the effects of supplementation on anthropometric outcomes. For weight, LC n3-PUFA supplementation showed no significant effect compared with placebo (MD: −0.80; 95% CI: −4.42 to 2.81). In terms of BMI, butyrate was associated with a modest but statistically significant reduction (MD: −0.31; 95% CI: −0.52 to −0.10), while LC n3-PUFA showed no meaningful change (MD: 0.08; 95% CI: −0.03 to 0.18).

For waist circumference, butyrate supplementation led to a significant reduction (MD: −5.08 cm; 95% CI: −7.33 to −2.83), whereas LC n3-PUFA again showed no significant effect (MD: −0.58 cm; 95% CI: −4.39 to 3.23).

**Figure 7:**
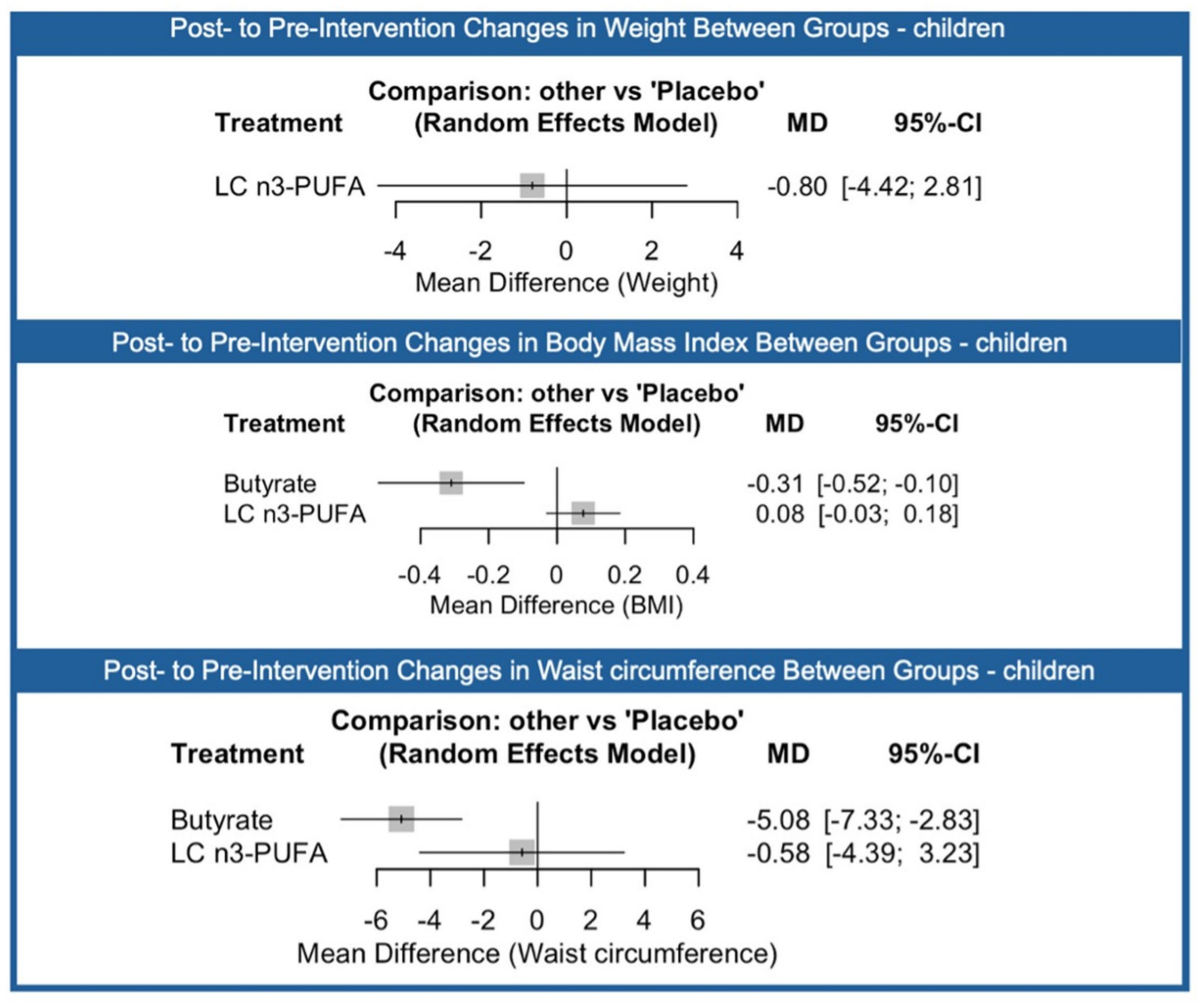
Network meta-analysis results comparing the effects of different nutraceutical interventions on children compared to placebo (reference) on (top) weight, (middle) body mass index (BMI) and (bottom) waist circumference. The number of direct comparisons for each treatment group is listed, alongside the standardized mean difference (SMD), 95% confidence intervals (CI), and P-scores, which range from 0 (least effective) to 1 (most effective). Proportion values indicate the contribution of direct evidence to the network estimates. Heterogeneity statistics (τ2, χ2, I2) are reported below each graph to assess consistency across included studies.

Overall, in this subgroup of children, butyrate supplementation demonstrated beneficial effects on BMI and waist circumference, while LC n3-PUFA showed no significant impact on weight, BMI, or waist circumference compared with placebo. The league table for the comparisons between groups it is presented in the Figure 7.

#### 3.7.2 Subgroup analysis in children for blood pressure

In the subgroup analysis of children, LC n3-PUFA supplementation did not show significant effects on blood pressure compared with placebo. For systolic blood pressure, the mean difference was −0.54 (95% CI: −1.75 to 0.67), indicating a no significant trend toward reduction. For diastolic blood pressure, the effect was negligible (MD: 0.01; 95% CI: −0.31 to 0.33). Overall, LC n3-PUFA supplementation in children did not significantly influence systolic or diastolic blood pressure (Supplementary Figure S15).

#### 3.7.3 Subgroup analysis in children for glycemic and lipid profile

**Figure 8:**
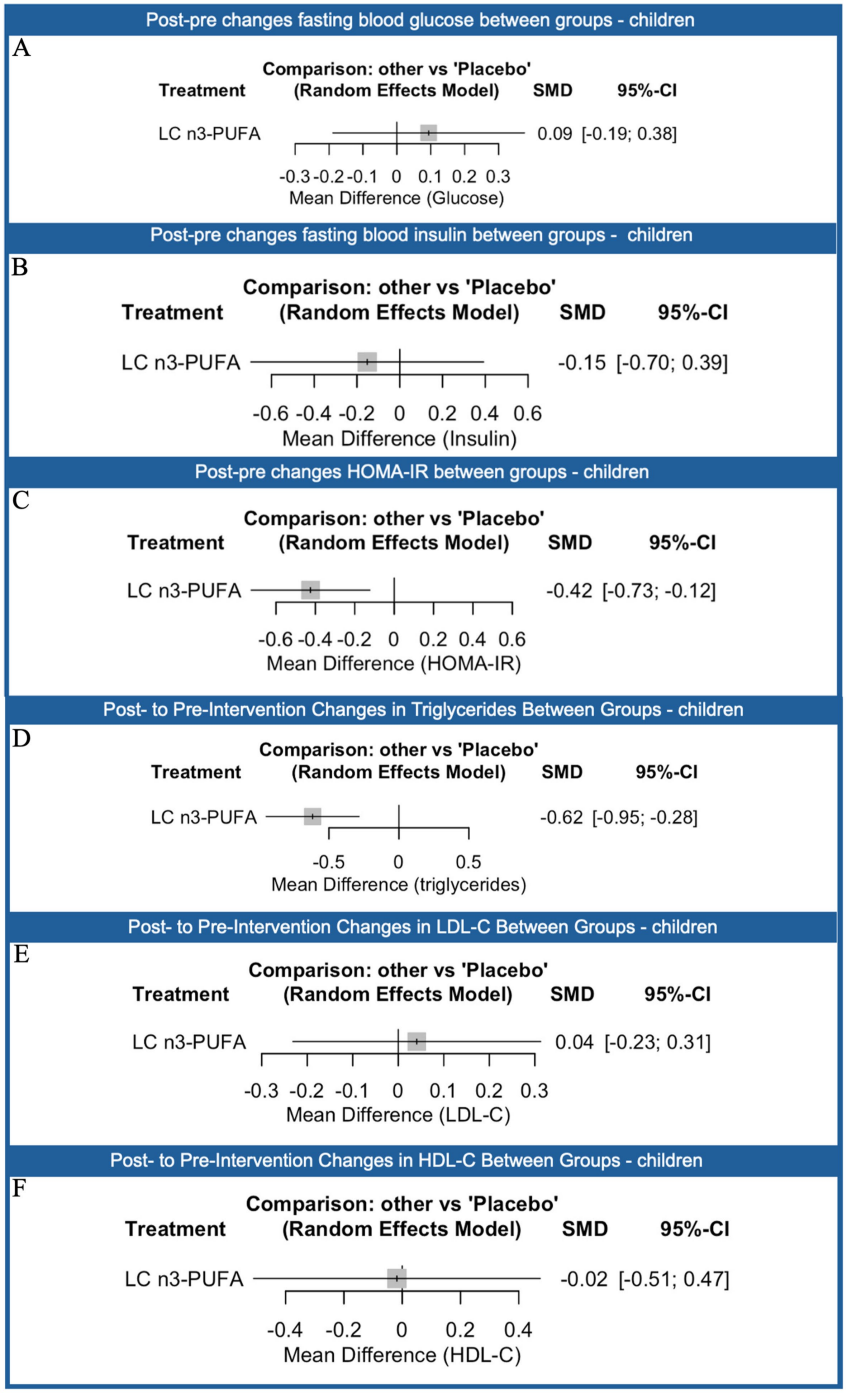
Network meta-analysis results comparing the effects of different nutraceutical interventions on children compared to placebo (reference) on fasting blood glucose **(A)**, fasting insulin **(B)**, homeostatic model assessment of insulin resistance (HOMA-IR, **C**), triglycerides **(D)**, low-density lipoprotein (LDL-C, **E**) and high-density lipoprotein (HDL-C, **F**). The number of direct comparisons for each treatment group is listed, alongside the standardized mean difference (SMD), 95% confidence intervals (CI), and P-scores, which range from 0 (least effective) to 1 (most effective). Proportion values indicate the contribution of direct evidence to the network estimates. Heterogeneity statistics (*τ*^2^, *χ*^2^, *I*^2^) are reported below each graph to assess consistency across included studies.

In the subgroup analysis of children, supplementation with LC n3-PUFA showed limited effects on glycemic outcomes (Figure 8). For fasting blood glucose, no significant change was observed compared with placebo (SMD: 0.09; 95% CI: −0.19 to 0.38). Similarly, fasting insulin levels were not significantly affected (SMD: −0.15; 95% CI: −0.70 to 0.39). In contrast, for HOMA-IR, LC n3-PUFA supplementation was associated with a modest but statistically significant reduction (SMD: −0.42; 95% CI: −0.73 to −0.12). In the subgroup analysis of children, LC n3-PUFA supplementation demonstrated mixed effects on lipid profiles compared with placebo. For triglycerides, LC n3-PUFA was associated with a statistically significant reduction (SMD: −0.62; 95% CI: −0.95 to −0.28). However, no significant effects were observed for LDL-C (SMD: 0.04; 95% CI: −0.23 to 0.31) or HDL-C (SMD: −0.02; 95% CI: −0.51 to 0.47).

## 4 Discussion

A healthy lifestyle, characterized by adherence to established dietary guidelines and an adequate level of physical activity, remains the cornerstone of effective obesity management. However, achieving and maintaining such habits is often challenging. For this reason, alongside pharmacological treatments and bariatric surgery, nutraceutical supplements have recently been introduced to the market. These products, through various mechanisms of action, appear to promote significant weight loss while avoiding major adverse effects. The effectiveness of these treatments on weight management and obesity-related cardiometabolic complications remains a topic of great interest, with growing evidence suggesting that specific nutraceuticals may provide valuable support by modulating key metabolic and inflammatory pathways. This network meta-analysis offered a comprehensive evaluation of the effects of different nutritional supplements, including inulin, butyrate, LC n3-PUFA (EPA DHA), vitamin B complexes and carnitine on anthropometric, metabolic, and lipid outcomes in the general population, with additional insights from pediatric subgroups.

### 4.1 Synthesis of the network meta-analysis

Overall, the interventions displayed heterogeneous efficacy, with some compounds producing strong and clinically relevant effects while others contributed only marginal or inconsistent benefits.

L-carnitine consistently emerged as the most effective intervention. Compared with placebo over a 12-week period, It significantly reduced body weight (MD: −5.12 kg), BMI (MD: −1.20 kg/m^2^), and waist circumference (MD: −4.30 cm). Moreover, it improved fasting glucose (SMD: −5.13), insulin resistance (HOMA-IR SMD: −1.60), and lipid profile, lowering LDL-C (SMD: −1.05) and increasing HDL-C (SMD: +2.45). These clinical benefits are supported by several potential mechanisms of action. First, L-carnitine facilitates the transport of long-chain fatty acids into mitochondria, where *β*-oxidation occurs, thereby increasing lipid utilization for energy and reducing fat accumulation (59–61). In addition, it can modulate key regulators of lipid metabolism and abiogenesis, such as carnitine palmitoyltransferase I, hormone-sensitive lipase, acyl-CoA oxidase, and PPAR-*γ*, contributing to a better balance between fat storage and oxidation (62–64). Another proposed mechanism involves the increase in acetyl-CoA, the final product of β-oxidation, which may influence glucose availability to the brain, indirectly affecting appetite control and energy expenditure (65, 66). Although not yet fully clarified, these mechanisms are consistent with our findings, which showed significant reductions in anthropometric measures and improvements in the glyco-lipid profile. Dose–response modeling confirmed that these benefits were obtained at relatively modest intakes: between 400 and 750 mg per day were sufficient to lower body weight, BMI, triglycerides, and fasting glucose; about 1,000 mg per day produced reductions in LDL cholesterol; and as little as 550 mg per day was effective in raising HDL cholesterol. The ability of L-carnitine to exert broad metabolic improvements at low doses underscores its potential as a versatile and clinically practical strategy for obesity management.

Long-chain omega-3 fatty acids (EPA/DHA) displayed a more selective profile. They significantly reduced triglycerides (SMD: −0.62) at ~3,382 mg/day. This outcome is biologically plausible, as omega-3 fatty acids are known to reduce hepatic triglyceride synthesis and enhance *β*-oxidation, partly through the activation of PPAR-*α* and modulation of transcription factors such as SREBP-1c (67, 68). They can also improve lipid clearance by upregulating lipoprotein lipase activity and influencing VLDL particle production. Consistent with their ability to suppress hepatic triglyceride synthesis and enhance β-oxidation. However, they had no meaningful effects on weight or BMI, and at higher intakes were associated with an increase in LDL-C (SMD: +0.36) possibly related to enhanced conversion of VLDL to LDL particles. Dose–response analyses highlighted that EPA-only and DHA-only formulations showed more favorable effects on glucose and triglycerides compared to EPA/DHA combinations, although predefined clinical targets were not reached. These results suggest omega-3 supplementation may be particularly useful in obese individuals with hypertriglyceridemia, though caution is warranted in patients with dyslipidemia.

In contrast, inulin demonstrated only modest and inconsistent effects. A daily dose of about 10 g produced a reduction of roughly 2 kg in body weight, yet no meaningful changes were observed in BMI, waist circumference, fasting glucose, or insulin resistance. Similarly, lipid outcomes showed negligible improvement, with no significant effects on triglycerides, LDL, or HDL cholesterol. These limited effects may be explained by proposed mechanisms of action: inulin can increase satiety by delaying gastric emptying, modulate gut microbiota composition, and enhance short-chain fatty acid production, which in turn might influence appetite regulation and energy balance (35, 46, 69). Dose–response analysis further indicated that inulin failed to reach the predefined target effect sizes even at the highest tested intakes. These findings suggest that inulin may contribute marginally to weight reduction, possibly via satiety regulation or gut microbiota modulation, but it lacks the potency required for meaningful clinical impact as a stand-alone intervention in obesity management.

The benefits of butyrate have been reported only in a study conducted on pediatric populations, where supplementation was associated with improvements in BMI (MD: −0.31) and waist circumference (MD: −5.08 cm) at dose of 20 mg/kg.

The biological plausibility of butyrate’s action stems from its role as a short-chain fatty acid produced by microbial fermentation of dietary fibers. Butyrate provides energy to colonocytes, strengthens intestinal barrier integrity, and modulates immune and metabolic pathways through G-protein–coupled receptor activation and histone deacetylase inhibition (70, 71). These mechanisms could, in principle, influence lipid metabolism, insulin sensitivity, and energy balance. Although preliminary findings highlight a promising area for further investigation, current evidence remains insufficient to recommend butyrate as a routine intervention for obesity management.

Vitamin B supplementation, finally, did not produce significant or consistent improvements across anthropometric, metabolic, or lipid parameters. Dose–response analysis indicated that daily intakes of around 14 mg were insufficient to affect BMI, glucose, or lipid outcomes. In some models, vitamin B supplementation was even associated with a small increase in fasting insulin, raising concerns about possible unfavorable metabolic effects.

With respect to cardiovascular and inflammatory markers, none of the supplements, including L-carnitine and omega-3 fatty acids, significantly improved blood pressure or CRP levels. This indicates that the primary benefits of nutraceuticals are confined to weight, glycemic control, and lipid metabolism, rather than broader cardiometabolic or inflammatory endpoints. In this context, higher P-scores observed for some interventions should not be interpreted as evidence of efficacy, as rankings reflect relative point estimates rather than statistical significance and must be considered alongside confidence intervals.

In Table 2, the effective dose ranges and main metabolic effects of selected dietary supplements are summarized.

**Table 2:** Supplement dose ranges and effects.

Supplement	Age group	Dosage range (mg/day)	Effects
L-carnitine	Adults	250–2,000	400–750 mg/day: significant ↓ weight (−5.1 kg), ↓ BMI (−1.2 kg/m^2^), ↓ waist circumference (−4.3 cm), ↓ TG, ↓ glucose 550 mg/day: ↑ HDL-C Strongest glycemic improvement 1,000 mg/day: ↓ LDL-C
	Children	–	No pediatric studies
	Total	250–2,000	Most effective intervention across anthropometric, metabolic, and lipid outcomes
LC n3-PUFA (total)	Adults	250–6,000	↓ Triglycerides at ~3,400 mg/day (SMD: −0.62) ↑ LDL-C at 6,000 mg/day (SMD: +0.36) No significant effect on weight/BMI Small, non-significant changes at 6,000 mg/day
	Children	250–3,000	↓ Triglycerides (SMD: −0.62) No effect on LDL-C or HDL-C No significant impact on weight, BMI, or waist circumference Modest ↓ HOMA-IR (SMD: −0.42)
	Total	250–6,000	Consistent TG lowering Risk of LDL-C increase at higher doses
EPA	Adults	90–3,000	EPA-only produced steeper reductions in LDL-C Outperformed EPA: DHA for weight/BMI/waist, but targets not achieved
	Children	90–2,000	Limited data; not superior to DHA-only
	Total	90–3,000	More favorable metabolic profile than EPA: DHA blends
DHA	Adults	41.4–3,860	DHA-only showed stronger effects on fasting glucose and TG (target TG reduction achieved at higher doses)
	Children	210–1,000	Limited data; no consistent effects on weight/BMI
	Total	41.4–3,860	Steeper TG-lowering and glycemic benefits compared with EPA: DHA
Inulin	Adults	200–21,000	~10,000 mg/day: modest ↓ weight (−2.2 kg) no significant effects on BMI, waist, glucose, insulin, TG, LDL-C, HDL-C
	Children	–	No pediatric studies
	Total	200–21,000	Limited efficacy
Butyrate	Adults	–	No adult studies
	Children	20 mg/kg bow	↓ BMI (−0.31), ↓ waist circumference (−5.08 cm) No significant effects on weight or BP
	Total	Pediatric only	Promising in children; evidence still preliminary
Vitamin B complex	Adults	40 (≈14 mg active)	No significant effects did not reach efficacy thresholds
	Children	–	No pediatric studies
	Total	40	Minimal or unfavorable effects

### 4.2 Limitations

There are several important limitations that should be considered when interpreting the findings. First, a high degree of heterogeneity was observed across the included trials, reflecting differences in study populations, baseline characteristics, and outcome measures. Such variability reduces the comparability of results and may partly explain the inconsistency of some estimates. Second, most of the available studies investigated supplementation over relatively short intervention periods, averaging approximately twelve weeks. This restricted timeframe limits the ability to evaluate long-term efficacy, safety, and sustainability, which are crucial for interventions targeting chronic conditions such as obesity and metabolic disorders.

Third, there was substantial variability in the dosages used across studies, complicating the interpretation of dose–response relationships. Although dose–response analyses were conducted, the wide range of doses and the absence of standardized protocols hinder robust conclusions. Fourth, methodological limitations in the included trials, such as small sample sizes, inadequate blinding, and inconsistent reporting of adherence or adverse events, may have introduced bias and affected the overall strength of evidence.

Finally, evidence in pediatric populations was limited and derived from a small number of studies and concerned only a few nutraceuticals. This makes it difficult to generalize the results to younger age groups, despite the potential relevance of early-life interventions for preventing long-term cardiometabolic risk.

### 4.3 Implications for research, practice, and policy

The findings of this network meta-analysis carry important implications for future research, clinical practice, and health policy. From a research perspective, longer trials are urgently needed to determine the long-term safety, durability, and scalability of nutritional supplementation strategies, particularly in pediatric populations. Standardization of dosages and protocols would help clarify dose–response relationships and reduce heterogeneity across studies. Moreover, mechanistic investigations are warranted to better elucidate the age-specific benefits of butyrate, the paradoxical LDL-raising effect of LC n3-PUFA, and the reasons why some nutraceuticals exert metabolic benefits but fail to consistently modulate CRP or blood pressure. Future studies should also prioritize hard clinical outcomes, such as the incidence of type 2 diabetes and cardiovascular events, in addition to surrogate metabolic markers. Some outcomes, particularly systolic and diastolic blood pressure, showed very high heterogeneity (*I*^2^ > 75%), likely due to differences in study populations, intervention characteristics and supplementation duration. Because of the limited number of studies per comparison and the variability of study-level characteristics, we were unable to conduct sensitivity analyses or meta-regressions to explore sources of heterogeneity. Therefore, these results should be interpreted with caution. Given the number of comparisons performed across outcomes and dose–response analyses, no formal adjustment for multiple testing was applied; therefore results with marginal statistical significance should be interpreted cautiously.

In terms of clinical practice, the results suggest that L-carnitine represents the most versatile and effective option, with consistent benefits across weight management, glucose metabolism, and lipid regulation, achievable at relatively modest doses in adults; however, there is no evidence available in the pediatric population. LC n3-PUFA retain an important role in patients with obesity and hypertriglyceridemia, although their use requires caution in those with elevated LDL cholesterol. In contrast, inulin appears to provide only ancillary benefits, while butyrate shows promise in children but remains premature for routine clinical application. Vitamin B supplementation, on the other hand, does not currently have evidence to support its use in obesity management. Taken together, these findings encourage a more personalized approach, where supplementation is guided by individual metabolic profiles and comorbidities rather than applied uniformly.

At the policy level, the results highlight the need to integrate nutritional supplements into evidence-based guidelines only when their efficacy and safety are clearly supported by robust data. Investments in independent, high-quality research are crucial to mitigate conflicts of interest and strengthen the evidence base. Policymakers should also support the development of sustainable and tailored interventions within healthcare systems, moving away from one-size-fits-all strategies. Finally, special consideration should be given to vulnerable groups, such as children and individuals with metabolic disorders, ensuring that recommendations are both effective and equitable.

## 5 Conclusion

This network meta-analysis confirms that nutritional supplements exert heterogeneous effects on obesity-related outcomes. L-carnitine emerged as the most versatile intervention, while LC n3-PUFA, inulin, butyrate, and vitamin B showed more selective or limited benefits. Importantly, preliminary findings suggest that age may modulate the response to supplementation, with butyrate and LC n3-PUFA showing potential in pediatric populations. However, evidence in children remains less extensively reported in the literature, underlining the need for further studies to clarify age-specific effects and to develop safe, effective, and tailored strategies for obesity management across the lifespan.
