# Supplementary material for: Nutraceuticals for the management of weight and inflammation-related complications in obesity: a pediatric perspective. Systematic review and network meta-analysis
Source: Front Nutr. 2026 Feb 12;13:1715574. doi: 10.3389/fnut.2026.1715574 (PMC12937135; doi:10.3389/fnut.2026.1715574)
Supplement: Supplementary file 1 [file Table_1.docx]

**Nutraceuticals for the management of weight and inflammation-related complications in obesity: a pediatric perspective. Systematic review and network meta-analysis**

**Supplementary materials**

Index

[Figure S1. Funnel plot of network meta-analysis depicting the relationship between effect size versus standard error for the effect of different therapies on weight. 5](#_Toc217911439)

[Figure S2. Funnel plot of network meta-analysis depicting the relationship between effect size versus standard error for the effect of different therapies on body mass index (BMI). 6](#_Toc217911440)

[Figure S3. Funnel plot of network meta-analysis depicting the relationship between effect size versus standard error for the effect of different therapies on waist circumference. 7](#_Toc217911441)

[Figure S4. Funnel plot of network meta-analysis depicting the relationship between effect size versus standard error for the effect of different therapies on hip circumference 8](#_Toc217911442)

[Figure S5. Funnel plot of network meta-analysis depicting the relationship between effect size versus standard error for the effect of different therapies on fat mass. 9](#_Toc217911443)

[Figure S6. Funnel plot of network meta-analysis depicting the relationship between effect size versus standard error for the effect of different therapies on fat free mass. 10](#_Toc217911444)

[Figure S7. Funnel plot of network meta-analysis depicting the relationship between effect size versus standard error for the effect of different therapies on fasting glucose. 11](#_Toc217911445)

[Figure S8. Funnel plot of network meta-analysis depicting the relationship between effect size versus standard error for the effect of different therapies on fasting insulin. 12](#_Toc217911446)

[Figure S9. Funnel plot of network meta-analysis depicting the relationship between effect size versus standard error for the effect of different therapies on homeostatic model assessment of insulin resistance (HOMA-IR). 13](#_Toc217911447)

[Figure S10. Funnel plot of network meta-analysis depicting the relationship between effect size versus standard error for the effect of different therapies on triglycerides. 14](#_Toc217911448)

[Figure S11. Funnel plot of network meta-analysis depicting the relationship between effect size versus standard error for the effect of different therapies on low-density lipoprotein (LDL-C). 15](#_Toc217911449)

[Figure S12. Funnel plot of network meta-analysis depicting the relationship between effect size versus standard error for the effect of different therapies on high-density lipoprotein (HDL-C). 16](#_Toc217911450)

[Figure S13. Network meta-analysis results comparing the effects of different nutraceutical interventions compared to placebo (reference) on (top) systolic blood pressure, and (bottom) diastolic blood pressure. The number of direct comparisons for each treatment group is listed, alongside the standardized mean difference (SMD), 95% confidence intervals (CI), and P-scores, which range from 0 (least effective) to 1 (most effective). Proportion values indicate the contribution of direct evidence to the network estimates. Heterogeneity statistics (τ², χ², I²) are reported below each graph to assess consistency across included studies. 17](#_Toc217911451)

[Figure S14. Multivariate dose-response network meta-analysis of DHA and EPA supplementation effects on body composition (top), lipid profile (middle) and glycemic profile (bottom) after 12 weeks compared to placebo. 19](#_Toc217911452)

[Figure S14. Network diagrams illustrating the available comparisons for anthropometric and metabolic outcomes. Line thickness reflects the number of studies directly comparing each intervention pair, while node size corresponds to the total number of participants in each treatment group. Each node indicates a treatment group: placebo or the type of supplementation. 21](#_Toc217911453)

[Figure S15. Network meta-analysis results comparing the effects of different nutraceutical interventions on children compared to placebo (reference) on (top) systolic blood pressure, and (bottom) diastolic blood pressure. The number of direct comparisons for each treatment group is listed, alongside the standardized mean difference (SMD), 95% confidence intervals (CI), and P-scores, which range from 0 (least effective) to 1 (most effective). Proportion values indicate the contribution of direct evidence to the network estimates. Heterogeneity statistics (τ², χ², I²) are reported below each graph to assess consistency across included studies. 22](#_Toc217911454)

[Figure S16. Node-splitting analysis for body weight (placebo-controlled comparisons). 23](#_Toc217911455)

[Figure S17. Node-splitting analysis for body mass index (BMI, placebo-controlled comparisons). 24](#_Toc217911456)

[Figure S18. Node-splitting analysis for waist circumference (placebo-controlled comparisons). 25](#_Toc217911457)

[Figure S19. Node-splitting analysis for hip circumference (placebo-controlled comparisons). 26](#_Toc217911458)

[Figure S20. Node-splitting analysis for fat mass (placebo-controlled comparisons). 27](#_Toc217911459)

[Figure S21. Node-splitting analysis for fat free mass (placebo-controlled comparisons). 28](#_Toc217911460)

[Figure S22. Node-splitting analysis for fasting glucose (placebo-controlled comparisons). 29](#_Toc217911461)

[Figure S23. Node-splitting analysis for fasting insulin (placebo-controlled comparisons). 30](#_Toc217911462)

[Figure S23. Node-splitting analysis for homeostatic model assessment of insulin resistance (HOMA-IR, placebo-controlled comparisons). 31](#_Toc217911463)

[Figure S23. Node-splitting analysis for triglycerides (placebo-controlled comparisons). 32](#_Toc217911464)

[Figure S24. Node-splitting analysis for low-density lipoprotein (LDL-C, placebo-controlled comparisons). 33](#_Toc217911465)

[Figure S25. Node-splitting analysis for high-density lipoprotein (HDL-C, placebo-controlled comparisons). 34](#_Toc217911466)

[Table S1. PRISMA NMA Checklist of Items to Include When Reporting A Systematic Review Involving a Network Meta-analysis 35](#_Toc217911467)

[Table S2. Search strategy employed for Pubmed/medline database. 39](#_Toc217911468)

[Table S3. Descriptive characteristics of the studies included. 40](#_Toc217911469)

[Table S4. Prediction intervals for the comparisons between nutraceuticals interventions with the placebo interventions. 52](#_Toc217911470)


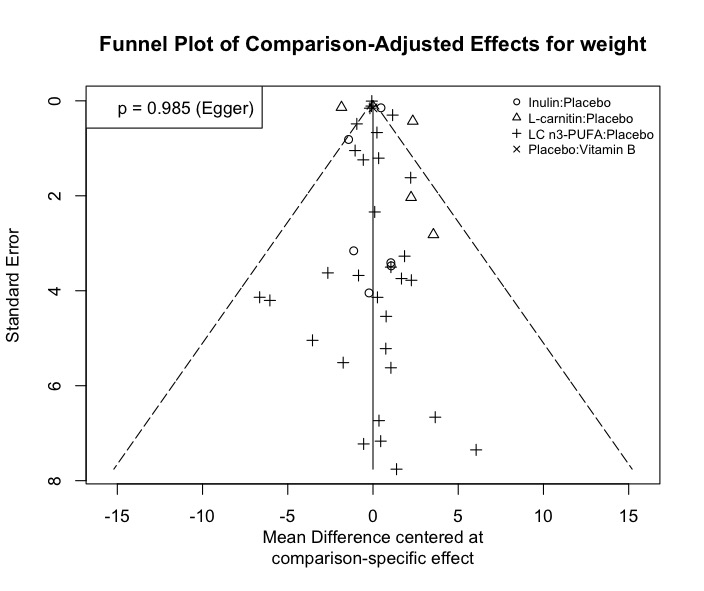


# **Figure S1**. Funnel plot of network meta-analysis depicting the relationship between effect size versus standard error for the effect of different therapies on weight.


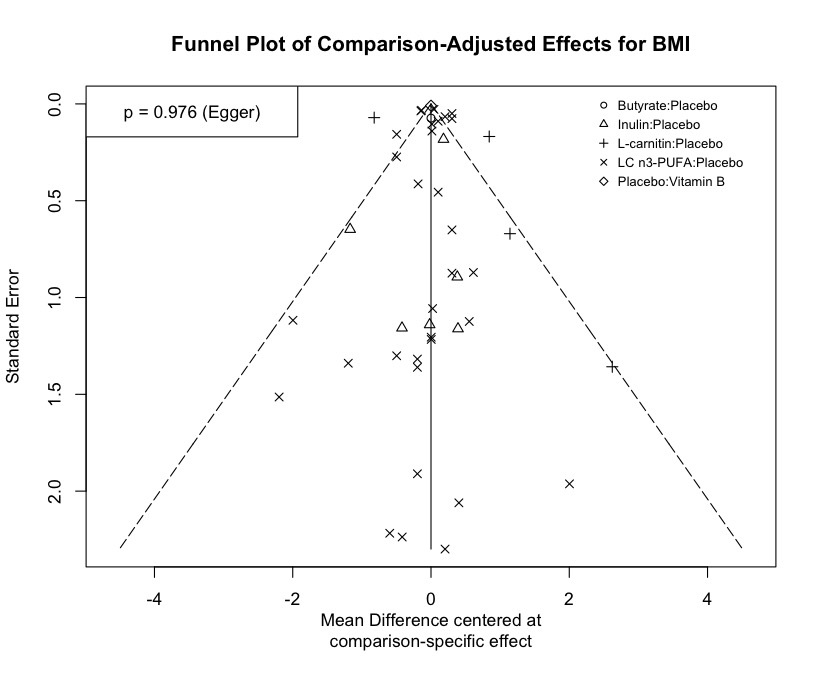


# **Figure S2**. Funnel plot of network meta-analysis depicting the relationship between effect size versus standard error for the effect of different therapies on body mass index (BMI).


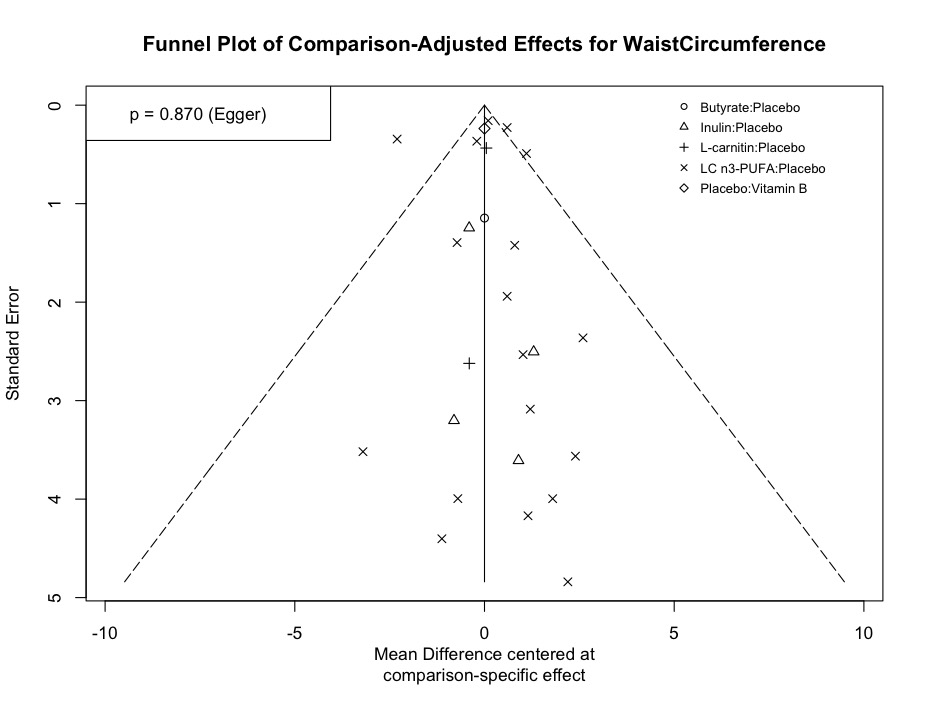


# **Figure S3**. Funnel plot of network meta-analysis depicting the relationship between effect size versus standard error for the effect of different therapies on waist circumference.


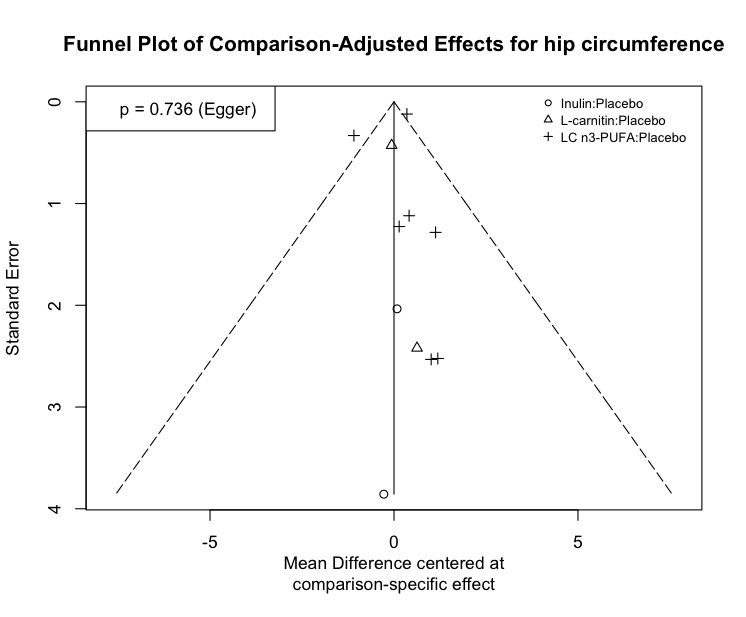


**Figure S4**. Funnel plot of network meta-analysis depicting the relationship between effect size versus standard error for the effect of different therapies on hip circumference.


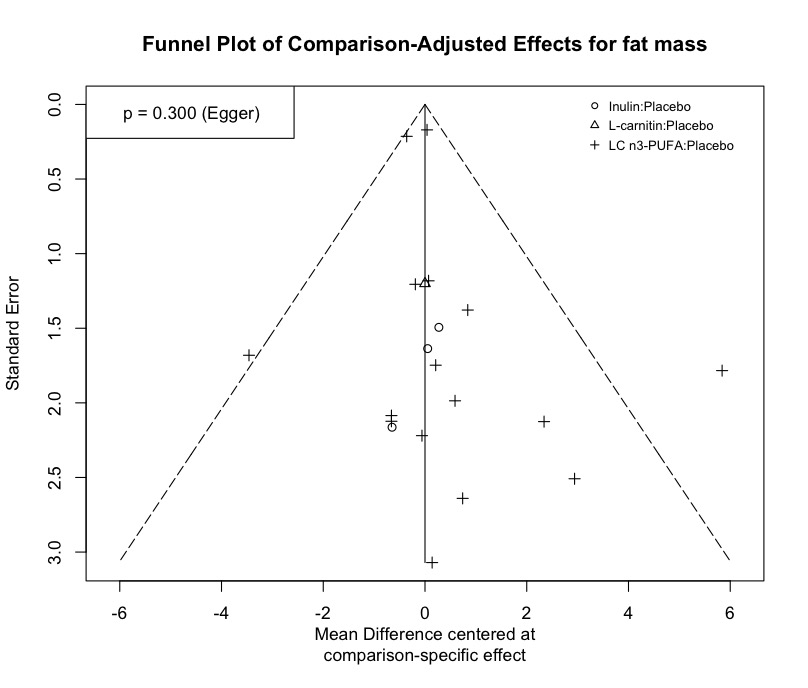


# **Figure S5**. Funnel plot of network meta-analysis depicting the relationship between effect size versus standard error for the effect of different therapies on fat mass.


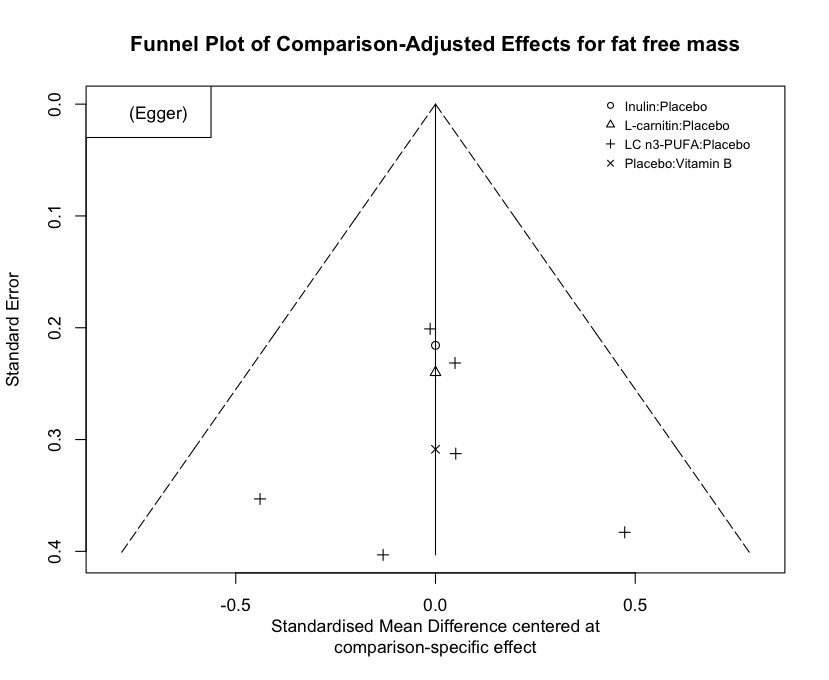


# **Figure S6**. Funnel plot of network meta-analysis depicting the relationship between effect size versus standard error for the effect of different therapies on fat free mass.


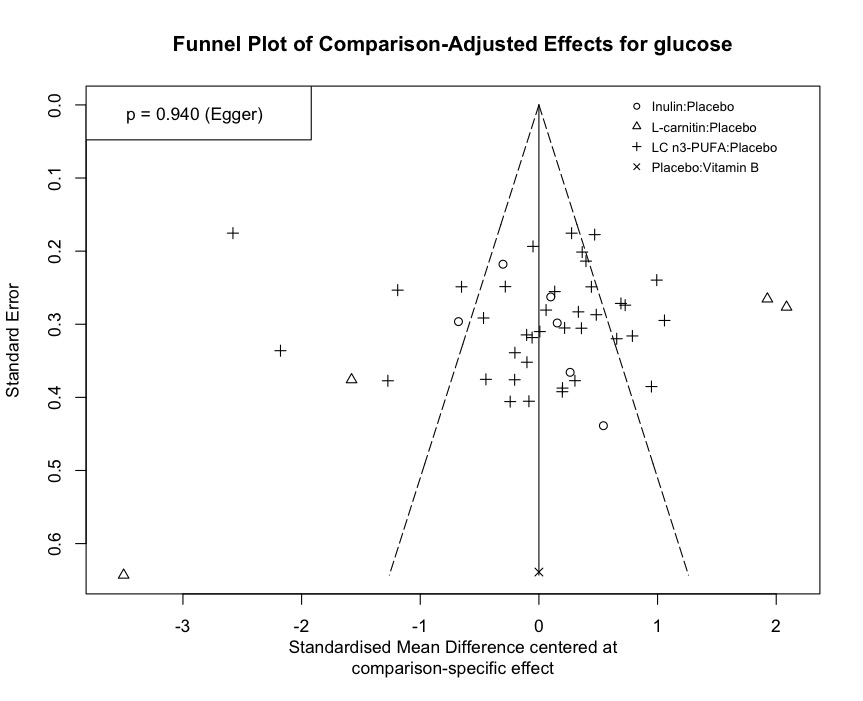


# **Figure S7**. Funnel plot of network meta-analysis depicting the relationship between effect size versus standard error for the effect of different therapies on fasting glucose.


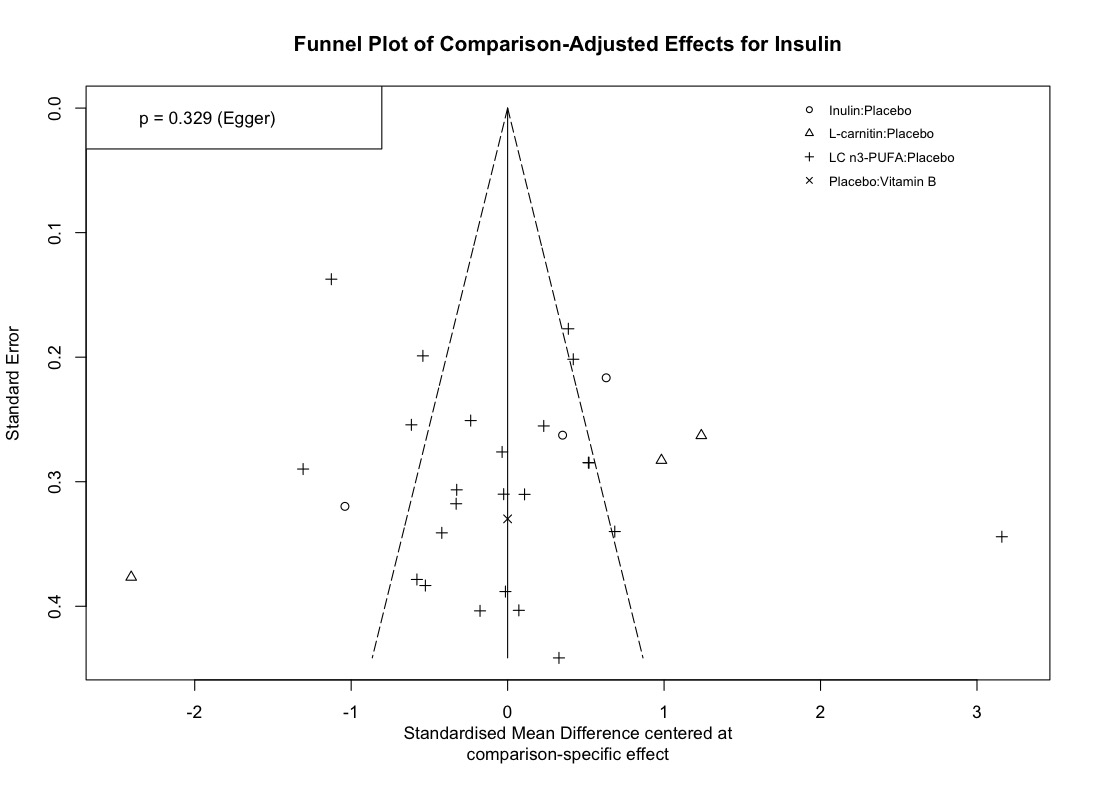


# **Figure S8**. Funnel plot of network meta-analysis depicting the relationship between effect size versus standard error for the effect of different therapies on fasting insulin.


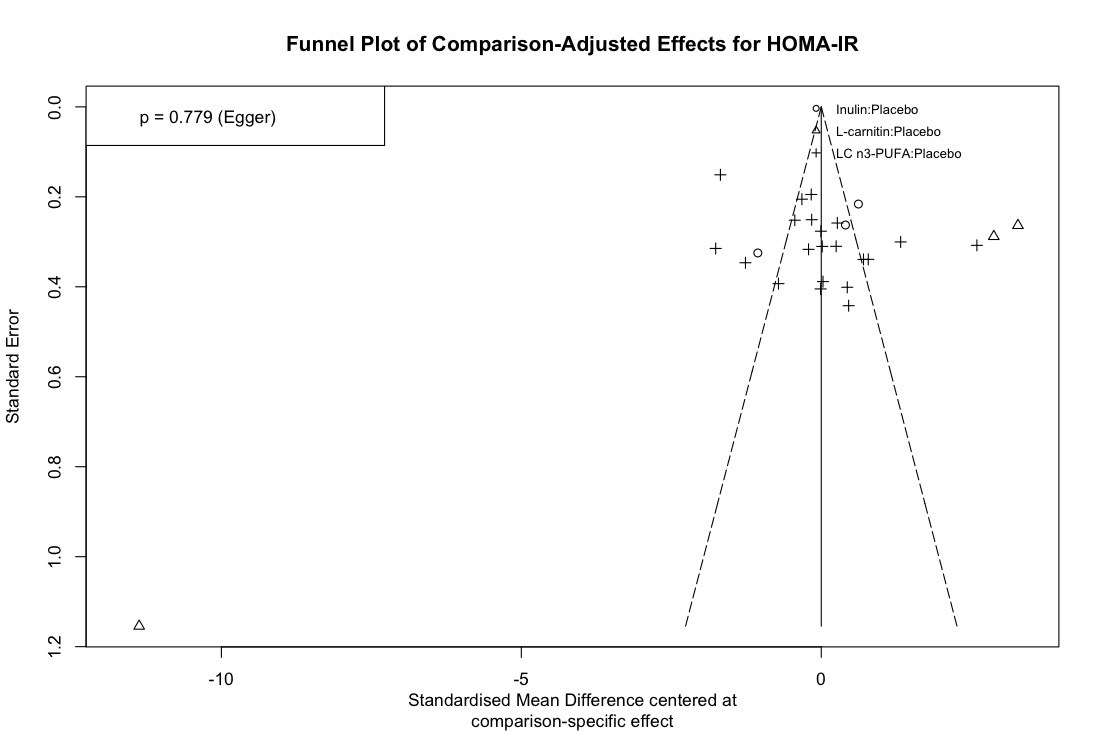


# **Figure S9**. Funnel plot of network meta-analysis depicting the relationship between effect size versus standard error for the effect of different therapies on homeostatic model assessment of insulin resistance (HOMA-IR).


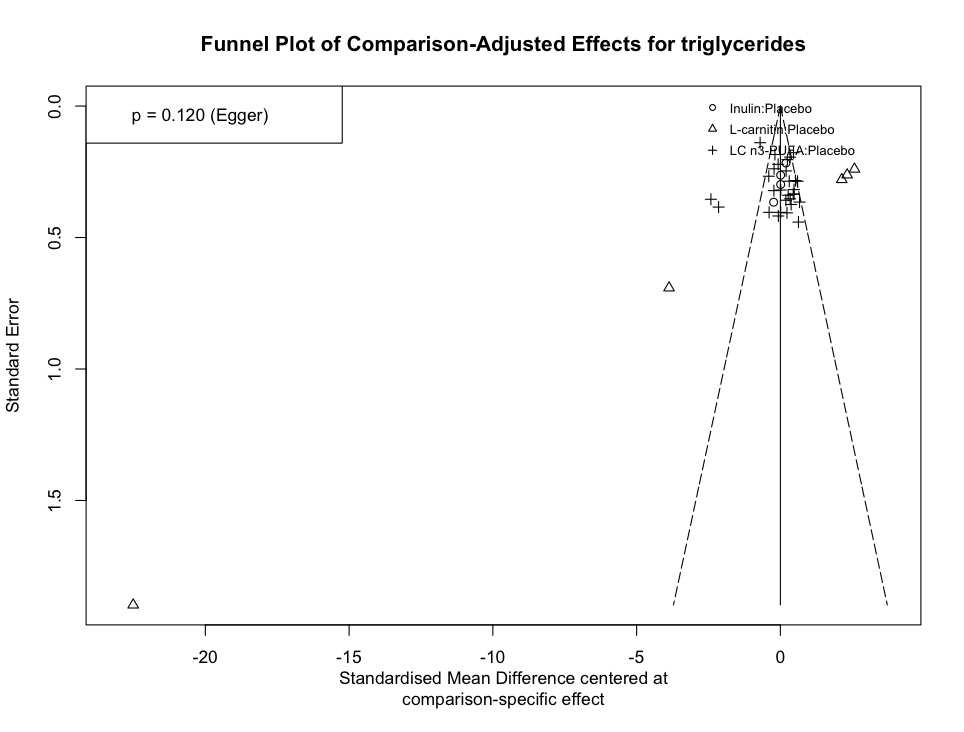


# **Figure S10**. Funnel plot of network meta-analysis depicting the relationship between effect size versus standard error for the effect of different therapies on triglycerides.


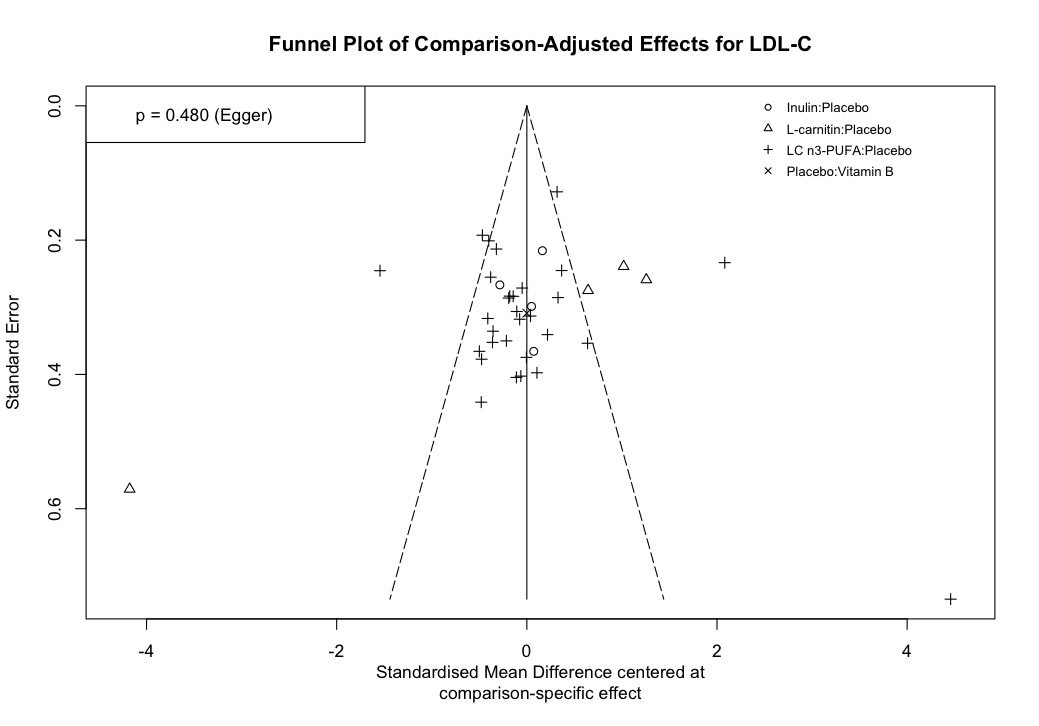


# **Figure S11**. Funnel plot of network meta-analysis depicting the relationship between effect size versus standard error for the effect of different therapies on low-density lipoprotein (LDL-C).


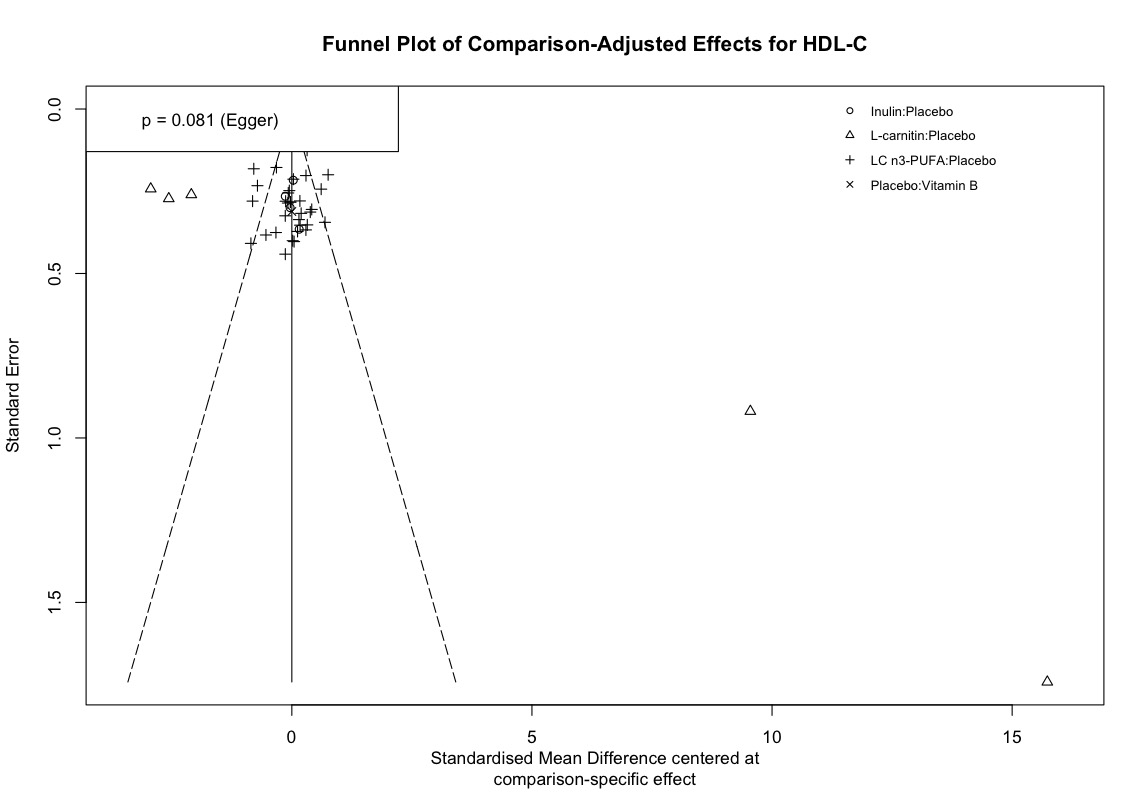


# **Figure S12**. Funnel plot of network meta-analysis depicting the relationship between effect size versus standard error for the effect of different therapies on high-density lipoprotein (HDL-C).


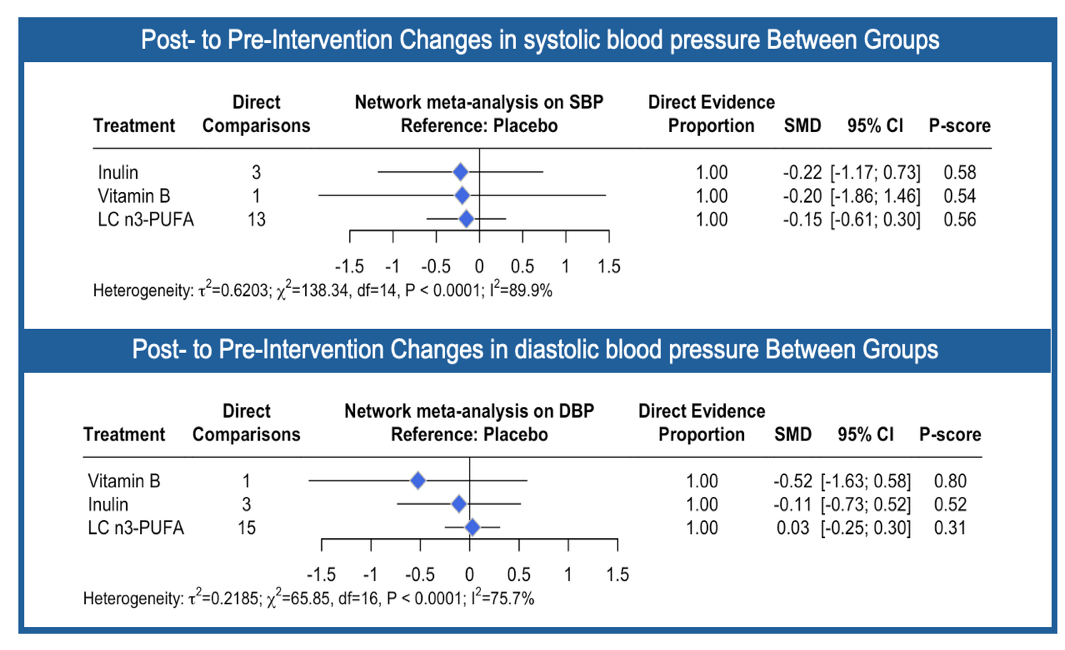

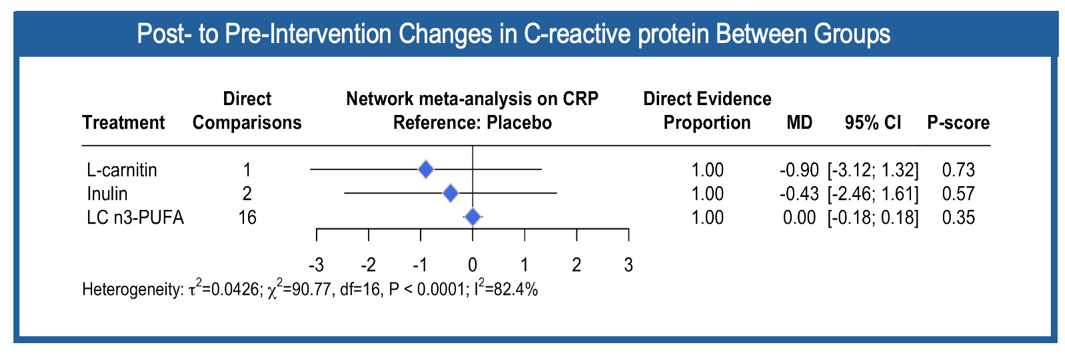


# **Figure S13**. Network meta-analysis results comparing the effects of different nutraceutical interventions compared to placebo (reference) on (top) systolic blood pressure, and (bottom) diastolic blood pressure. The number of direct comparisons for each treatment group is listed, alongside the standardized mean difference (SMD), 95% confidence intervals (CI), and P-scores, which range from 0 (least effective) to 1 (most effective). Proportion values indicate the contribution of direct evidence to the network estimates. Heterogeneity statistics (τ², χ², I²) are reported below each graph to assess consistency across included studies.


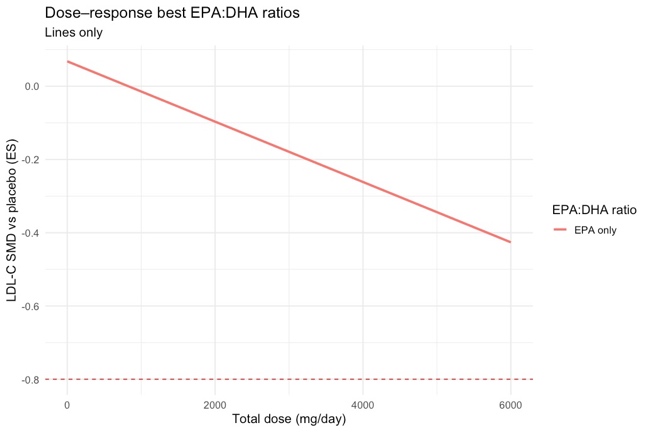

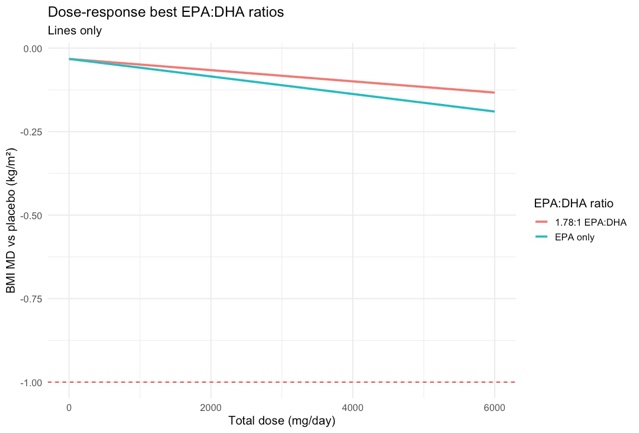

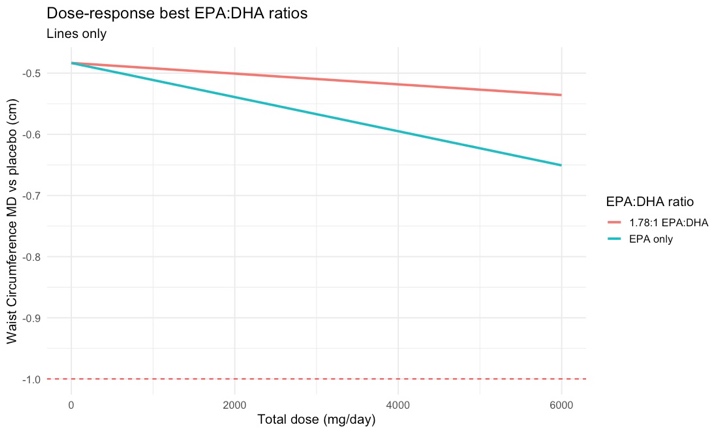

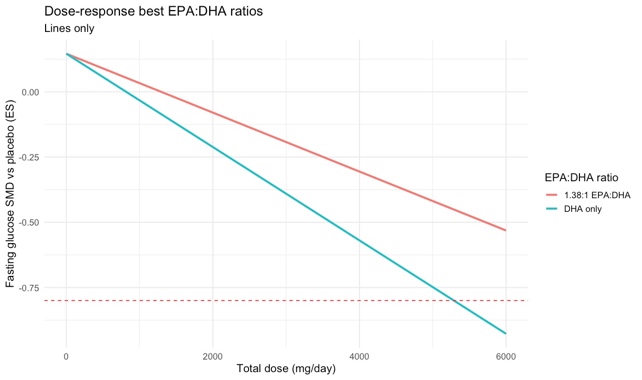

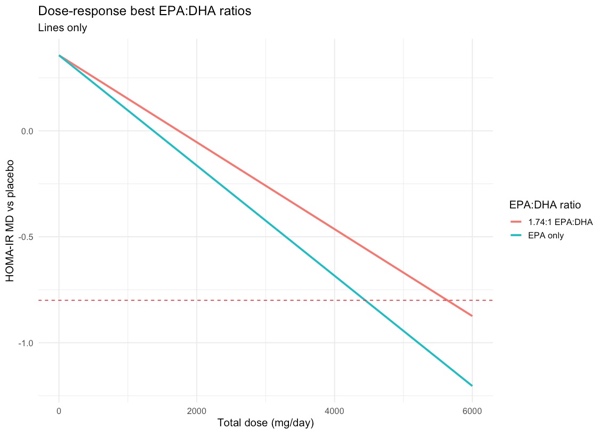

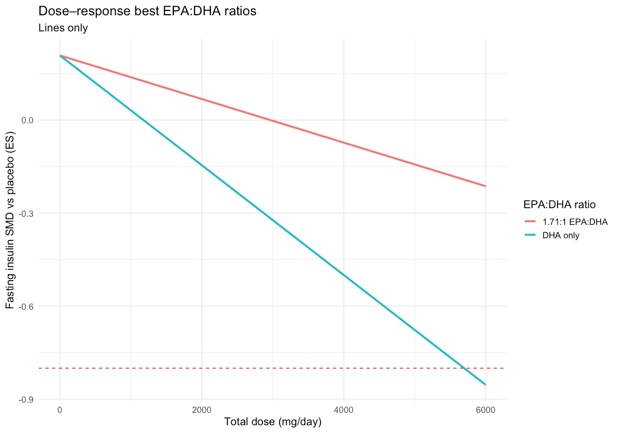

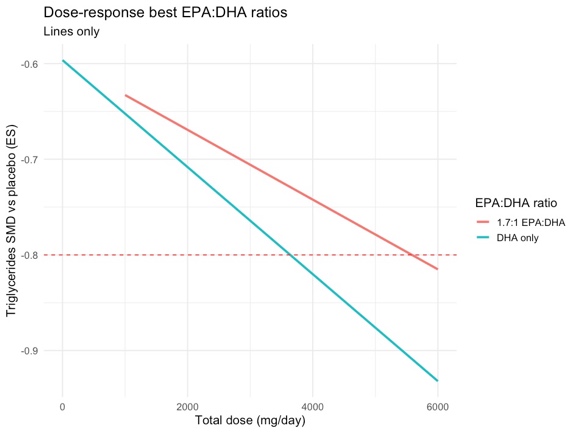

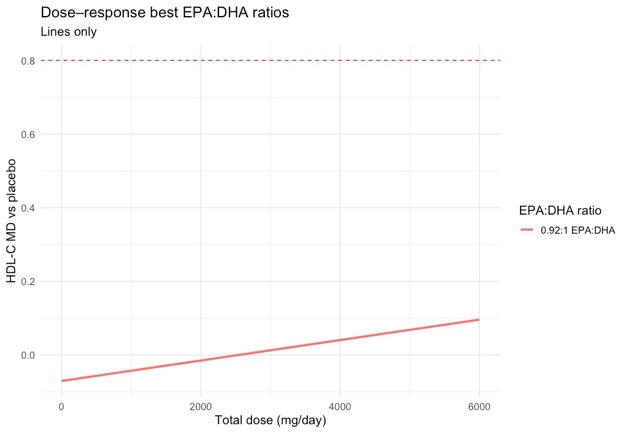

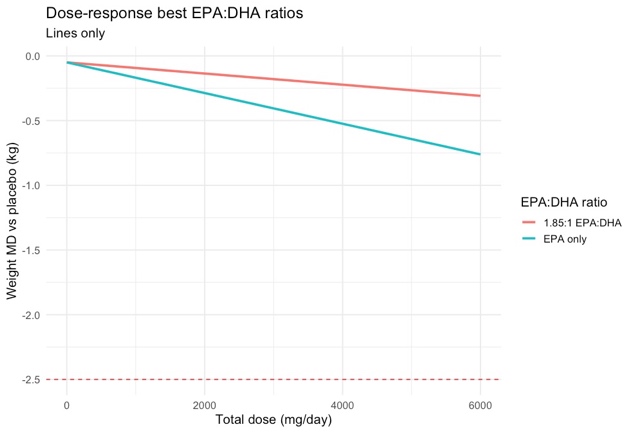


# **Figure S14**. Multivariate dose-response network meta-analysis of DHA and EPA supplementation effects on body composition (top), lipid profile (middle) and glycemic profile (bottom) after 12 weeks compared to placebo.


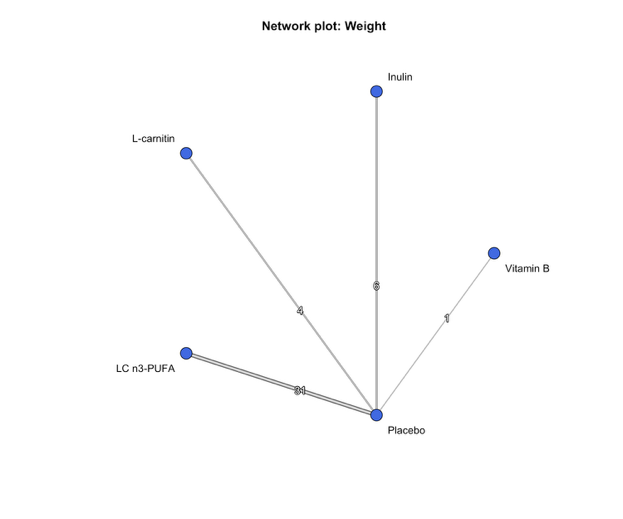

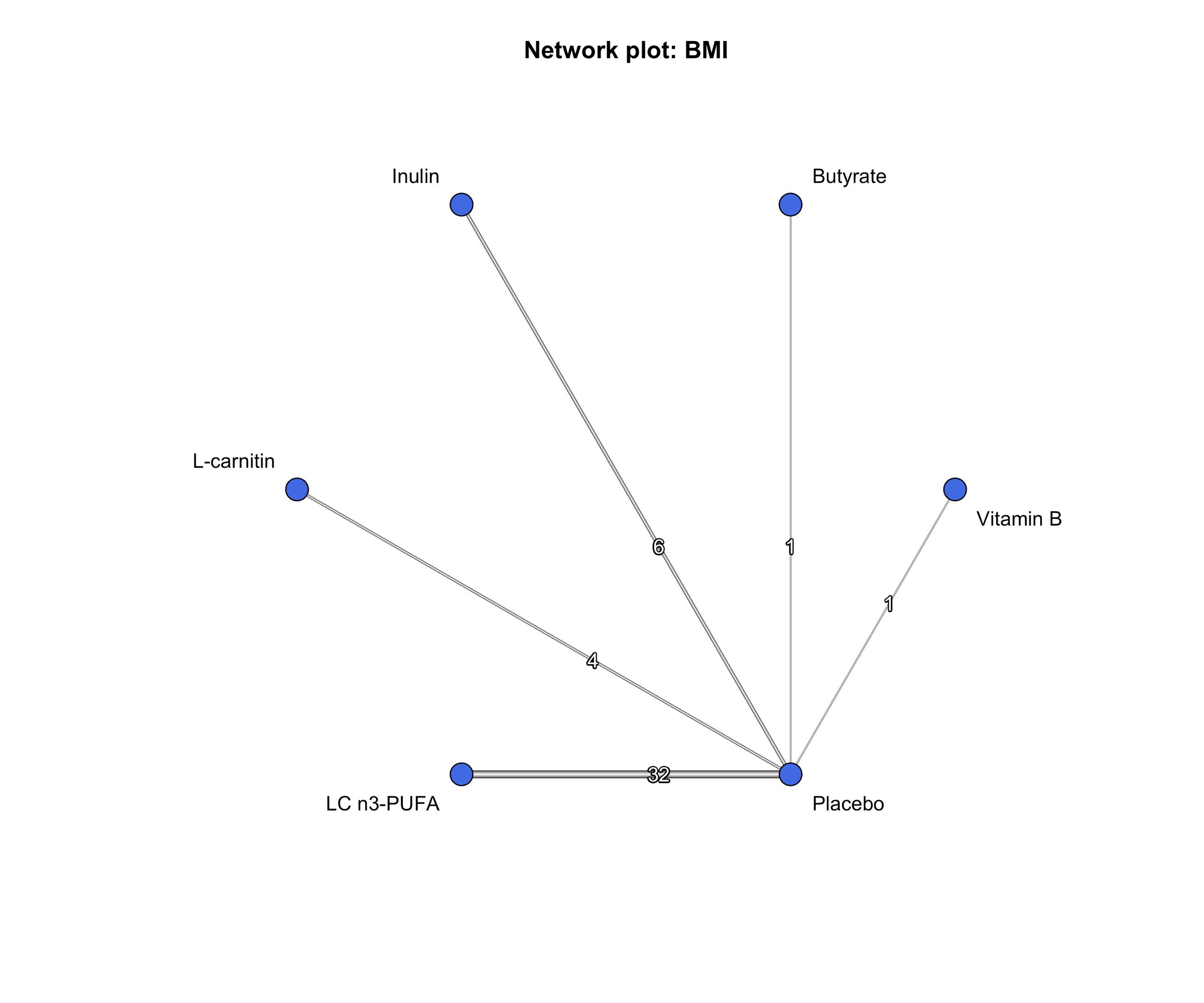

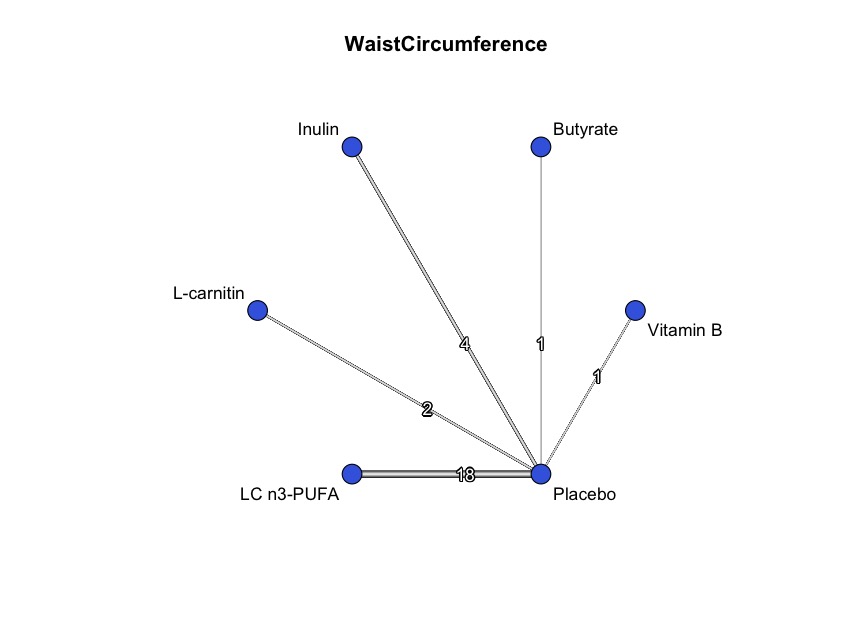

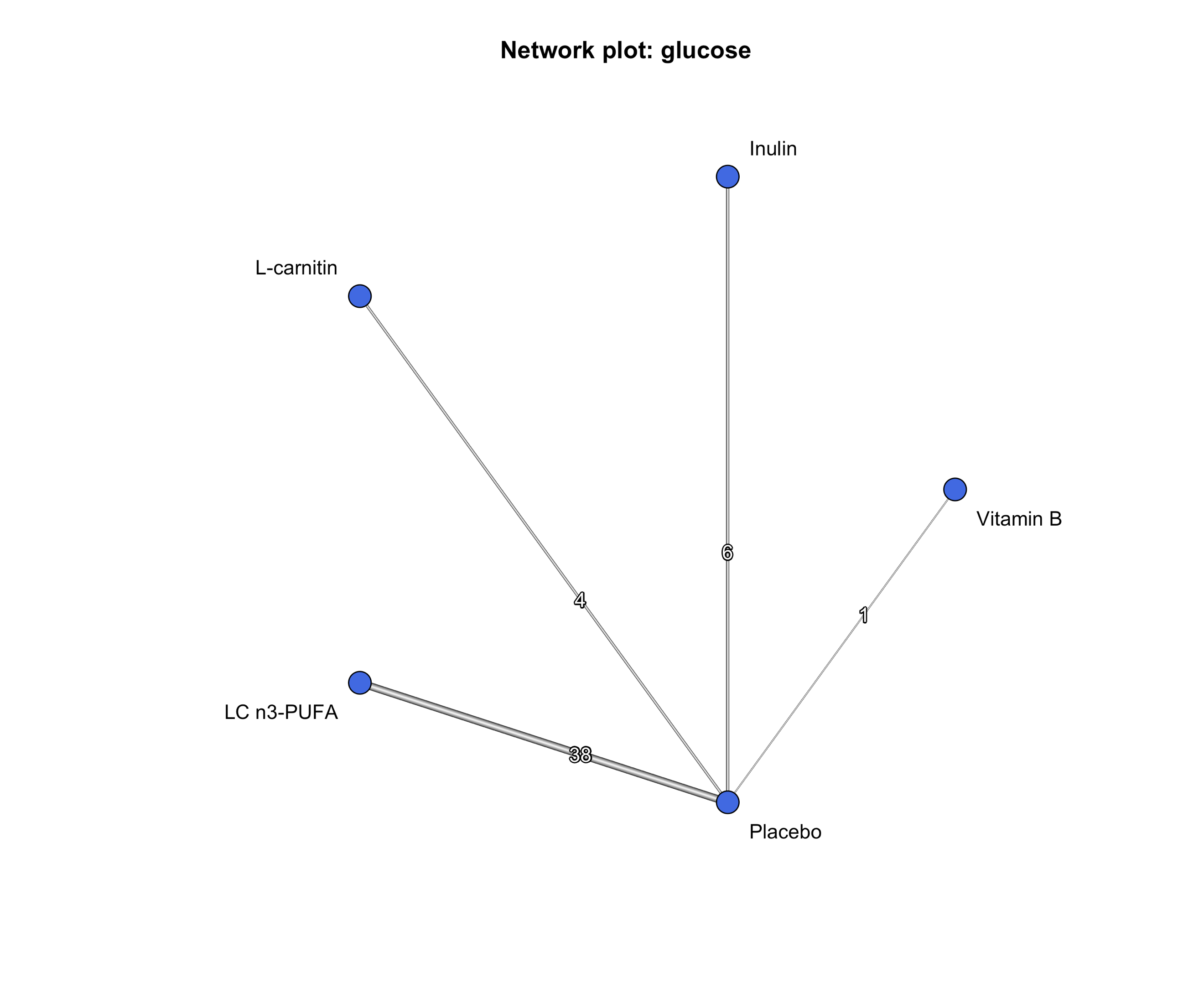

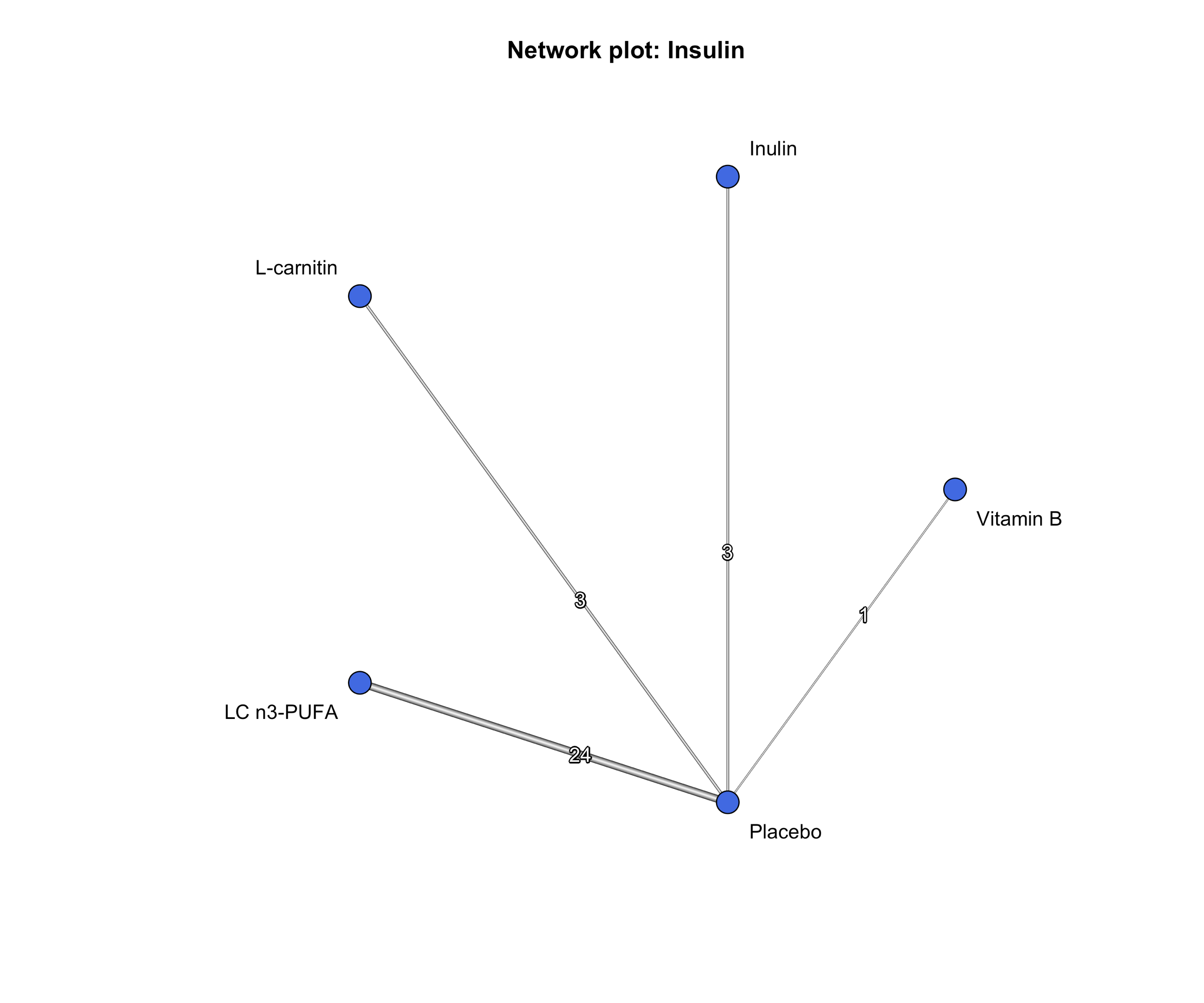

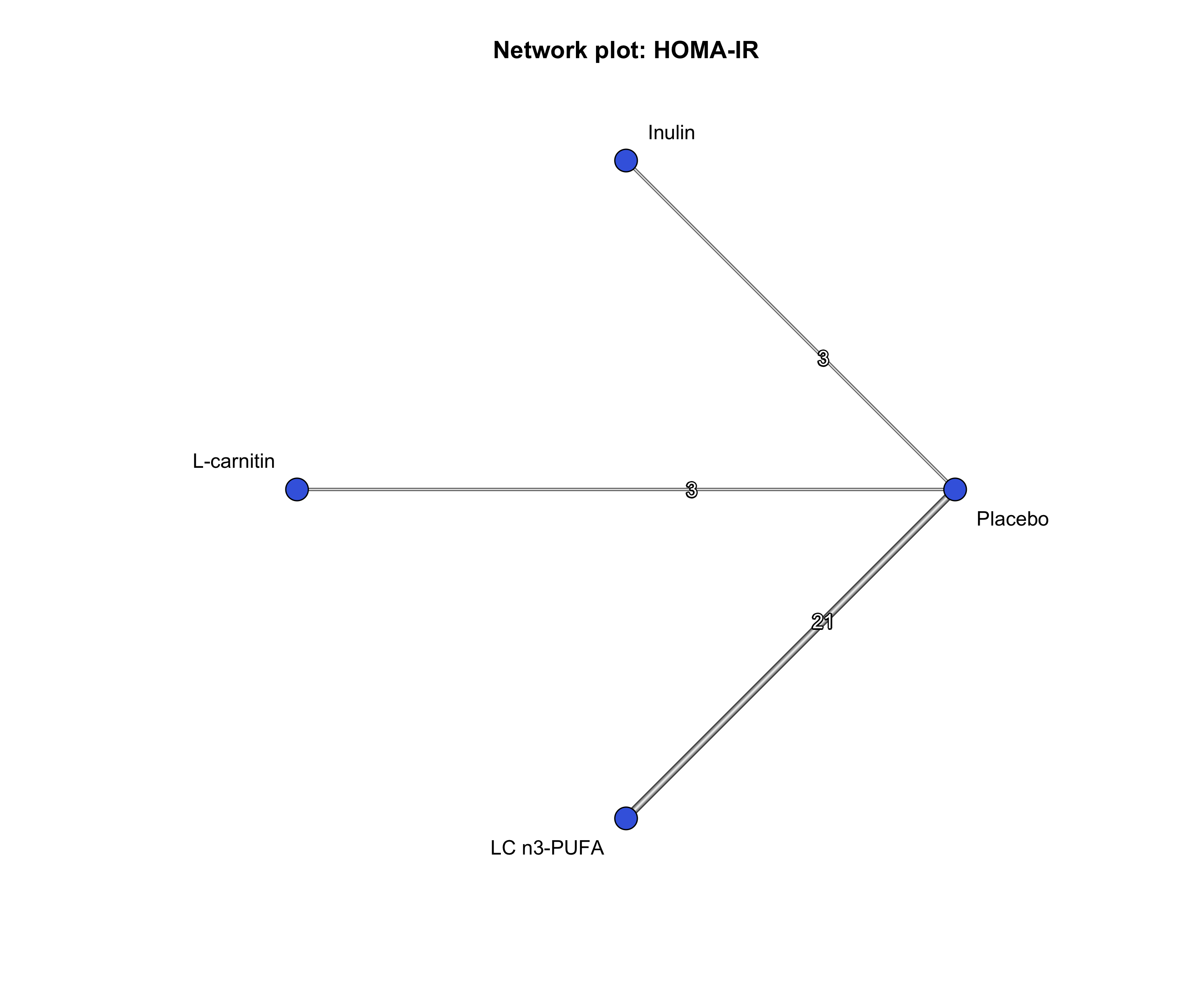

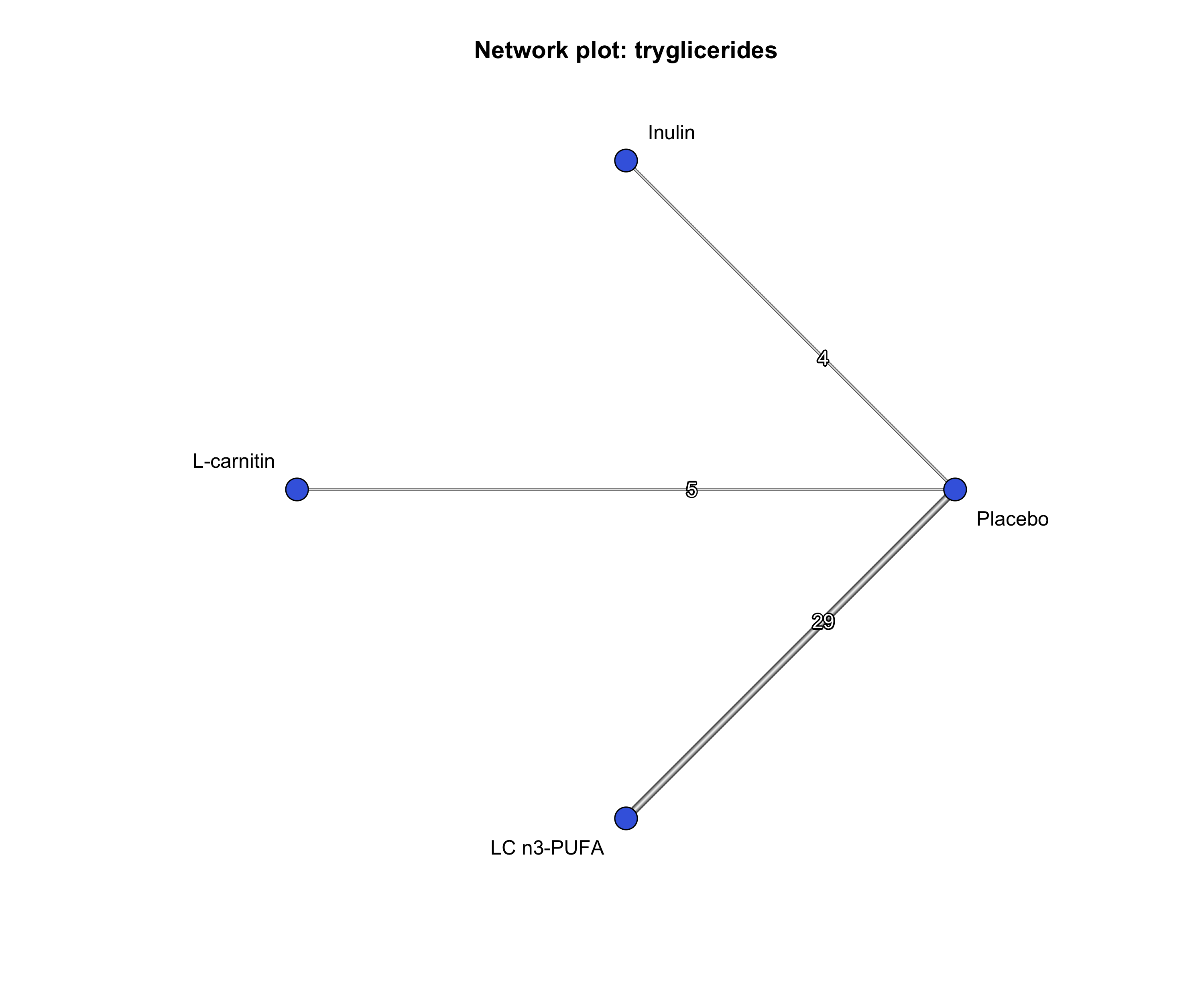

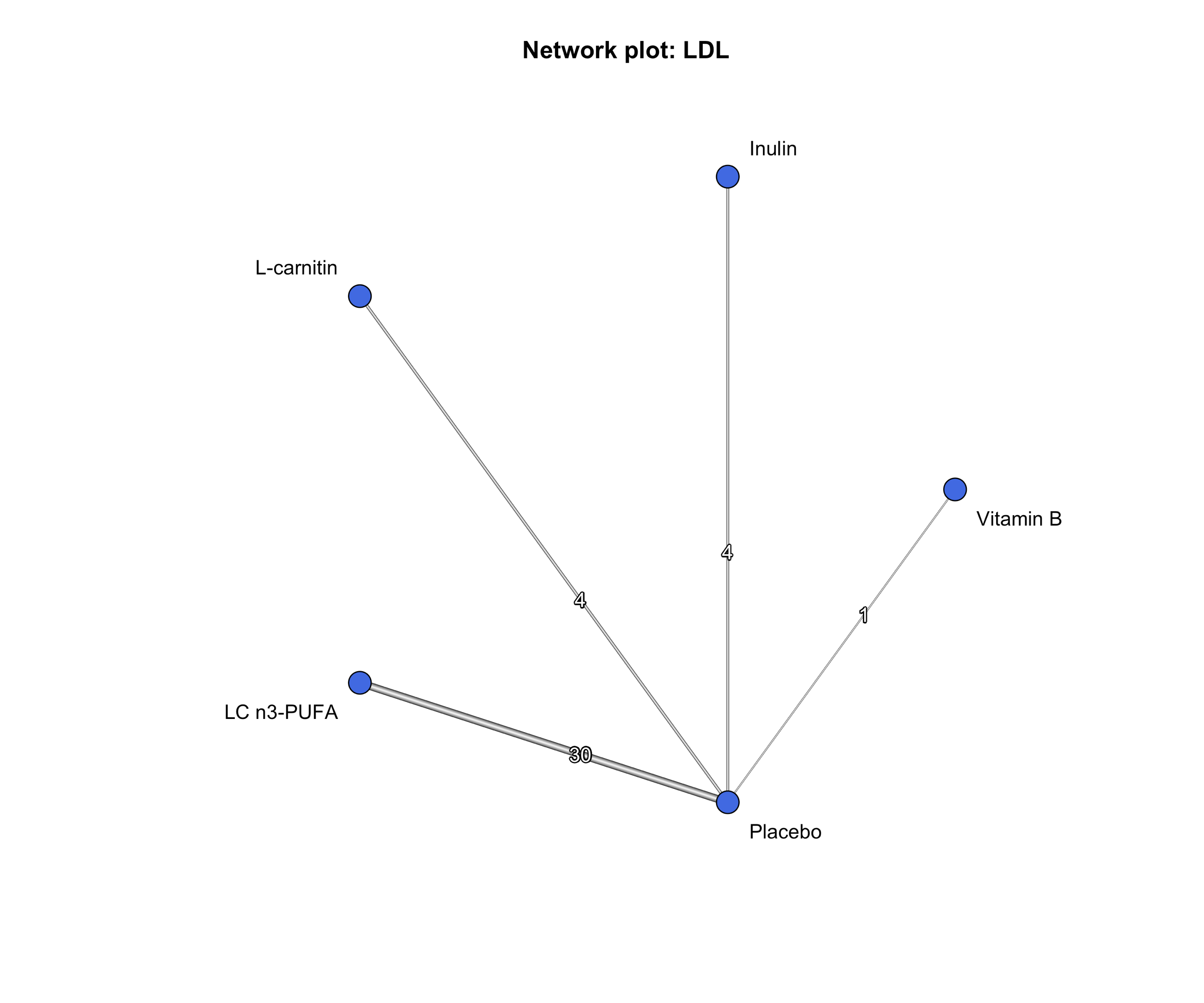

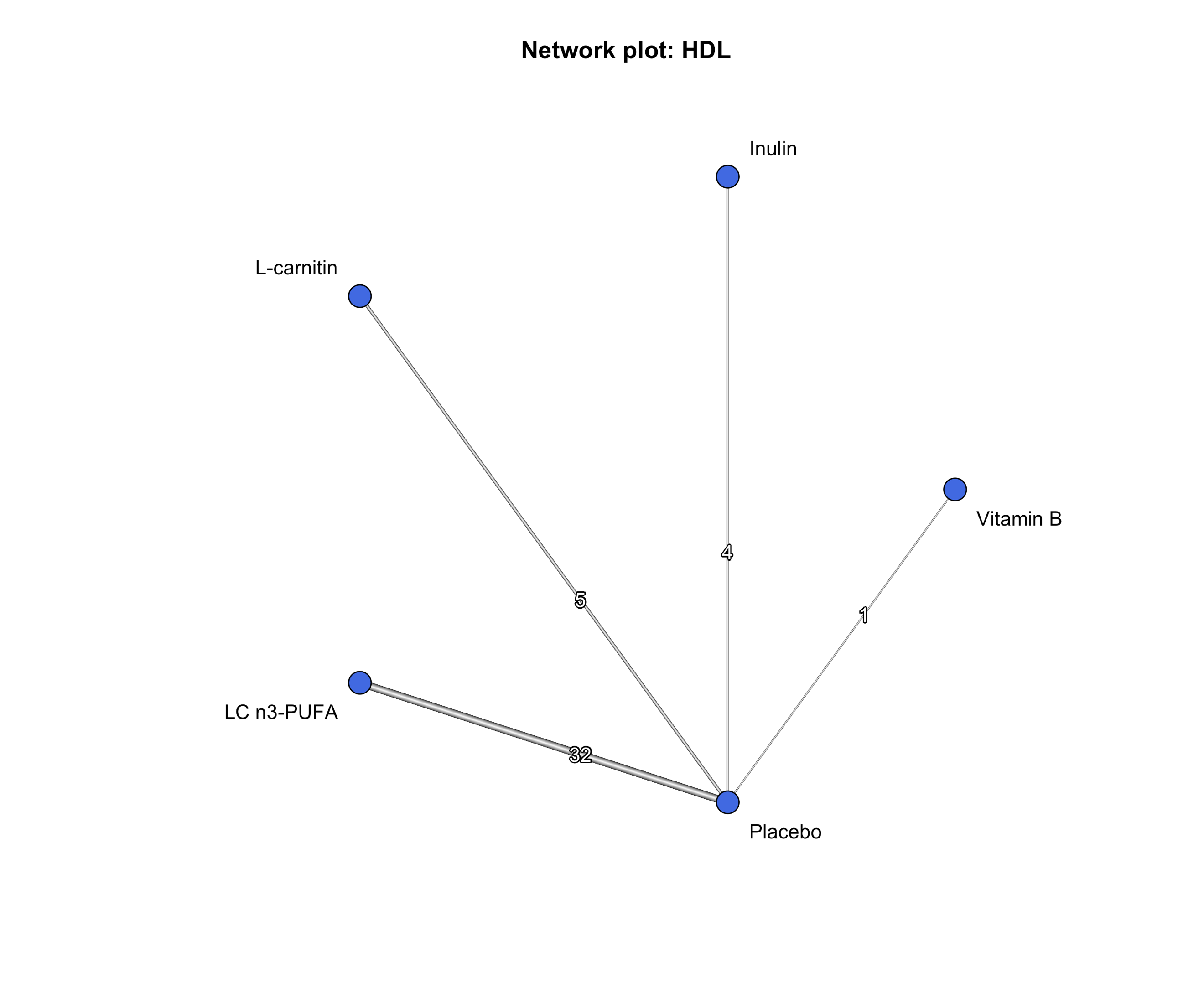


# **Figure S14**. Network diagrams illustrating the available comparisons for anthropometric and metabolic outcomes. Line thickness reflects the number of studies directly comparing each intervention pair, while node size corresponds to the total number of participants in each treatment group. Each node indicates a treatment group: placebo or the type of supplementation.


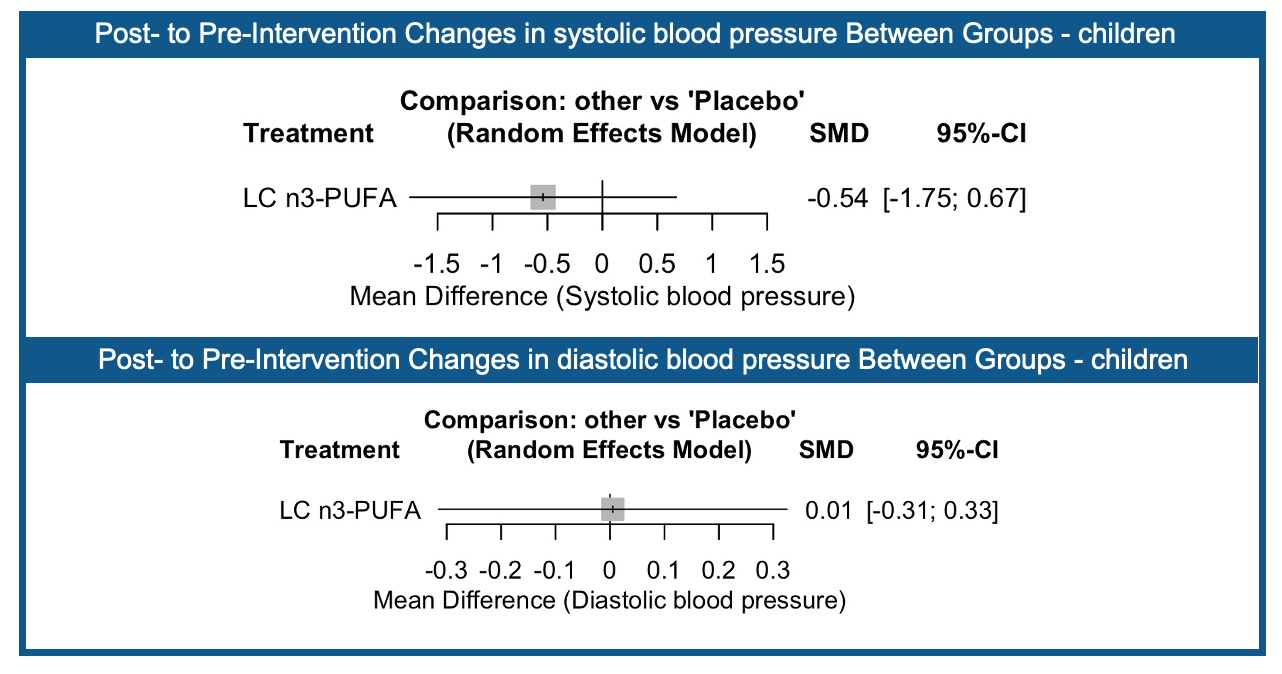


# **Figure S15**. Network meta-analysis results comparing the effects of different nutraceutical interventions on children compared to placebo (reference) on (top) systolic blood pressure, and (bottom) diastolic blood pressure. The number of direct comparisons for each treatment group is listed, alongside the standardized mean difference (SMD), 95% confidence intervals (CI), and P-scores, which range from 0 (least effective) to 1 (most effective). Proportion values indicate the contribution of direct evidence to the network estimates. Heterogeneity statistics (τ², χ², I²) are reported below each graph to assess consistency across included studies.


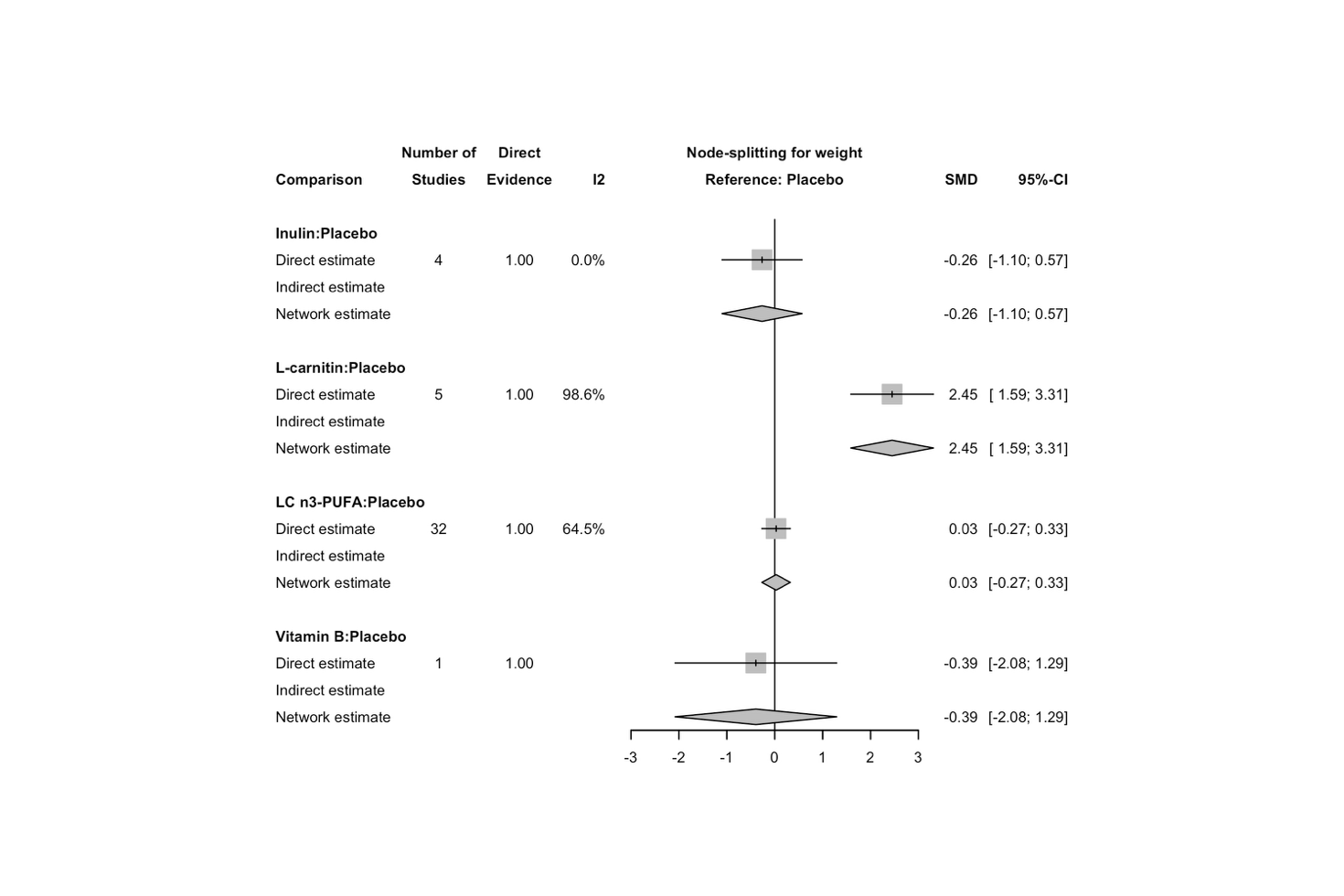


# **Figure S16.** Node-splitting analysis for body weight (placebo-controlled comparisons).


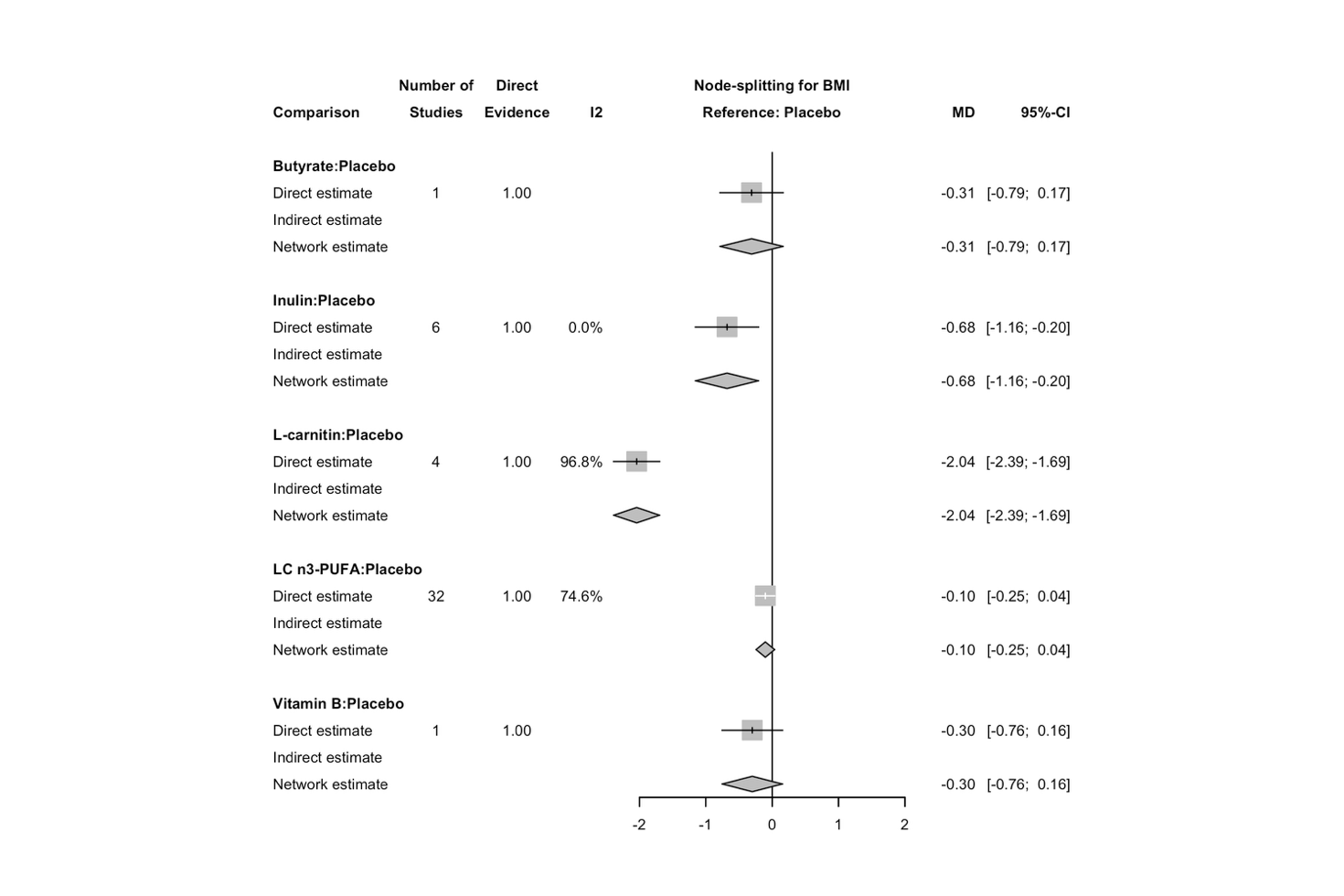


# **Figure S17.** Node-splitting analysis for body mass index (BMI, placebo-controlled comparisons).


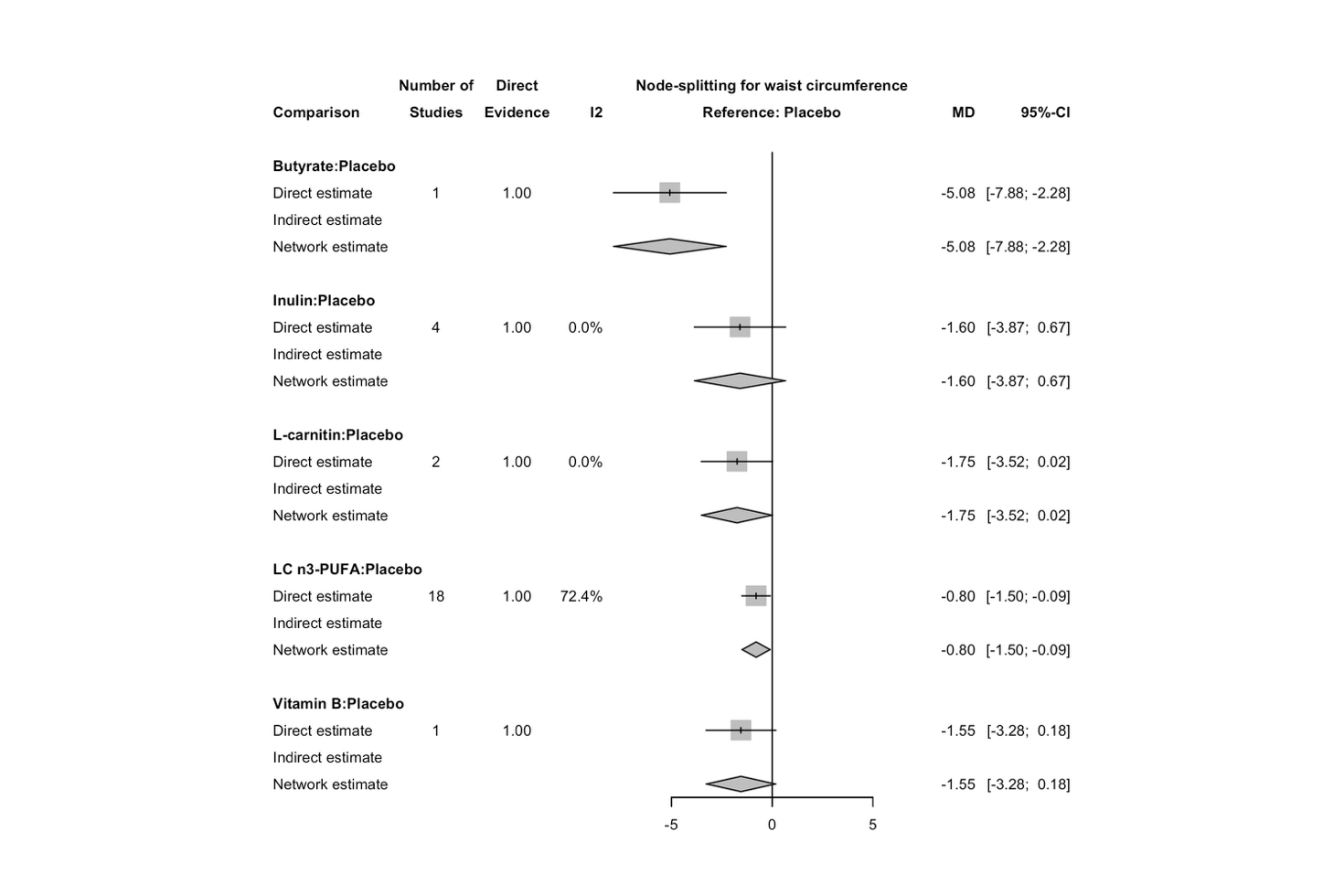


# **Figure S18.** Node-splitting analysis for waist circumference (placebo-controlled comparisons).


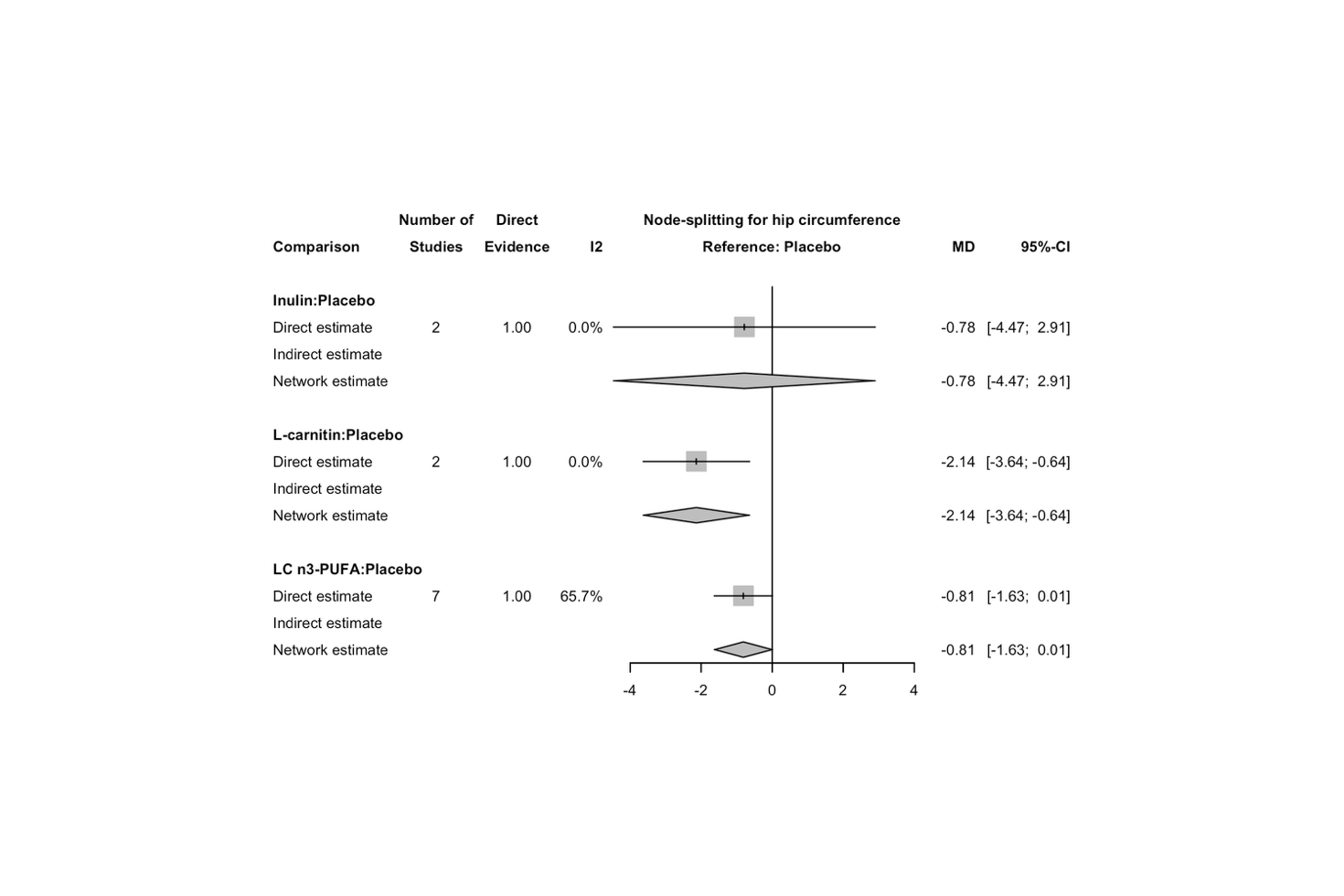


# **Figure S19.** Node-splitting analysis for hip circumference (placebo-controlled comparisons).


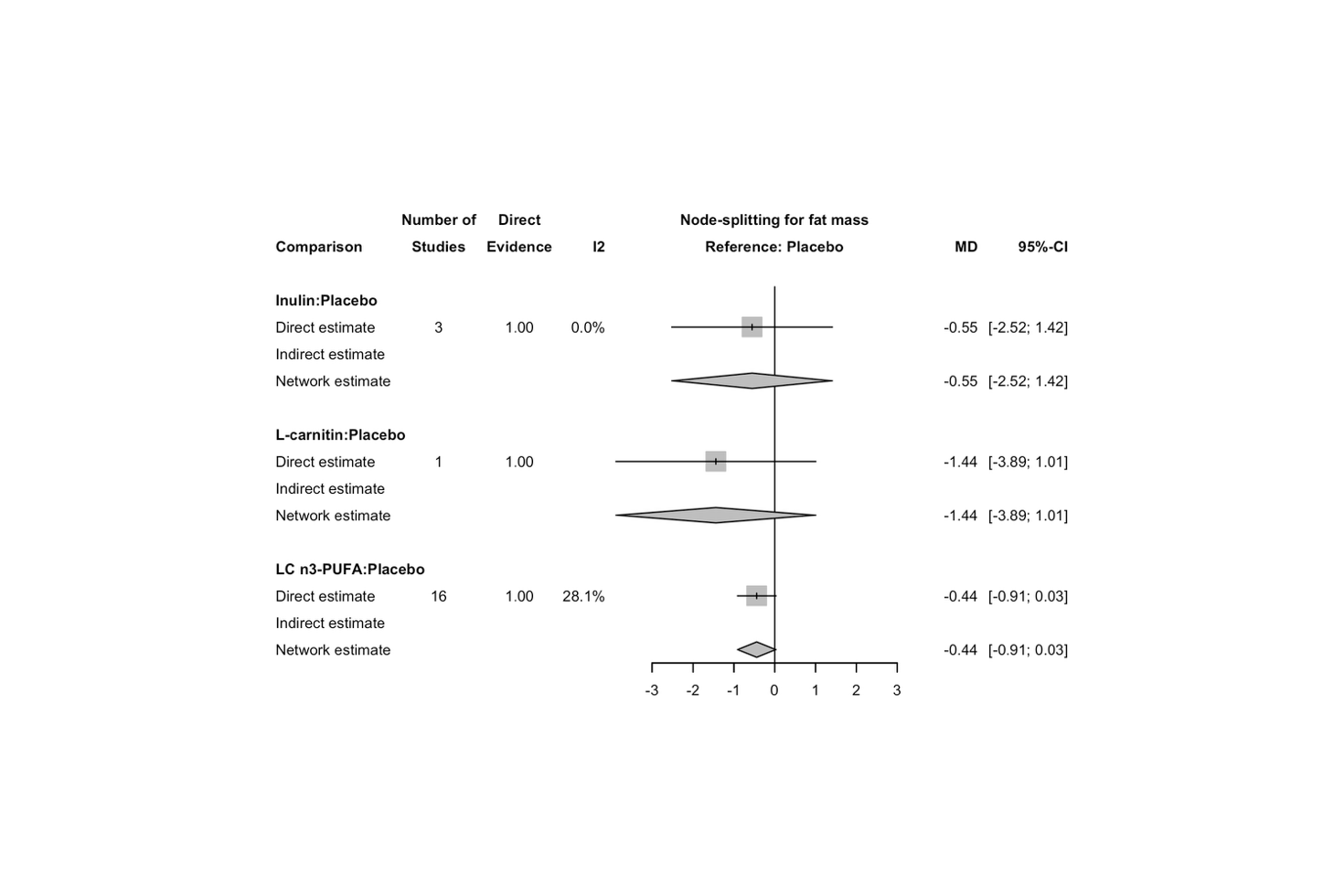


# **Figure S20.** Node-splitting analysis for fat mass (placebo-controlled comparisons).


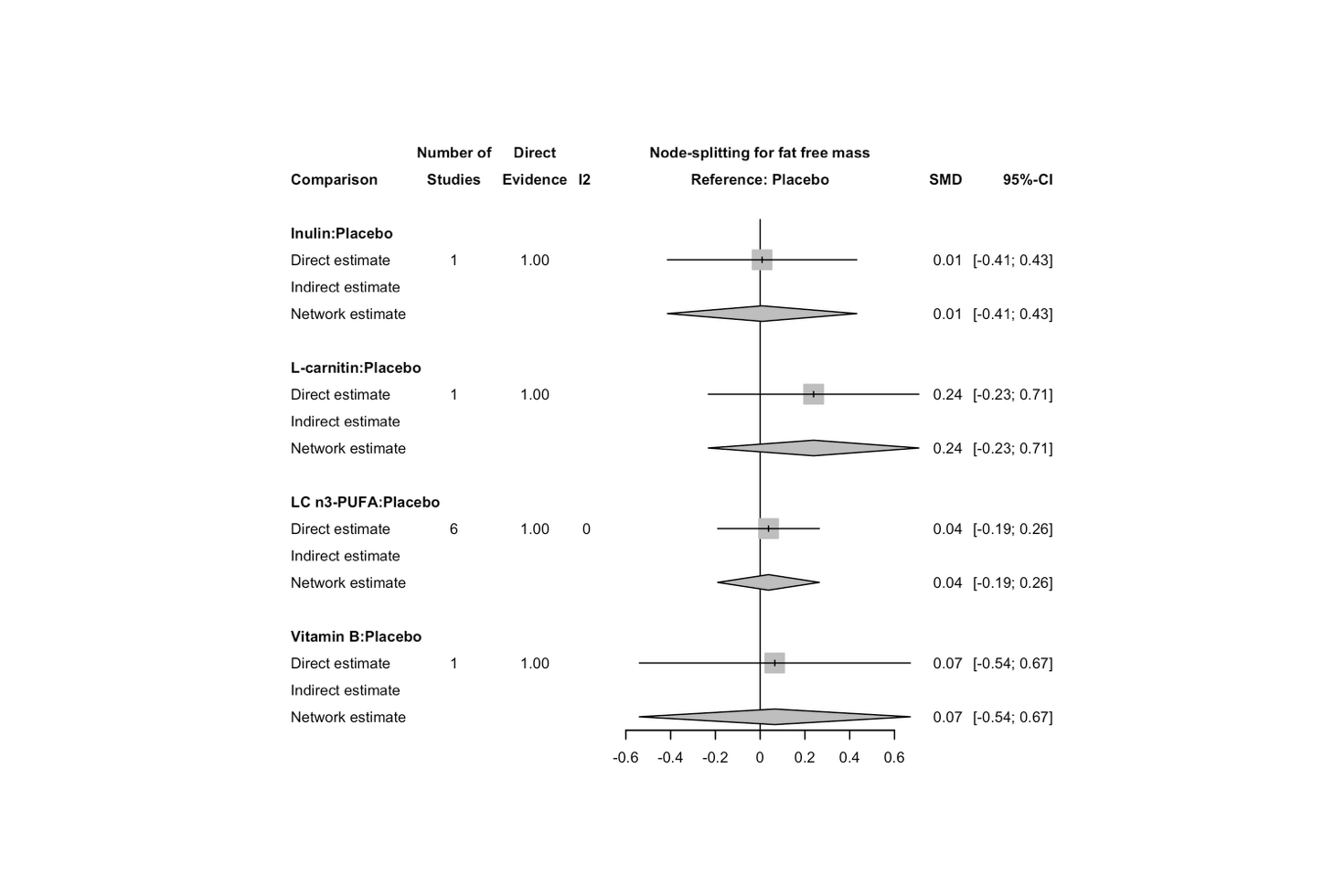


# **Figure S21.** Node-splitting analysis for fat free mass (placebo-controlled comparisons).


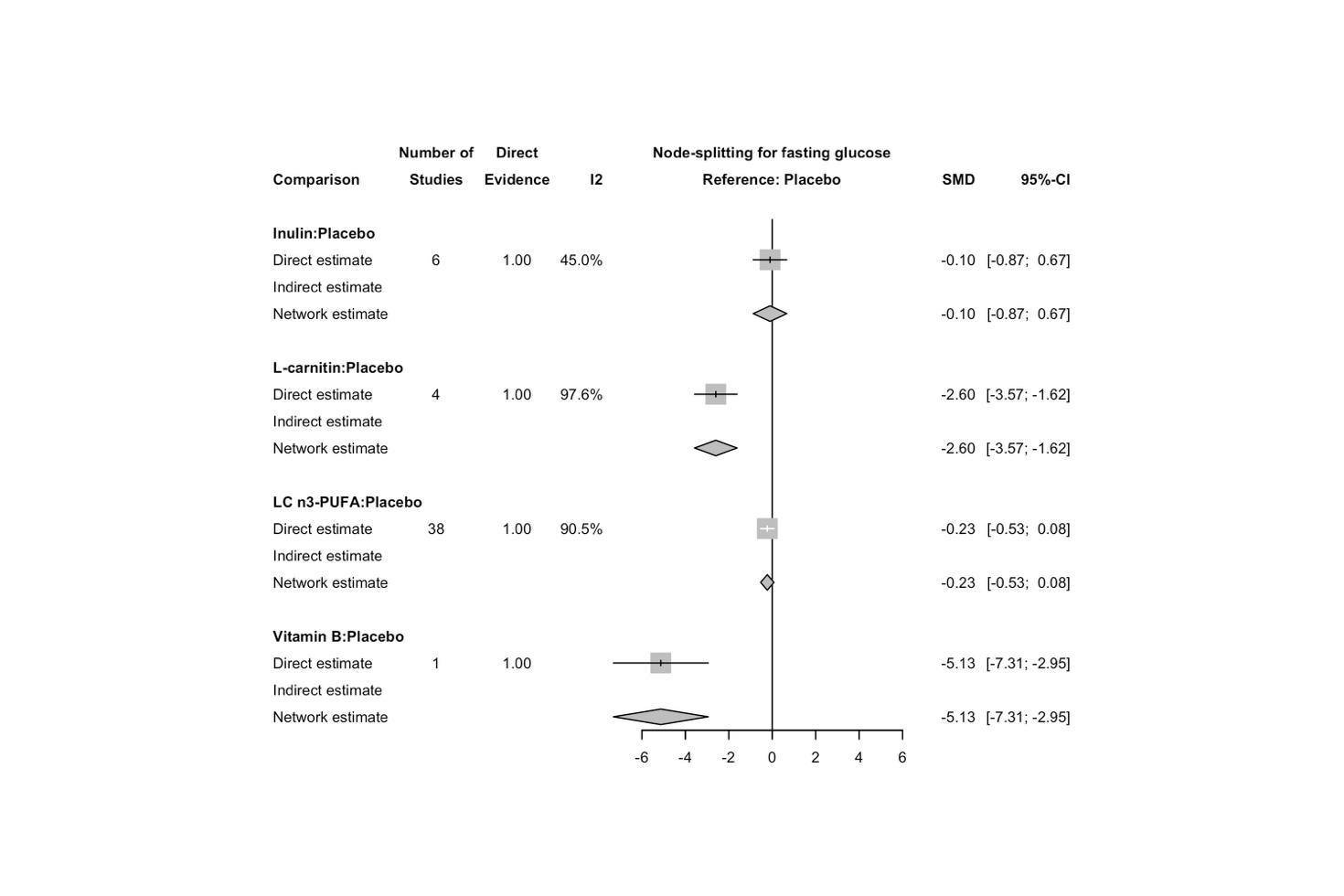


# **Figure S22.** Node-splitting analysis for fasting glucose (placebo-controlled comparisons).


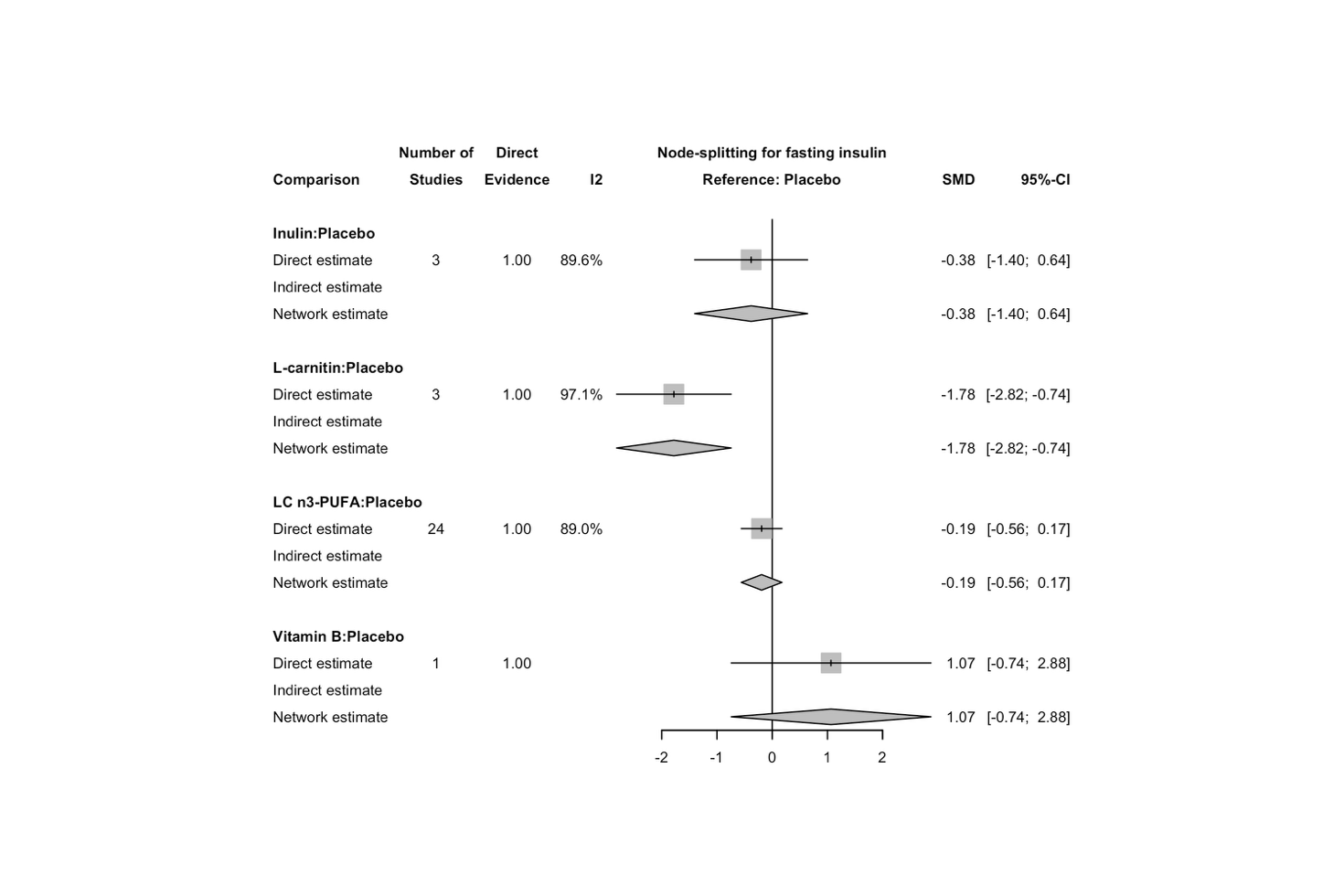


# **Figure S23.** Node-splitting analysis for fasting insulin (placebo-controlled comparisons).


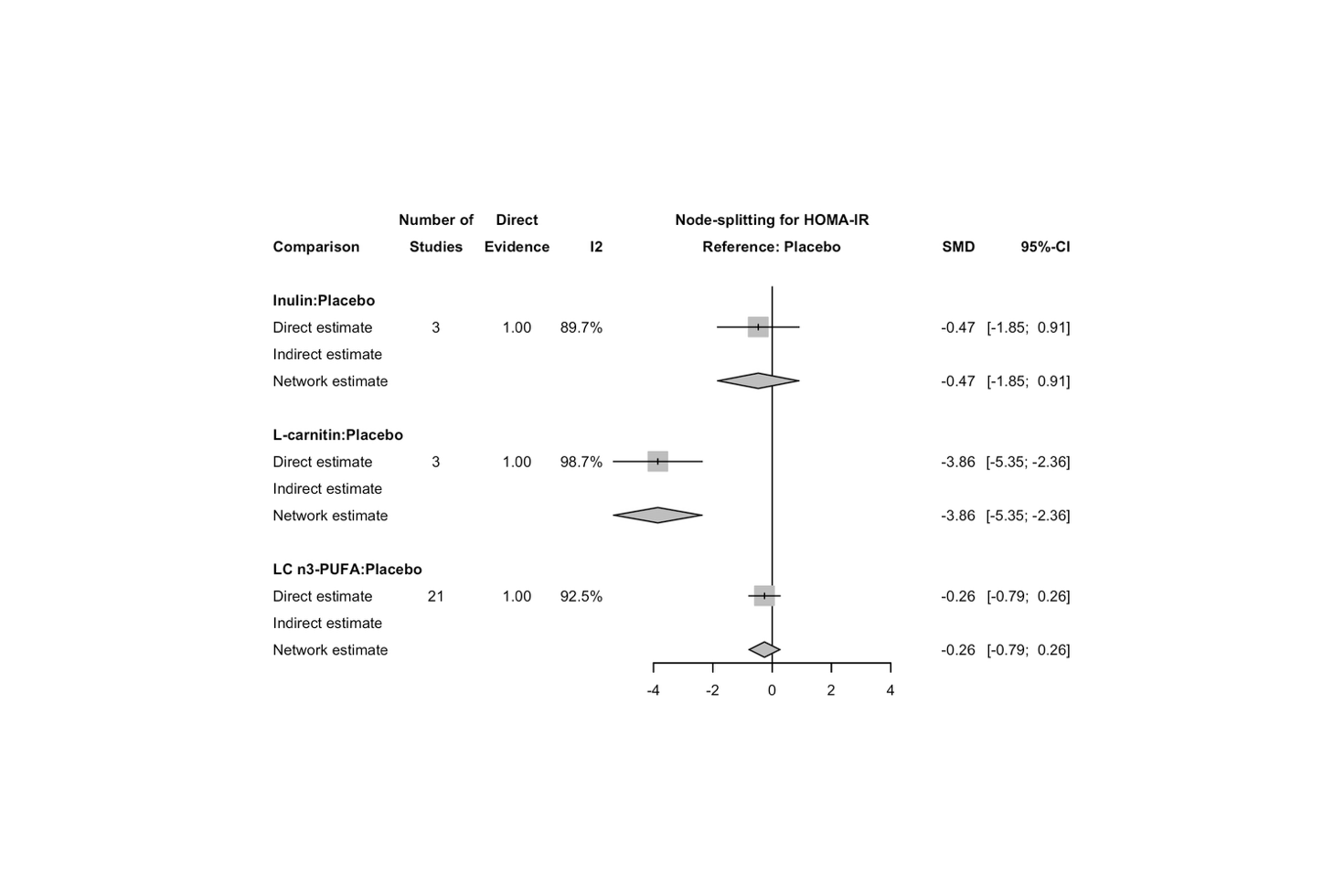


# **Figure S24.** Node-splitting analysis for homeostatic model assessment of insulin resistance (HOMA-IR, placebo-controlled comparisons).

**
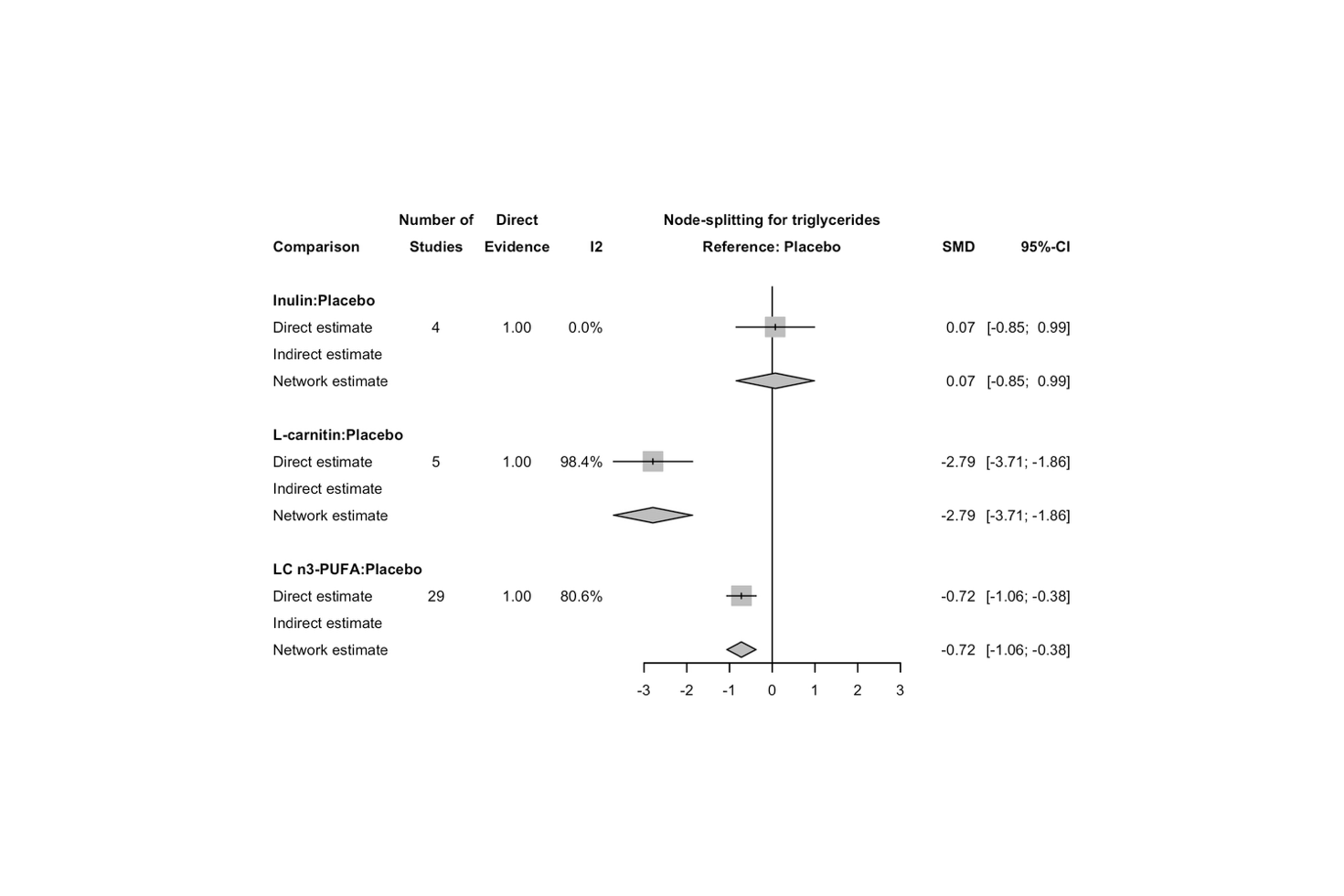
**

# **Figure S23.** Node-splitting analysis for triglycerides (placebo-controlled comparisons).


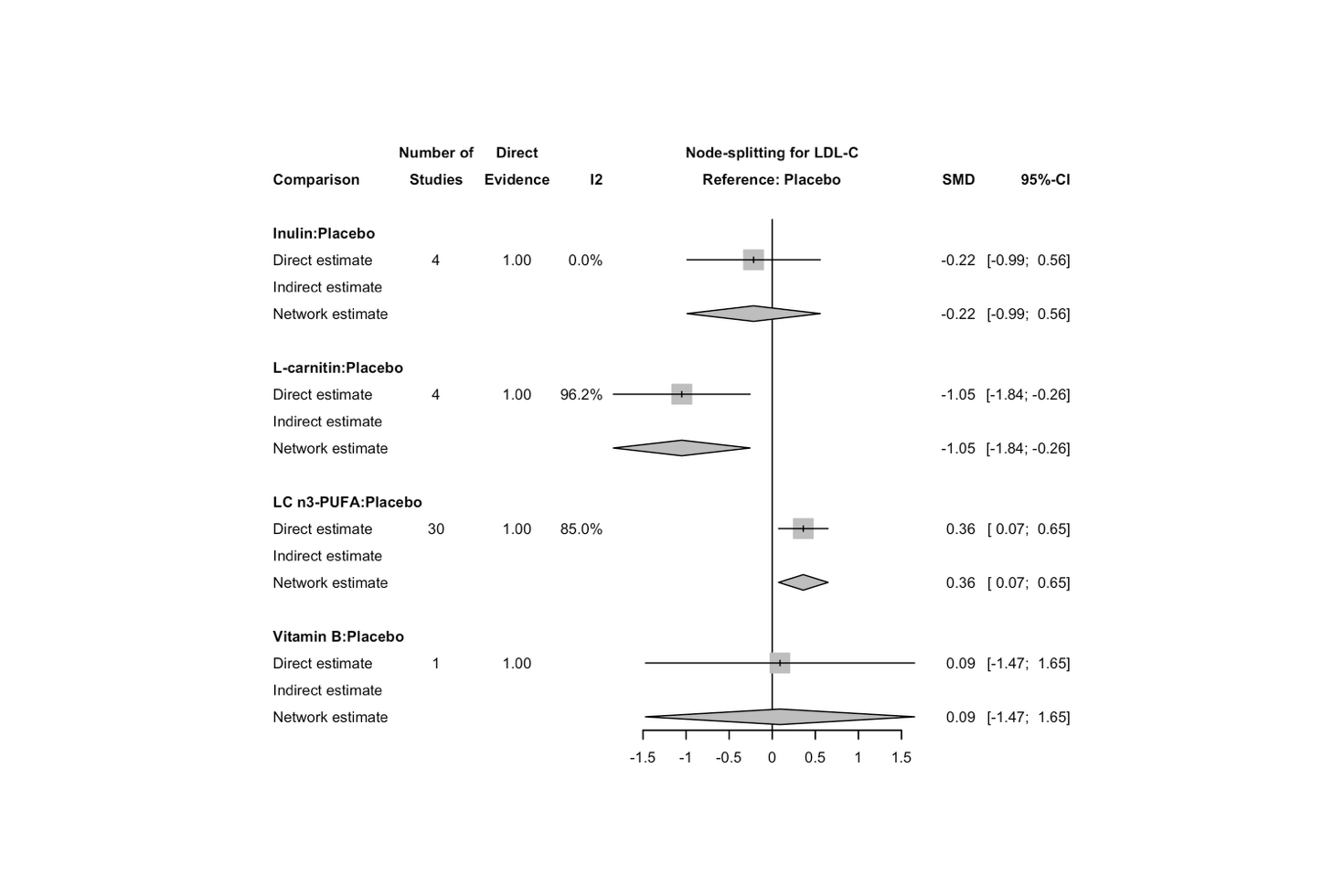


# **Figure S25.** Node-splitting analysis for low-density lipoprotein (LDL-C, placebo-controlled comparisons).


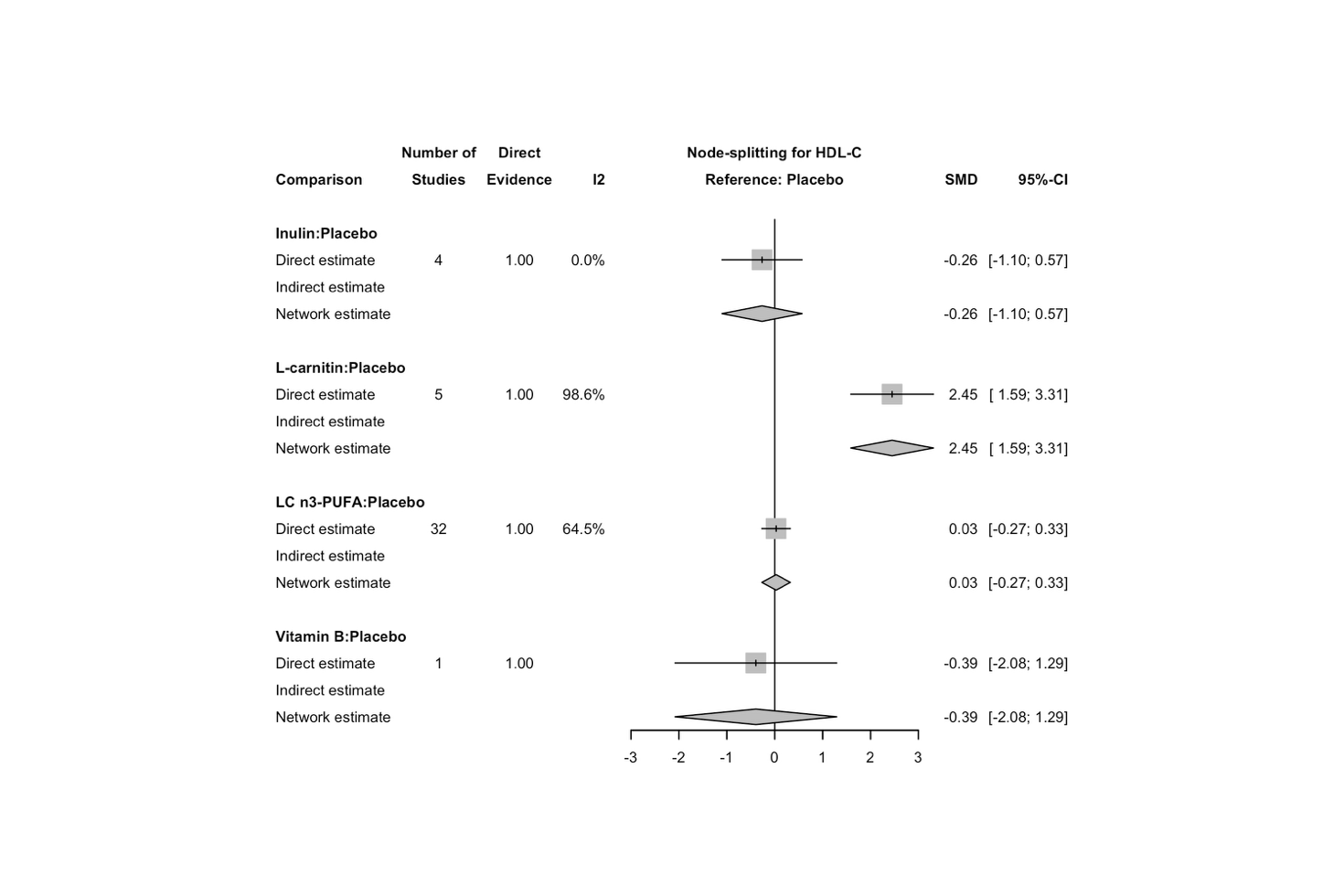


# **Figure S25.** Node-splitting analysis for high-density lipoprotein (HDL-C, placebo-controlled comparisons).

# **Table S1.** PRISMA NMA Checklist of Items to Include When Reporting A Systematic Review Involving a Network Meta-analysis

| **Section/Topic** | **Item #** | **Checklist Item** | **Reported on Page #** |
| --- | --- | --- | --- |
| **TITLE** |  |  |  |
| Title | 1 | Identify the report as a systematic review *incorporating a network meta-analysis (or related form of meta-analysis).* | 1 |
|  |  |  |  |
| **ABSTRACT** |  |  |  |
| Structured summary | 2 | Provide a structured summary including, as applicable:  **Background:** main objectives  **Methods:** data sources; study eligibility criteria, participants, and interventions; study appraisal; and *synthesis methods, such as network meta-analysis.*  **Results:** number of studies and participants identified; summary estimates with corresponding confidence/credible intervals; *treatment rankings may also be discussed. Authors may choose to summarize pairwise comparisons against a chosen treatment included in their analyses for brevity.*  **Discussion/Conclusions:** limitations; conclusions and implications of findings.  **Other:** primary source of funding; systematic review registration number with registry name. | 1-2 |
|  |  |  |  |
| **INTRODUCTION** |  |  |  |
| Rationale | 3 | Describe the rationale for the review in the context of what is already known*, including mention of why a network meta-analysis has been conducted.* | 3 |
| Objectives | 4 | Provide an explicit statement of questions being addressed, with reference to participants, interventions, comparisons, outcomes, and study design (PICOS). | 3-4 |
|  |  |  |  |
| **METHODS** |  |  |  |
| Protocol and registration | 5 | Indicate whether a review protocol exists and if and where it can be accessed (e.g., Web address); and, if available, provide registration information, including registration number. | 4 |
| Eligibility criteria | 6 | Specify study characteristics (e.g., PICOS, length of follow-up) and report characteristics (e.g., years considered, language, publication status) used as criteria for eligibility, giving rationale. *Clearly describe eligible treatments included in the treatment network, and note whether any have been clustered or merged into the same node (with justification).* | 4; Supp. |
| Information sources | 7 | Describe all information sources (e.g., databases with dates of coverage, contact with study authors to identify additional studies) in the search and date last searched. | 4; Supp. |
| Search | 8 | Present full electronic search strategy for at least one database, including any limits used, such that it could be repeated. | 4; Supp. |
| Study selection | 9 | State the process for selecting studies (i.e., screening, eligibility, included in systematic review, and, if applicable, included in the meta-analysis). | 5-6 |
| Data collection process | 10 | Describe method of data extraction from reports (e.g., piloted forms, independently, in duplicate) and any processes for obtaining and confirming data from investigators. | 5 |
| Data items | 11 | List and define all variables for which data were sought (e.g., PICOS, funding sources) and any assumptions and simplifications made. | 6; Supp. |
| **Geometry of the network** | **S1** | Describe methods used to explore the geometry of the treatment network under study and potential biases related to it. This should include how the evidence base has been graphically summarized for presentation, and what characteristics were compiled and used to describe the evidence base to readers. | 6-7 |
| Risk of bias within individual studies | 12 | Describe methods used for assessing risk of bias of individual studies (including specification of whether this was done at the study or outcome level), and how this information is to be used in any data synthesis. | 6 |
| Summary measures | 13 | State the principal summary measures (e.g., risk ratio, difference in means). *Also describe the use of additional summary measures assessed, such as treatment rankings and surface under the cumulative ranking curve (SUCRA) values, as well as modified approaches used to present summary findings from meta-analyses.* | 6 |
| Planned methods of analysis | 14 | Describe the methods of handling data and combining results of studies for each network meta-analysis. This should include, but not be limited to:   - *Handling of multi-arm trials;* - *Selection of variance structure;* - *Selection of prior distributions in Bayesian analyses; and* - *Assessment of model fit.* | 6 |
| **Assessment of Inconsistency** | **S2** | Describe the statistical methods used to evaluate the agreement of direct and indirect evidence in the treatment network(s) studied. Describe efforts taken to address its presence when found. | 6-7 |
| Risk of bias across studies | 15 | Specify any assessment of risk of bias that may affect the cumulative evidence (e.g., publication bias, selective reporting within studies). | 5 |
| Additional analyses | 16 | Describe methods of additional analyses if done, indicating which were pre-specified. This may include, but not be limited to, the following:   - Sensitivity or subgroup analyses; - Meta-regression analyses; - *Alternative formulations of the treatment network; and* - *Use of alternative prior distributions for Bayesian analyses (if applicable).* | 6 |
|  |  |  |  |
| **RESULTS†** |  |  |  |
| Study selection | 17 | Give numbers of studies screened, assessed for eligibility, and included in the review, with reasons for exclusions at each stage, ideally with a flow diagram. | 6-7 |
| **Presentation of network structure** | **S3** | Provide a network graph of the included studies to enable visualization of the geometry of the treatment network. | Supp. |
| **Summary of network geometry** | **S4** | Provide a brief overview of characteristics of the treatment network. This may include commentary on the abundance of trials and randomized patients for the different interventions and pairwise comparisons in the network, gaps of evidence in the treatment network, and potential biases reflected by the network structure. | 7 |
| Study characteristics | 18 | For each study, present characteristics for which data were extracted (e.g., study size, PICOS, follow-up period) and provide the citations. | Supp. |
| Risk of bias within studies | 19 | Present data on risk of bias of each study and, if available, any outcome level assessment. | 7 |
| Results of individual studies | 20 | For all outcomes considered (benefits or harms), present, for each study: 1) simple summary data for each intervention group, and 2) effect estimates and confidence intervals. *Modified approaches may be needed to deal with information from larger networks.* | Supp. |
| Synthesis of results | 21 | Present results of each meta-analysis done, including confidence/credible intervals. *In larger networks, authors may focus on comparisons versus a particular comparator (e.g. placebo or standard care), with full findings presented in an appendix. League tables and forest plots may be considered to summarize pairwise comparisons.* If additional summary measures were explored (such as treatment rankings), these should also be presented. | 8-10; Supp. |
| **Exploration for inconsistency** | **S5** | Describe results from investigations of inconsistency. This may include such information as measures of model fit to compare consistency and inconsistency models, *P* values from statistical tests, or summary of inconsistency estimates from different parts of the treatment network. | Supp. |
| Risk of bias across studies | 22 | Present results of any assessment of risk of bias across studies for the evidence base being studied. | Supp. |
| Results of additional analyses | 23 | Give results of additional analyses, if done (e.g., sensitivity or subgroup analyses, meta-regression analyses*, alternative network geometries studied, alternative choice of prior distributions for Bayesian analyses,* and so forth). | Supp. |
|  |  |  |  |
| **DISCUSSION** |  |  |  |
| Summary of evidence | 24 | Summarize the main findings, including the strength of evidence for each main outcome; consider their relevance to key groups (e.g., healthcare providers, users, and policy-makers). | 18 |
| Limitations | 25 | Discuss limitations at study and outcome level (e.g., risk of bias), and at review level (e.g., incomplete retrieval of identified research, reporting bias). *Comment on the validity of the assumptions, such as transitivity and consistency. Comment on any concerns regarding network geometry (e.g., avoidance of certain comparisons).* | 19 |
| Conclusions | 26 | Provide a general interpretation of the results in the context of other evidence, and implications for future research. | 18-19 |
|  |  |  |  |
| **FUNDING** |  |  |  |
| Funding | 27 | Describe sources of funding for the systematic review and other support (e.g., supply of data); role of funders for the systematic review. This should also include information regarding whether funding has been received from manufacturers of treatments in the network and/or whether some of the authors are content experts with professional conflicts of interest that could affect use of treatments in the network. | Not applicable |

PICOS = population, intervention, comparators, outcomes, study design.

# **Table S2.** Search strategy employed for Pubmed/medline database.

| Database | Search Terms | Date Range |
| --- | --- | --- |
| Pubmed/Medline | (  ("Child"[MeSH Terms] OR "Adolescent"[MeSH Terms] OR child* OR adolescen* OR teen* OR pediatric*)  AND  ("Obesity"[MeSH Terms] OR obes* OR overweight OR "weight gain")  AND  (  "inulin"[MeSH Terms] OR inulin  OR "Butyrates"[MeSH Terms] OR "butyric acid" OR butyrate  OR "Fatty Acids, Omega-3"[MeSH Terms] OR "omega-3" OR omega 3 OR DHA OR EPA OR ALA  OR "Vitamin B Complex"[MeSH Terms] OR "vitamin B" OR "B complex"  OR "Carnitine"[MeSH Terms] OR carnitine  OR nutraceutical*  )  AND  (  "Weight Loss"[MeSH Terms] OR "weight loss" OR "body weight reduction"  OR "weight gain prevention"  OR "Metabolic Syndrome"[MeSH Terms] OR "Insulin Resistance"[MeSH Terms]  OR hyperinsulinemia OR hyperlipidemia OR dyslipidemia OR hypercholesterolemia OR hypertriglyceridemia  OR hypertension OR inflammation  OR "Blood Glucose"[MeSH Terms] OR "fasting blood glucose"  OR "Glycemic Control"[MeSH Terms] OR "glycemic control"  OR "Glycemic Index"[MeSH Terms] OR "HOMA-IR" OR "fasting insulin" OR "insulin sensitivity"  )  AND  (  "Randomized Controlled Trial"[Publication Type]  OR "Clinical Trial"[Publication Type]  OR randomized OR randomised OR "controlled study" OR "clinical study"  )  )  NOT "adult"[MeSH Terms] | 2010-01-01 to 2025-05-31 |

# **Table S3.** Descriptive characteristics of the studies included.

|  |  | Intervention type | | | | Study characteristics | | | |
| --- | --- | --- | --- | --- | --- | --- | --- | --- | --- |
| Study | Country | Study design | Weeks | Dosage supplement (mg/day) | Number of pills per day | Total sample size | Number of men | Number of women | Age group |
| Albert et al., 2015[58] | New Zealand | LC n3-PUFA vs placebo | 8 | 2000.0  EPA: 1150.0  DHA: 770.0 | 5 | 47 | 51 | 0 | Adults |
| Alblaji et al., 2025 [59] | UK | LC n3-PUFA vs placebo | 8 | 1140.0  EPA: 764.0  DHA: 376.0 | 4 | 41 | 16 | 25 | Adults |
| Ansari et al., 2017 [60] | Iran | LC n3-PUFA vs placebo | 12 | 3750.0  EPA: 1800.0  DHA: 900.0 | 3 | 43 | 25 | 18 | Adults |
| Baghban et al., 2021[61] | Iran | L-carnitin vs placebo | 12 | 1000.0 | Not specified | 70 | 0 | 70 | Adults |
| Bakker et al., 2023 [62] | The Netherlands | LC n3-PUFA vs placebo | 4 | 2000.0  EPA: 920.0  DHA: 760.0 | 2 | 56 | 0 | 56 | Adults |
| Boyraz et al., 2015 [63] | Turkey | LC n3-PUFA vs placebo | 52 | 1000.0 | 1 | 108 | 55 | 53 | Children |
| Bragt et al., 2011 [64] | Netherlands | LC n3-PUFA vs placebo | 6 | 3700.0  EPA: 1700.0  DHA: 1200.0 | 8 | 20 | 10 | 10 | Adults |
| Burhop et al., 2022 [65] | Germany | LC n3-PUFA vs placebo | 16 | 524.0  EPA: 138.0  DHA: 128.0 | 4 | 33 | 12 | 31 | Adults |
| Cicero et al., 2015 [66] | Italy | LC n3-PUFA vs placebo | 4 | 2000.0  EPA: 150.0  DHA: 90.0 | 2 | 25 | 14 | 12 | Adults |
| Citarrella et al., 2024 [67] | Italy | Inulin vs Placebo | 16 | 200.0 | 3 | 58 | 0 | 0 | Adults |
| Coppola et al., 2022 [68] | Italy | Butyrate vs placebo | 24 | 20.0 * body weight | Not specified | 54 | 23 | 31 | Children |
| Crochemore et al., 2012 [69] | Brazil | LC n3-PUFA vs placebo | 4 | 2500.0  EPA: 547.0  DHA: 325.5 | Oil | 41 | 0 | 41 | Adults |
| Dangardt et al., 2010 [70] | Sweden | LC n3-PUFA vs placebo | 12 | 1200.0  EPA: 930.0  DHA: 290.0 | 10 | 25 | 11 | 14 | Children |
| Dangardt et al., 2012 [71] | Sweden | LC n3-PUFA vs placebo | 12 | 1220.0  EPA: 930.0  DHA: 290.0 | 10 | 25 | 11 | 14 | Children |
| De Fina et al., 2011 [72] | USA | LC n3-PUFA vs placebo | 24 | 3600.0  EPA: 3000.0  DHA: 600.0 | 5 | 128 | 40 | 88 | Adults |
| De Luis et al., 2016 [73] | Spain | LC n3-PUFA vs placebo | 24 | DHA: 250.0 | Not specified | 29 | 15 | 14 | Adults |
| Dehghan et al., 2013 [74] | Iran | Inulin vs placebo | 8 | 10000.0 | Not specified | 49 | 0 | 54 | Adults |
| Derosa et al., 2016 [75] | Italy | LC n3-PUFA vs placebo | 72 | 3000.0  EPA: 1875.0  DHA: 1125.0 | 3 | 258 | 0 | 0 | Adults |
| Gammelmark et al., 2012 [76] | Denmark | LC n3-PUFA vs placebo | 6 | 2200.0  EPA: 1280.0  DHA: 960.0 | 2 | 49 | 24 | 26 | Adults |
| García-López et al., 2016 [77] | Mexico | LC n3-PUFA vs placebo | 4 | 2400.0  EPA: 1440.0  DHA: 960.0 | 2 | 0 | 0 | 0 | Adults |
| Golpour et al., 2020 [78] | Iran | LC n3-PUFA vs placebo | 10 | 2700.0  EPA: 1800.0  DHA: 900.0 | 3 | 61 | 39 | 22 | Adults |
| Goralska et al., 2022 [79] | Poland | LC n3-PUFA vs placebo | 12 | 2160.0  EPA: 360.0  DHA: 1800.0 | 3 | 62 | 16 | 46 | Adults |
| Hess et al., 2020 [80] | Denmark | Inulin vs placebo | 12 | 10000.0 | 1 | 86 | 40 | 76 | Adults |
| Huang et al., 2018 [81] | Mexico | LC n3-PUFA vs placebo | 12 | 3000.0  EPA: 2000.0  DHA: 1000.0 | 5 | 65 | 0 | 0 | Children |
| Huang et al., 2024 [82] | Taiwan | LC n3-PUFA vs placebo | 12 | 2130.0  EPA: 1280.0  DHA: 850.0 | 10 | 117 | 0 | 117 | Adults |
| Huerta et al., 2015 [83] | Spain | LC n3-PUFA vs placebo | 10 | 1340.4  EPA: 1299.0  DHA: 41.4 | 6 | 77 | 0 | 73 | Adults |
| Huerta et al., 2016 [84] | Spain | LC n3-PUFA vs placebo | 10 | EPA: 1300.0 | 3 | 40 | 0 | 40 | Adults |
| Iannelli et al., 2022 [85] | France | LC n3-PUFA vs placebo | 4 | 1650.0  EPA: 990.0  DHA: 660.0 | 3 | 37 | 14 | 28 | Adults |
| Itariu et al., 2012 [86] | Austria | LC n3-PUFA vs placebo | 8 | 3360.0  EPA: 1840.0  DHA: 1520.0 | 4 | 55 | 9 | 46 | Adults |
| Ito et al., 2014 [87] | Japan | LC n3-PUFA vs placebo | 12 | EPA: 1800.0 | 1 | 88 | 48 | 40 | Adults |
| Jamilian et al., 2019 [88] | Iran | L-carnitin vs placebo | 12 | 1000.0 | 1 | 54 | 0 | 54 | Adults |
| Juárez-López, 2013 [89] | Mexico | LC n3-PUFA vs placebo | 12 | 1800.0  EPA: 1080.0  DHA: 720.0 | 3 | 201 | 0 | 0 | Children |
| Keshavarz et al., 2018 [90] | Iran | LC n3-PUFA vs placebo | 12 | 1800.0  EPA: 1080.0  DHA: 720.0 | 6 | 45 | 0 | 45 | Adults |
| Lalia et al., 2015 [91] | USA | LC n3-PUFA vs placebo | 24 | 3900.0  EPA: 2700.0  DHA: 1200.0 | 4 | 25 | 8 | 23 | Adults |
| Lee et al., 2019 [92] | USA | LC n3-PUFA vs placebo | 8 | 4000.0  EPA: 1860.0  DHA: 1460.0 | Not specified | 25 | 0 | 0 | Adults |
| Leija-Martínez et al., 2019 [93] | Mexico | LC n3-PUFA vs placebo | 12 | 3000.0  EPA: 2000.0  DHA: 1000.0 | 5 | 130 | 0 | 0 | Children |
| López-Alarcón et al., 2019 [94] | Mexico | LC n3-PUFA vs placebo | 12 | 1200.0  EPA: 800.0  DHA: 400.0 | Not specified | 245 | 0 | 0 | Children |
| López-Alarcón et al., 2020 [95] | Mexico | LC n3-PUFA vs placebo | 12 | 1200.0  EPA: 800.0  DHA: 400.0 | Not specified | 117 | 0 | 117 | Children |
| Mitchell et al., 2021[96] | USA | Inulin vs placebo | 6 | 10000.0 | Not specified | 22 | 8 | 14 | Adults |
| Mosah et al., 2015 [97] | Iraq | L-carnitin vs placebo | 12 | 1000.0 | Not specified | 56 | 0 | 56 | Adults |
| Munro et al., 2011 [98] | Australia | LC n3-PUFA vs placebo | 8 | 6000.0  EPA: 1620.0  DHA: 420.0 | 6 | 33 | 11 | 22 | Adults |
| Munro et al., 2012 [99] | Australia | LC n3-PUFA vs placebo | 12 | 6000.0  EPA: 1620.0  DHA: 420.0 | 6 | 32 | 6 | 26 | Adults |
| Munro et al., 2013 [100] | Australia | LC n3-PUFA vs placebo | 12 | 6000.0  EPA: 420.0  DHA: 1620.0 | 6 | 39 | 9 | 30 | Adults |
| Neff et al., 2011 [101] | USA | LC n3-PUFA vs placebo | 3 | DHA: 2000.0 | oil | 36 | 0 | 0 | Adults |
| Novin et al., 2018 [102] | Iran | Vitamin B vs placebo | 4 | 40 | 1 | 42 | 0 | 46 | Adults |
| Oscarsson et al., 2018 [103] | Sweden | LC n3-PUFA vs placebo | 12 | 3400.0  EPA: 2400.0  DHA: 1000.0 | 4 | 42 | 30 | 21 | Adults |
| Pacifico et al., 2015 [104] | Italy | LC n3-PUFA vs placebo | 24 | DHA: 250.0 | 1 | 51 | 30 | 28 | Children |
| Parker et al., 2019 [105] | Australia | LC n3-PUFA vs placebo | 12 | 1728.0  EPA: 588.0  DHA: 412.0 | oil | 50 | 50 | 0 | Adults |
| Pauls et al., 2021 [106] | Canada | LC n3-PUFA vs placebo | 4 | DHA: 3860.0 | 7 | 43 | 0 | 43 | Adults |
| Rajkumar et al., 2014 [107] | India | LC n3-PUFA vs placebo | 6 | 300.0  EPA: 180.0  DHA: 120.0 | 1 | 60 | 30 | 30 | Adults |
| Root et al., 2013 [108] | USA | LC n3-PUFA vs placebo | 4 | 1740.0  EPA: 1050.0  DHA: 690.0 | 3 | 51 | 0 | 0 | Adults |
| Salman et al., 2022 [109] | Turkey | LC n3-PUFA vs placebo | 12 | 1020.0  EPA: 580.0  DHA: 390.0 | Not specified | 40 | 12 | 28 | Adults |
| Salmean et al., 2019 [110] | Kuwait | Inulin vs placebo | 6 | 21000.0 | 1 | 0 | 0 | 0 | Adults |
| Samimi et al., 2016 [111] | Iran | L-carnitin vs placebo | 12 | 250.0 | 1 | 60 | 0 | 60 | Adults |
| Sarbolouki et al., 2013 [112] | Iran | LC n3-PUFA vs placebo | 12 | EPA: 2000.0 | 4 | 67 | 26 | 41 | Adults |
| Shabrina et al., 2020 [113] | Taiwan | LC n3-PUFA vs placebo | 12 | 2130.0  EPA: 1280.0  DHA: 850.0 | oil | 21 | 21 | 0 | Adults |
| Sidiartha et al., 2017 [114] | Indonesia | LC n3-PUFA vs placebo | 8 | 500.0  EPA: 90.0  DHA: 450.0 | 3 | 70 | 37 | 33 | Children |
| Simao et al., 2012 [115] | Brazil | LC n3-PUFA vs placebo | 12 | 3000.0  EPA: 1800.0  DHA: 1200.0 | oil | 65 | 0 | 34 | Adults |
| Spencer et al., 2013 [116] | USA | LC n3-PUFA vs placebo | 12 | 4000.0  EPA: 1860.0  DHA: 1500.0 | Not specified | 0 | 11 | 22 | Adults |
| Tapsell et al., 2013 [117] | Australia | LC n3-PUFA vs placebo | 48 | 810.0  EPA: 420.0  DHA: 210.0 | oil | 64 | 0 | 0 | Adults |
| Utami et al., 2017 [118] | Taiwan | LC n3-PUFA vs placebo | 8 | 2130.0  EPA: 1250.0  DHA: 850.0 | oil | 71 | 0 | 71 | Adults |
| Utami et al., 2021 [119] | Indonesia | Inulin vs placebo | 8 | 240.0 | 2 | 55 | 21 | 34 | Adults |
| Vaghef-Mehrabany et al., 2019 [120] | Iran | Inulin vs placebo | 8 | 10000.0 | Not specified | 62 | 0 | 62 | Adults |
| Torres-Vanegas et al., 2025 [121] | Mexico | LC n3-PUFA vs placebo | 8 | 1800.0  EPA: 1020.0  DHA: 780.0 | Supplementation through meals | 40 | 24 | 16 | Adults |
| Veleba et al., 2015 [122] | Czech Republic | LC n3-PUFA vs placebo | 24 | 5000.0  EPA: 750.0  DHA: 2000.0 | 2 | 60 | 0 | 0 | Adults |
| Wang et al., 2016 [123] | China | LC n3-PUFA vs placebo | 24 | 2410.0  EPA: 1340.0  DHA: 1070.0 | 4 | 99 | 35 | 64 | Adults |
| Yang et al., 2011 [124] | Taiwan | Inulin vs placebo | 6 | 18000.0 | Powder | 30 | 16 | 14 | Adults |
| Zakharova et al., 2023 [125] | Russian Federation | L-carnitin vs placebo | 12 | 2000.0 | 1 | 90 | 56 | 34 | Adults |

# **Table S4.** Prediction intervals for the comparisons between nutraceuticals interventions with the placebo interventions.

|  | L-carnitin vs placebo | LC n3-PUFA vs placebo | Inulin vs placebo | Butyrate vs placebo | Vitamin B complex vs placebo |
| --- | --- | --- | --- | --- | --- |
|  | Mean (95% prediction intervals) | Mean (95% prediction intervals) | Mean (95% prediction intervals) | Mean (95% prediction intervals) | Mean (95% prediction intervals) |
| Weight | -5.13 (-6.55, -3.72) | -0.45 (-1.66, 0.76) | -1.67 (-3.14, -0.19) | // | -0.79 (-2.40, 0.82) |
| BMI | -2.04 (-2.64, -1.45) | -0.10 (-0.60, 0.39) | -0.68 (-1.36, 0.00) | -0.31 (-0.99, 0.37) | -0.30 (-0.97, 0.37) |
| Waist circumference | -1.75 (-4.33, 0.83) | -0.80 (-2.72, 1.13) | -1.60 (-4.59, 1.39) | -5.08 (-8.54, -1.62) | -1.55 (-4.10, 1.00) |
| Hip circumference | -2.14 (-4.50, 0.23) | -0.81 (-2.65, 1.03) | -0.78 (-5.40, 3.83) | // | // |
| Fat mass | -1.44 (-4.17, 1.29) | -0.44 (-1.32, 0.44) | -0.55 (-2.79, 1.69) | // | // |
| Fat free mass | 0.24 (-0.38, 0.86) | 0.04 (-0.26, 0.33) | 0.01 (-0.55, 0.56) | // | 0.07 (-0.73, 0.86) |
| Fasting glucose | -2.60 (-4.69, -0.50) | -0.23 (-2.09, 1.64) | -0.10 (-2.10, 1.90) | // | -5.13 (-8.02, -2.23) |
| Fasting insulin | -1.78 (-3.86, 0.29) | -0.19 (-2.00, 1.62) | -0.38 (-2.45, 1.69) | // | 1.07 (-1.52, 3.66) |
| HOMA-IR | -3.86 (-6.77, -0.94) | -0.26 (-2.77, 2.25) | -0.47 (-3.32, 2.38) | // | // |
| Triglycerides | -2.79 (-4.84, -0.74) | -0.72 (-2.56, 1.12) | 0.07 (-1.97, 2.12) | // | // |
| LDL-C | -1.05 (-2.75, 0.65) | 0.36 (-1.16, 1.88) | -0.22 (-1.91, 1.48) | // | 0.09 (-2.11, 2.29) |
| HDL-C | 2.45 (0.60, 4.30) | 0.03 (-1.62, 1.68) | -0.26 (-2.10, 1.58) | // | -0.39 (-2.77, 1.99) |
